# Exploration of piperidine 3D fragment chemical space: synthesis and 3D shape analysis of fragments derived from 20 regio- and diastereoisomers of methyl substituted pipecolinates†

**DOI:** 10.1039/d2md00239f

**Published:** 2022-10-11

**Authors:** S. Paul Jones, James D. Firth, Mary C. Wheldon, Masakazu Atobe, Roderick E. Hubbard, David C. Blakemore, Claudia De Fusco, Simon C. C. Lucas, Stephen D. Roughley, Lewis R. Vidler, Maria Ann Whatton, Alison J.-A. Woolford, Gail L. Wrigley, Peter O'Brien

**Affiliations:** Department of Chemistry, University of York Heslington York YO10 5DD UK peter.obrien@york.ac.uk; Asahi Kasei Pharma Corporation 632-1 Mifuku, Izunokuni Shizuoka 410-2321 Japan; Vernalis (R&D) Ltd. Granta Park, Abington Cambridge CB21 6GB UK; Medicine Design, Pfizer Inc. 445 Eastern Point Road Groton CT 06340 USA; Bayer AG, Research and Development, Pharmaceuticals, Synthetic Modalities 13353 Berlin Germany; Hit Discovery, Discovery Sciences, R&D, AstraZeneca Cambridge CB4 0WG UK; Amphista Therapeutics The Cori Building, Granta Park, Great Abington Cambridge CB21 6GQ UK; Evotec (UK) Ltd Dorothy Crowfoot Hodgkin Campus, 114 Innovation Drive, Milton Park, Abingdon Oxon OX14 4RZ UK; Astex Pharmaceuticals 436 Cambridge Science Park, Milton Road Cambridge CB4 0QA UK; Medicinal Chemistry, Oncology R&D, AstraZeneca Cambridge UK

## Abstract

Fragment-based drug discovery is now widely adopted for lead generation in the pharmaceutical industry. However, fragment screening collections are often predominantly populated with flat, 2D molecules. Herein, we report the synthesis of piperidine-based 3D fragment building blocks – 20 regio- and diastereoisomers of methyl substituted pipecolinates using simple and general synthetic methods. *cis*-Piperidines, accessed through a pyridine hydrogenation were transformed into their *trans*-diastereoisomers using conformational control and unified reaction conditions. Additionally, diastereoselective lithiation/trapping was utilised to access *trans*-piperidines. Analysis of a virtual library of fragments derived from the 20 *cis*- and *trans*-disubstituted piperidines showed that it consisted of 3D molecules with suitable molecular properties to be used in fragment-based drug discovery programs.

## Abstract 2

The synthesis and 3D shape analysis of fragments derived from 20 regio- and diastereoisomers of methyl substituted pipecolinates is described.

## Graphical abstract

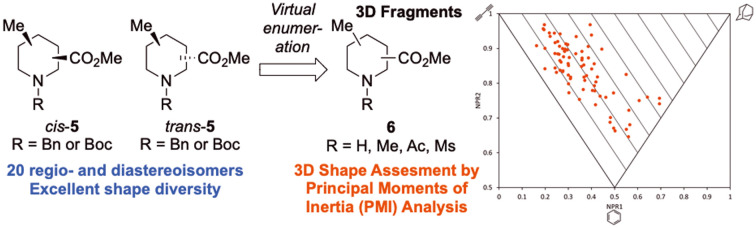

## Introduction

Fragment based drug discovery (FBDD) is now one of the main approaches for drug discovery in both pharmaceutical and academic laboratories.^1^ Over the last few years, six marketed drugs are listed as having their origins in FBDD methodology.^2–7^ Fragment sized chemical space can be broadly categorized by the ‘rule-of-three’: MW (molecular weight) < 300 Da; Clog *P* < 3; number of hydrogen bond acceptors (HBA) and donors (HBD) ≤ 3.^8^ Recently, Astex have adopted a narrower definition of fragment space with MW 140–230 Da (approximately 10–16 heavy atoms) and Clog *P* 0–2.^9^ The relatively low MW of fragments means that there is a more efficient sampling of chemical space and so fragment libraries are much smaller in size than typical high-throughput screening libraries.^10^ We^11^ and others^12^ have become increasingly interested in the exploration of 3D fragment chemical space. To some degree, the developing interest in 3D fragments can be traced back to Lovering *et al.*'s ‘Escape from Flatland’ paper,^13^ even though this work was not specifically concerned with fragment space and utilised Fsp^3^ (fraction of sp^3^ hybridised carbon atoms) as a metric (which we have recently shown is not a particularly good descriptor for 3D shape^11*c*^).

**Fig. 1:**
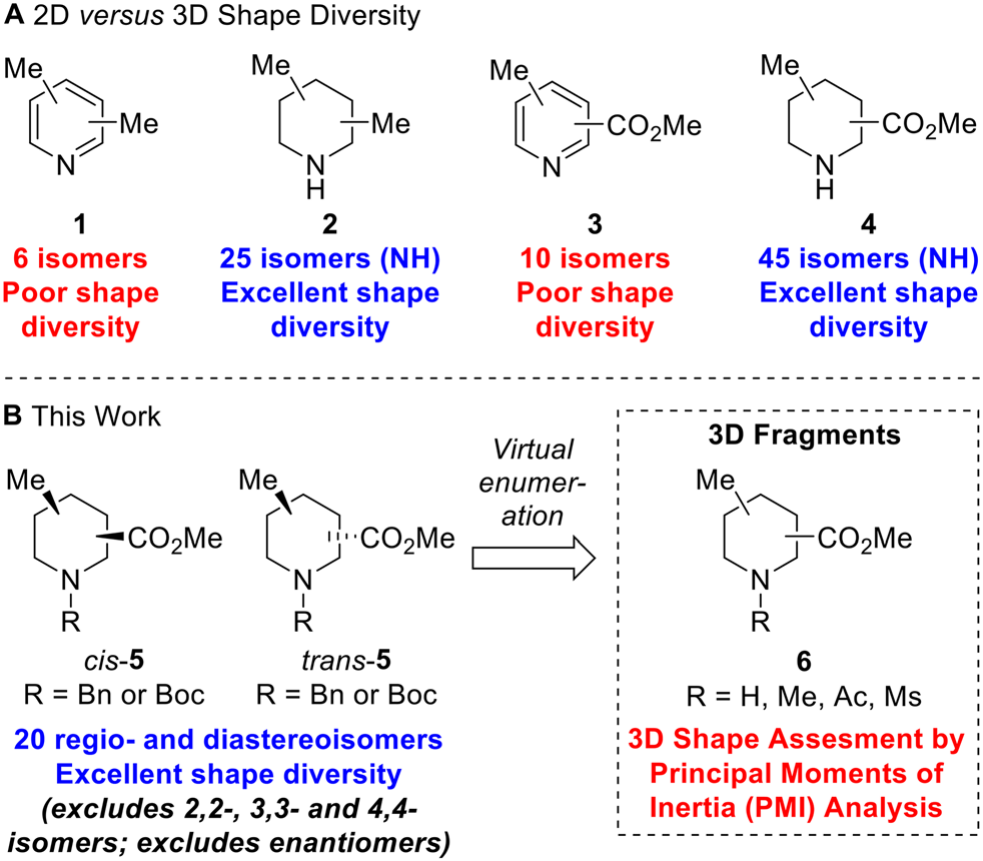
2D *versus* 3D shape diversity in disubstituted pyridines and piperidines and virtual 3D fragments presented in this work.

To illustrate the structural and shape diversity offered by ‘saturation’ of a scaffold, without significantly increasing its overall MW, Lovering *et al.* highlighted the isomeric composition of dimethyl pyridine 1*versus* dimethyl piperidine 2 (Fig. 1A).^13^ For dimethyl pyridine 1, there are six isomers which are of 2D shape but do at least provide different regioisomeric vectors for further functionalisation. In contrast, for dimethyl piperidine 2 (including *N*-methyl compounds), Lovering indicated that there were 34 isomers. As it turns out, this was incorrect as some of the depicted isomers were in fact the same compounds – there are 30 isomers which reduces to 25 isomers if we consider only NH piperidine compounds (Fig. 1A). Whichever number of isomers is considered, ‘saturation’ clearly provides a significant increase in shape diversity and complexity with only a small increase in MW (of 6). The 25 isomers of dimethyl NH piperidines 2 (see ESI†) comprise five achiral compounds (two of which are *meso*) and ten enantiomeric pairs of chiral compounds (*ci*s- and *trans*-configurations in four cases).

Given our interest in 3D fragments, we extended Lovering's analysis of pyridines and NH piperidines by furnishing the scaffolds with two different substituents: a methyl group and a methyl ester. In this case, the increase in 3D shape diversity was even more pronounced going from a 2D to a 3D scaffold: there are ten 2D isomers for methyl picolinates 3 and 45 3D isomers for methyl pipecolinates 4 (Fig. 1A). Of the 45 isomers for methyl pipecolinates 4, five are 2,2-, 3,3- or 4,4-disubstituted piperidines and there are two enantiomeric series of 20 *cis*- and *trans*-disubstituted piperidines. We considered that these 20 racemic *cis*- and *trans*-disubstituted methyl pipecolinates 4 would be interesting building blocks for use in medicinal chemistry and could form the key scaffold of novel 3D fragments (with *N*-functionalisation providing an additional point of diversity). Indeed, such piperidine motifs have found utility in drug development programs, including the development of PDK1 inhibitors^14^ and orexin receptor antagonists.^15^ However, for such 3D shape diversity to be realised in practice, viable synthetic routes to all possible isomers would be needed since a number of these substitution patterns had not been reported previously.

In this paper, we report three distinct pieces of synthetic methodology that has allowed access to each of the 20 regio- and diastereoisomers of methyl substituted pipecolinates *cis*- and *trans*-5 where the *N*-substituent is protected with either a benzyl or Boc group (Fig. 1B). It was not necessary to include the 2,2-, 3,3- or 4,4-disubstituted piperidines in this study since we had previously reported the synthesis of each of the *N*-Boc methyl pipecolinates (*via* enolate methylation).^11*c*^ Our synthetic approach towards the 20 regio- and diastereoisomers of methyl substituted pipecolinates *cis*- and *trans*-5 utilises pyridine hydrogenation and base-mediated epimerisation (*via* enolate formation) as well as Boc-directed α-lithiation-trapping (*vide infra*). This is the first report of the systematic synthesis of each isomer of methyl substituted pipecolinates *cis*- and *trans*-5 and means that fragments based on such piperidines can now be considered in drug discovery programmes. To highlight the 3D shape diversity provided by these 20 piperidines, each was enumerated with NH, NMe, *N*-acetamide and *N*-mesyl groups to generate a virtual library of 80 synthetically accessible 3D fragments 6, and their 3D shape was assessed using a principal moment of inertia (PMI) plot.^16^ Herein, we present our results.

## Results and discussion

**Scheme 1:**
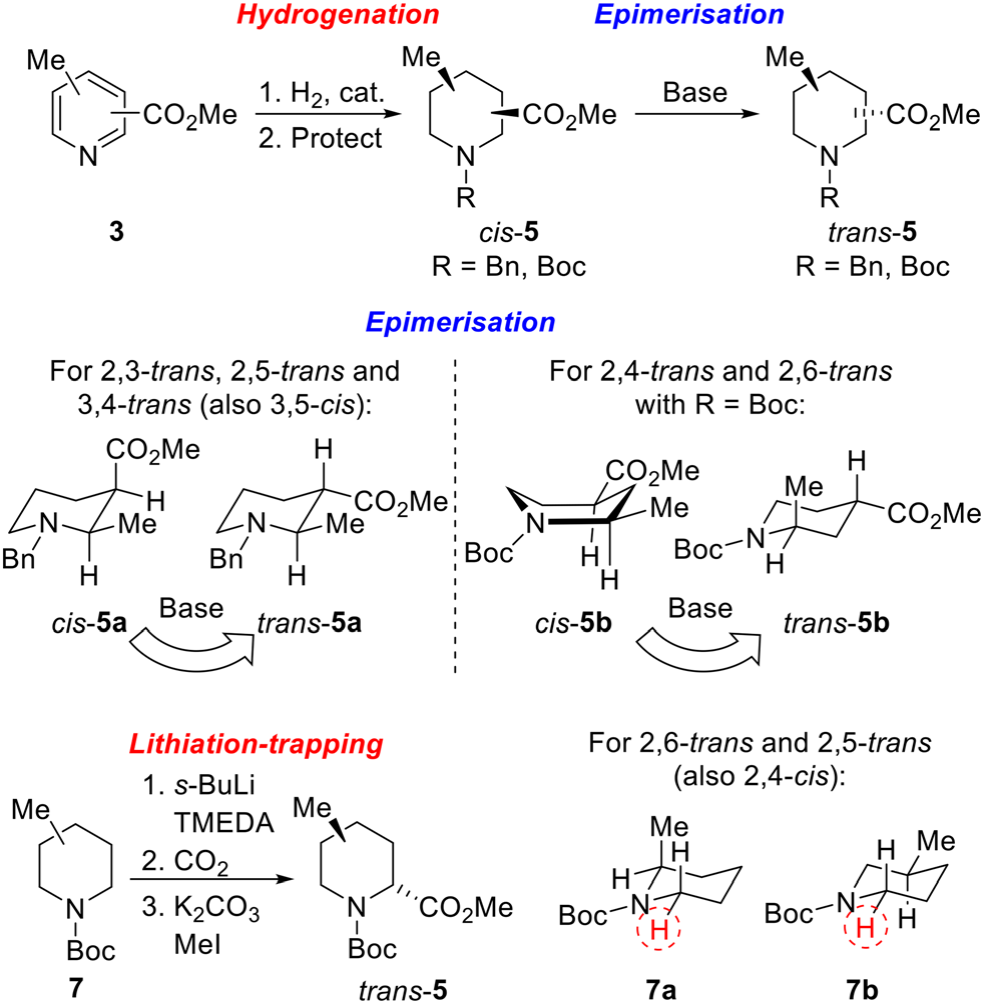
Synthetic strategy towards 20 regio- and diastereoisomers of methyl substituted pipecolinates: hydrogenation and epimerisation.

Our strategy for the synthesis of all 20 regio- and diastereoisomers of methyl substituted pipecolinates *cis*- and *trans*-5 (R = benzyl or Boc) is set out in Scheme 1. It was envisaged that hydrogenation of disubstituted pyridines 3 and subsequent *N*-protection would deliver methyl substituted pipecolinates *cis*-5 diastereoselectively.^17^ Then, subsequent base-mediated epimerisation of piperidines *cis*-5 (*via* enolate formation and re-protonation) should deliver diastereoisomeric piperidines *trans*-5. However, the epimerisation stage is not as straightforward as might appear and we anticipated that judicial choice of the *N*-protecting group would allow us to carry out the necessary epimerisations. For example, with 2,3-disubstitued *N*-benzyl piperidine *cis*-5a, one substituent will be axial and one equatorial. Thus, epimerisation of *cis*-5a under thermodynamic conditions should generate *trans*-5a driven by relieving the unfavourable 1,3-diaxial interaction (Scheme 1). Using a *N*-benzyl group, this strategy should be successful for the preparation of 2,3-*trans*, 2,5-*trans* and 3,4-*trans* isomers (together with 3,5-*cis*).

In contrast, when R = Boc in methyl pipecolinates, there are different effects which control the lowest energy conformations and this would impact on the epimerisation event. For example, with 2,4-disubstitued *N*-Boc piperidine *cis*-5b, the lowest energy conformation will likely be a twist-boat conformation with both substituents in pseudo-equatorial positions.^18^ In contrast, the epimeric 2,4-disubstituted *N*-Boc piperidine *trans*-5b will adopt a lower energy conformation, with an equatorial 4-substituent and the 2-substituent adopting an axial orientation to avoid unfavourable A^1,3^-type strain between the Boc group and the 2-substituent.^18*a*^ Thus, base-mediated epimerisation under thermodynamic conditions should convert *cis*-5b into *trans*-5b (Scheme 1). With a *N*-Boc substituent, this approach should be successful for the formation of 2,4-*trans* and 2,6-*trans* isomers. Clearly, either benzyl or Boc groups would be suitable for thermodynamically driven epimerisation of piperidines without 2-substituents (*i.e.* to 3,4-*trans* isomers).

Finally, an alternative route to selected *trans*-isomers would be the use of Beak's α-lithiation-trapping methodology,^18*a*,19^ with which we have much experience.^11*a*,20^ For this, it was envisaged starting with *N*-Boc methyl piperidines 7 and carrying out α-lithiation (using *s*-BuLi/TMEDA) and subsequent trapping with carbon dioxide (followed by methylation) to give methyl pipecolinates *trans*-5 (Scheme 1). For *N*-Boc 2-methyl piperidine 7a, equatorial lithiation from the lowest energy conformation with an axial 2-methyl group (to avoid unfavourable A^1,3^-type strain) should give the 2,6-*trans* isomer.^19*a*^ In contrast, for *N*-Boc 3-methyl piperidine 7b, the lowest energy conformation will have an equatorial methyl group (to avoid 1,3-diaxial interactions) and equatorial lithiation should then deliver the 2,5-*trans* isomer.^18*a*^ A similar rationale would also allow access to the 2,4-*cis*-isomer if desired.

Although the transition metal catalysed hydrogenation of several regioisomers of disubstituted pyridines 3 is known,^21^ no general methods exist. These reductions are often carried out at high pressure and/or high temperature and use high catalyst loadings or mixed metal catalyst systems. Furthermore, in many cases, specialist equipment such as Parr reactors, microwaves^21*b*^ or flow equipment^21*c*^ is required. We wanted to identify a general and simple hydrogenation process in which all 10 possible isomers of 3 could be reduced without the need for especially high catalyst loadings or specialist equipment.

**Scheme 2:**
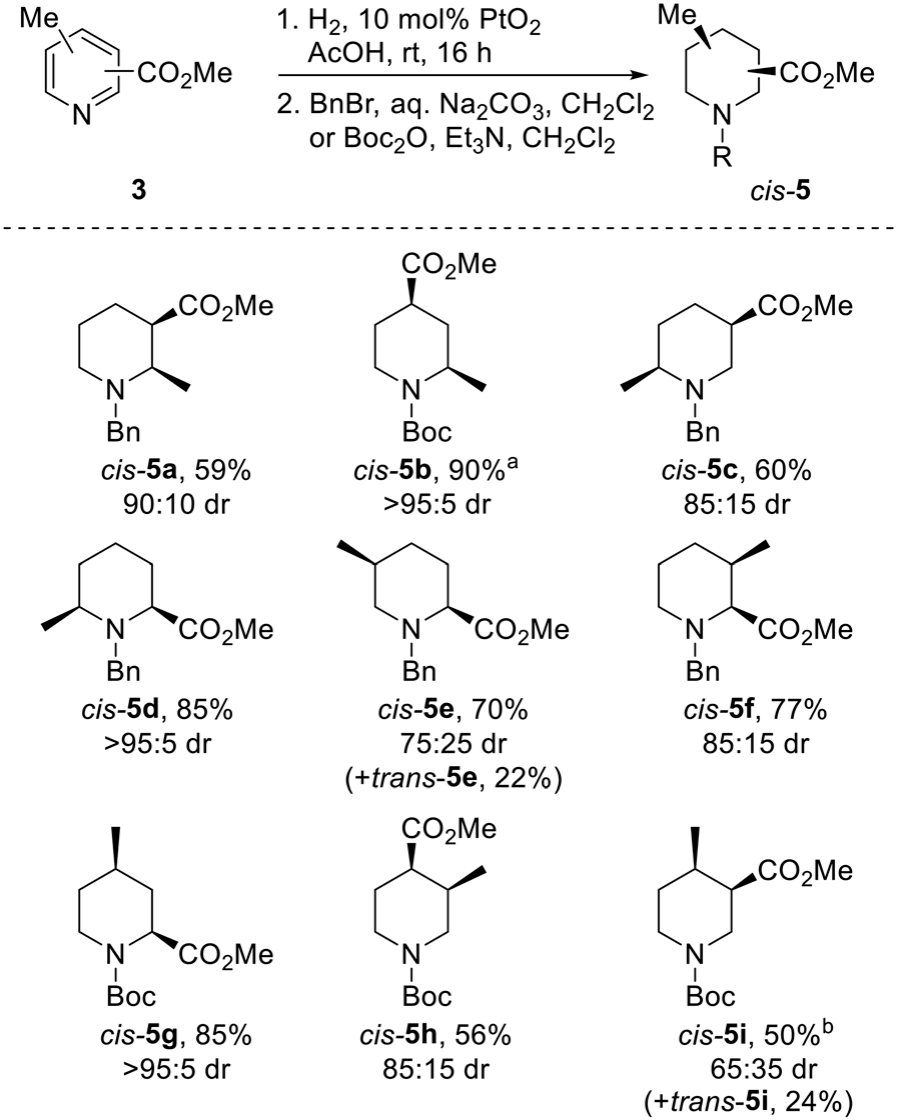
Reduction of pyridines 3 to piperidines *cis*-5. All products are racemic and the drs reported are the ratio of diastereoisomers obtained from the ^1^H NMR spectrum of the crude reaction product. ^*a*^ Methyl 2-chloro-6-methylpyridine-4-carboxylate was used; ^*b*^ 30 mol% PtO_2_ used.

The use of PtO_2_ as a hydrogenation catalyst was initially explored. To our delight, use of a comparatively low loading of 10 mol% PtO_2_ and a balloon of hydrogen successfully reduced acetic acid solutions of all but one pyridine 3 in under 16 h; complete reduction of 3h required the use of 30 mol% PtO_2_. Following protection with either a benzyl or Boc group, piperidines *cis*-5 were isolated in 50–90% yields as single diastereomers (Scheme 2). In all but one case, protected piperidines *cis*-5 were the major products, being formed in 65 : 35 to >95 : 5 dr (the drs reported are the ratio of diastereoisomers obtained from the ^1^H NMR spectrum of the crude reaction product). Of particular note, 2,4-substituted systems *cis*-5b and *cis*-5g were isolated in 90% and 85% yield respectively, with each being formed in >95 : 5 dr.

**Scheme 3:**
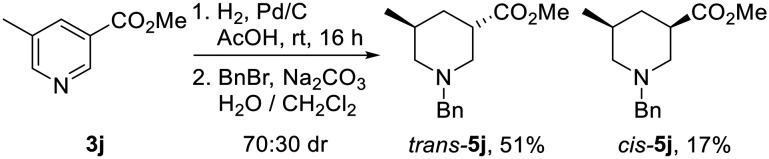
Reduction of pyridine 3j to piperidines *cis*-5. Both products are racemic and the dr reported is the ratio of diastereoisomers obtained from the ^1^H NMR spectrum of the crude reaction product.

Surprisingly, hydrogenation of 3,5-substituted pyridine 3j using 10% Pd/C led to preferential formation of the *trans*-isomer in 70 : 30 dr, with *trans*-5j and *cis*-5j being isolated in 51% and 17% yields after *N*-benzylation (Scheme 3). Use of 10% PtO_2_ resulted in 60 : 40 dr with *trans*-5j as the major product. Interestingly, Kappe has shown that the diastereoselectivity of the hydrogenation of 3j under flow conditions is dependent upon the catalyst employed, reaction temperature and pressure.^21*c*^ Given that *cis*-isomer *cis*-5j is likely to be the thermodynamically favoured product from an epimerisation experiment (*vide infra*), preferential formation of *N*-benzyl piperidine *trans*-5j*via* hydrogenation was fortuitous.

The relative stereochemistry of *cis*-5h was confirmed by single-crystal X-ray diffraction of the *N*-tosyl derivative.^11*c*^ The relative stereochemistry of all other *cis*-piperidines *cis*-5 was confirmed by comparison with known compounds or by analysis of *J* values in the ^1^H NMR spectra (see ESI† for full details).

**Scheme 4:**
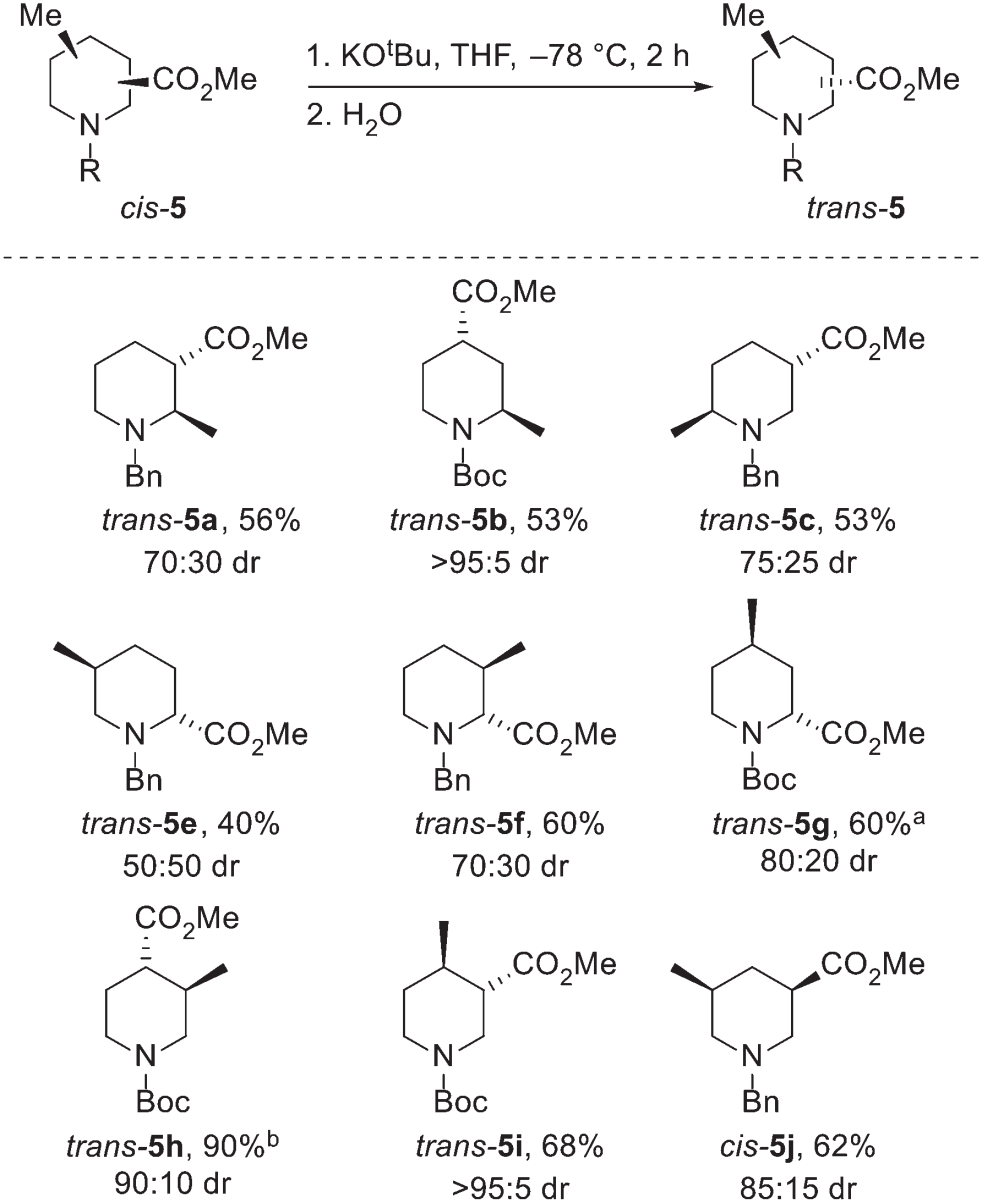
Epimerisation of di-substituted piperidines 5. All products are racemic and the drs reported, unless stated otherwise, are the ratio of diastereoisomers obtained from the ^1^H NMR spectrum of the crude reaction product. ^*a*^ LDA was used; ^*b*^ isolated as a 90 : 10 mixture of *trans*- and *cis*-isomers.

Next, the epimerisation of the ester groups of *cis*-piperidines *cis*-5 to the epimeric piperidines *trans*-5 (and of *trans*-5j to *cis*-5j) were explored. Isolated examples of the epimerisation of piperidines 5 have been reported,^21*d*,*i*,22^ but we were keen to identify general conditions that allowed access to all 10 possible epimers. Treatment of eight diastereomerically pure piperidines *cis*-5 with potassium *tert*-butoxide in THF at −78 °C for 2 h^21*i*^ resulted in epimerisation to give *trans*-piperidines *trans*-5, formed in 50 : 50–95 : 5 dr, isolated in 40–90% yields (Scheme 4). Of note, epimerisation of *cis*-5g to *trans*-5g required the use of LDA as a base due to a significant amount of trans-esterification when using potassium *tert*-butoxide. In this case, the observed 80 : 20 dr may be due to kinetic selectivity in the enolate re-protonation event. When 3,5-disubituted piperidine *trans*-5j was subjected to the standard conditions, *cis*-5j was formed in 85 : 15 dr and isolated in 62% yield.

Based on the analysis shown in Scheme 1, all of the epimerisations except that for piperidine *trans*-5e proceeded towards the expected thermodynamic product. Surprisingly, for *trans*-5e, a 50:50 mixture of *trans*- and *cis*-5e was generated, from which a 40% yield of *trans*-5e was obtained.

**Scheme 5:**
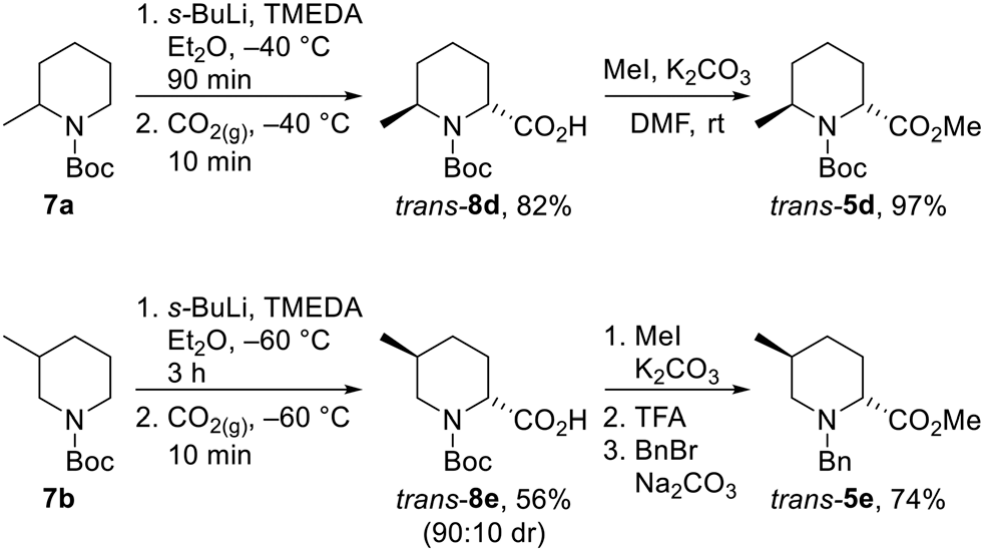
Synthesis of *trans*-5d and *trans*-5e*via* lithiation/trapping. All products are racemic and the dr of *trans*-8e reported is the ratio of diastereoisomers obtained from the ^1^H NMR spectrum of the crude reaction product and after chromatography.

Unfortunately, we were unable to install the requisite Boc group on to the *cis*-2,6-disubstituted system (*cf. cis*-5d) due to steric constraints. Therefore, the *trans*-2,6-system was not accessible using the epimerisation strategy and diastereoselective lithiation/trapping methodology was thus utilised to synthesise the single outstanding diastereomer *trans*-5d. Using a modification of Beak's conditions for the α-lithiation of substituted piperidines,^18*a*,19^*N*-Boc 2-methyl piperidine 7a was treated with *s*-BuLi and TMEDA in Et_2_O at −40 °C for 90 min. Subsequent trapping with CO_2_ gave 2,6-disubstitiuted piperidine *trans*-8d as a single regio- and diastereoisomer in 82% yield. Methylation of *trans*-8d gave piperidine *trans*-5d in 97% yield (Scheme 5).

Due to the poor diastereoselectivity (50 : 50 dr), and hence yield (40%) in the epimerisation of *cis*-5e to *trans*-5e (see Scheme 4), the lithiation/trapping methodology was explored as an alternative method for the synthesis of *trans*-5e.^22^ Treatment of *N*-Boc 3-methyl piperidine 7b with *s*-BuLi and TMEDA in Et_2_O at −60 °C for 3 h and subsequent trapping with CO_2_ gave an inseparable 90 : 10 mixture of *trans*-8e and *cis*-8e in 56% yield. Methylation followed by Boc group removal and *N*-benzylation resulted in *trans*-5e being isolated in 74% yield (41% overall from 6b) (Scheme 5).

**Fig. 2:**
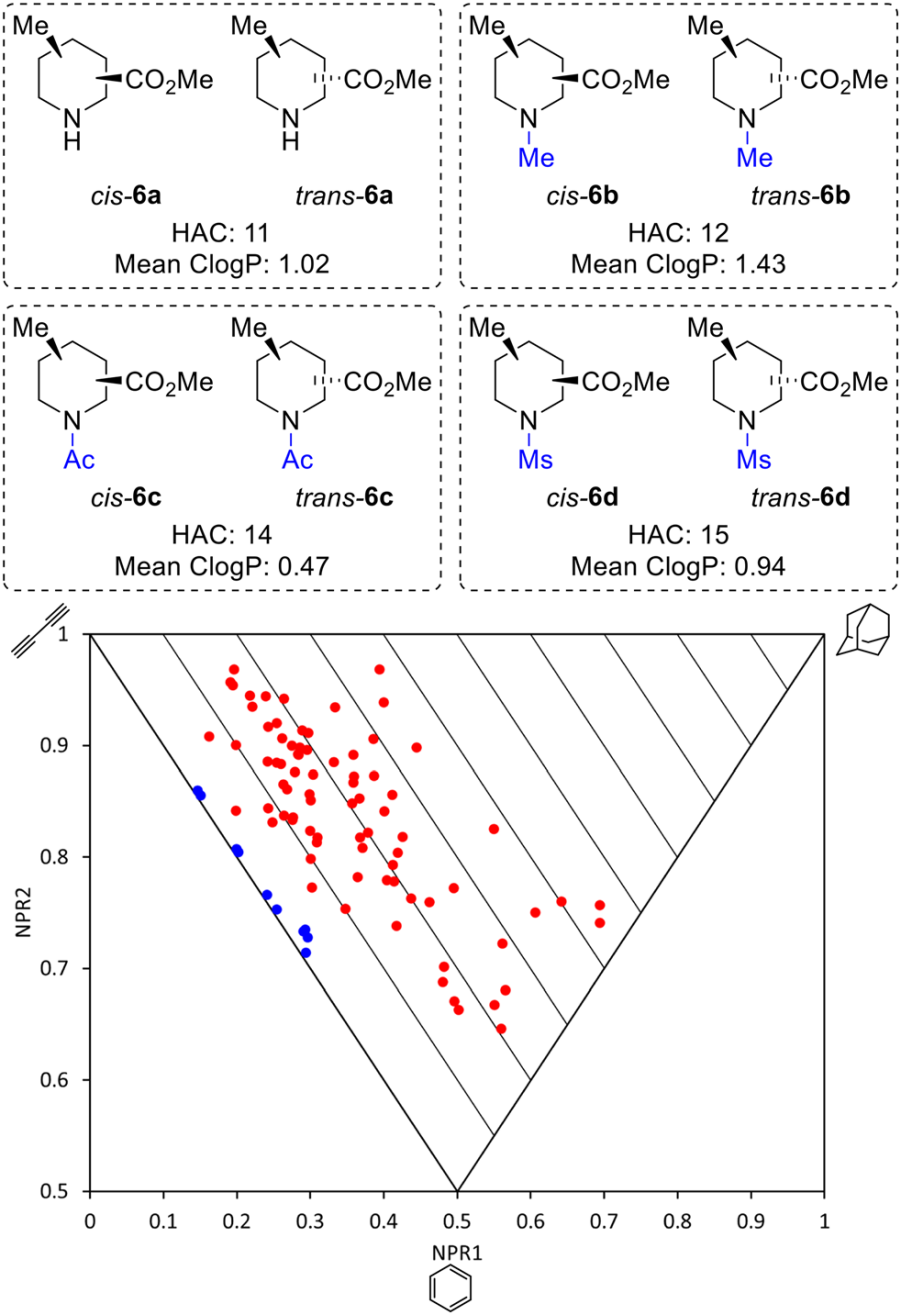
Molecular properties and PMI plot of the virtual fragment library: *cis*- and *trans*-6a–6d (red dots); pyridines 3 (blue dots) NPR is the normalised principal moment of inertia (see ESI†).

Thus, by using three different pieces of synthetic chemistry, all 20 regio- and diastereoisomers of methyl substituted picolinates 5 were prepared. Next, the suitability of the 20 piperidines 5 for the synthesis of 3D fragments for use in FBDD projects was assessed. First, we virtually removed the *N*-Boc or benzyl groups from 5 to give secondary amines 6a. Then, the amines 6a were virtually capped with one of three small groups (Me, Ac or Ms) to generate a virtual library of 80 fragments 6a–d (Fig. 2). The Me, Ac, and Ms substituents were selected as they are the simplest representatives of reductive amination, amide formation and sulfonamide formation reactions at the piperidine nitrogen atom, which are very commonly used in medicinal chemistry,^23^ and also provide mutually distinct exit vectors for substituents. Furthermore, Me, Ac, and Ms substituents featured in our previous 3D fragments^11*c*^ and thus provide a suitable comparison. In addition, methodology for attaching these substituents has been established in this previous work. Importantly, all fragments fit within Astex's updated guidelines for the physicochemical properties of fragments (HAC: 10–16 and log *P*: 0–2)^9^ (see ESI†). The 3D shape of the fragments was analysed using normalised principal moments of inertia (PMI) for the molecular mechanics-generated lowest energy conformation, as introduced by Sauer and Schwarz.^16^ The three vertices of these plots correspond to rod, disc and spherical shapes. Many current fragment libraries consist predominantly of flat (hetero)aromatic compounds that populate the rod-disc axis.^12*b*^ In comparison, our virtual fragments occupy a wide area of chemical space away from the rod-disc axis (Fig. 2, red dots), with 79 of the 80 fragments deemed to be 3D (by Firth's definition: NPR1 + NPR2 ≥ 1.07)^11*d*,24^ and would make suitable 3D fragments for a FBDD project. For comparison, the ten 2D isomers for methyl picolinates 3 lie on the rod-disc axis (Fig. 2, blue dots). Finally, the suitability of the piperidine fragment scaffolds as building blocks for medicinal chemistry was validated using the open-access tool LLAMA^25^ (see ESI† for full details).

## Conclusions

In summary, we have developed simple and practical methods for the synthesis of all 20 regio- and diastereoisomers of *cis*- and *trans*-methyl substituted pipecolinates 5. Architecturally simple and easily accessible pyridines 3 were hydrogenated under mild and practical conditions to generate more structurally complex piperidines *cis*-5 in moderate to excellent yields. Subsequent epimerisation, facilitated by conformational control, using general conditions gave all but one piperidines *trans*-5; the remaining isomer (*trans*-5d) was attained using diastereoselective lithiation/trapping methodology in excellent yield. Crucially, we have shown that a virtual library of fragments 6 derived from piperidines 5 are three-dimensional, cover a wide area of 3D fragment space and have excellent molecular properties for fragment-based drug discovery campaigns.

## Author contributions

All authors contributed to the conceptualization and writing (review & editing). SPJ, JDF, MCW, MA and POB carried out the investigation, methodology, data curation and formal analysis. POB and REH provided supervision and funding acquisition. POB and JDF carried out the writing (original draft).

## Conflicts of interest

There are no conflicts to declare.

## Supplementary Material

MD-013-D2MD00239F-s001
