# Supplementary material for: Exploration of piperidine 3D fragment chemical space: synthesis and 3D shape analysis of fragments derived from 20 regio- and diastereoisomers of methyl substituted pipecolinates
Source: RSC Med Chem. 2022 Oct 11;13(12):1614–20. doi: 10.1039/d2md00239f (PMC9749955; doi:10.1039/d2md00239f)
Supplement: MD-013-D2MD00239F-s001 [file MD-013-D2MD00239F-s001.pdf]

Electronic Supplementary Material (ESI) for RSC Medicinal Chemistry.
This journal is © The Royal Society of Chemistry 2022

Exploration of Piperidine 3D Fragment Chemical Space: Synthesis and 3D Shape Analysis of Fragments Derived from 20 Regio- and Diastereoisomers of Methyl Substituted Pipecolinates

S. Paul Jones,[a] James D. Firth,[a] Mary C. Wheldon,[a] Masakazu Atobe,[a,b] Roderick E. Hubbard,[a,c] David C. Blakemore,[d] Claudia De Fusco,[e] Simon C. C. Lucas,[f] Stephen D. Roughley,[c] Lewis R. Vidler,[g] Maria Ann Whatton,[h] Alison J.-A. Woolford,[i] Gail L. Wrigley[j] and Peter O'Brien*[a]

[a] Department of Chemistry, University of York, Heslington, York YO10 5DD, U.K.

[b] Asahi Kasei Pharma Corporation, 632-1 Mifuku, Izunokuni, Shizuoka 410-2321, Japan.

[c] Vernalis (R&D) Ltd, Granta Park, Abington, Cambridge, CB21 6GB, UK.

[d] Medicine Design, Pfizer Inc, 445 Eastern Point Road, Groton, CT 06340, USA.

[e] Current address: Bayer AG, Research and Development, Pharmaceuticals, Synthetic Modalities, 13353, Berlin, Germany.

[f] Hit Discovery, Discovery Sciences, R&D, AstraZeneca, Cambridge, CB4 0WG, UK.

[g] Current address: Amphista Therapeutics, The Cori Building, Granta Park, Great Abington, Cambridge CB21 6GQ.

[h] Current address: Evotec (UK) Ltd, Dorothy Crowfoot Hodgkin Campus, 114 Innovation Drive, Milton Park, Abingdon, Oxon, OX14 4RZ UK.

[i] Astex Pharmaceuticals, 436 Cambridge Science Park, Milton Road, Cambridge, CB4 0QA.

[j] Medicinal Chemistry, Oncology R&D, AstraZeneca, Cambridge, UK.

peter.obrien@york.ac.uk

Index

1. Experimental Details S3

1.1 General S3

1.2 General Procedures S4

1.3 Experimental Procedures and Characterisation Data S5

2. Isomers of dimethyl pyridine **1** and dimethyl NH piperidine **2** S35

3. Virtual Library Analysis S36

3.1 Principal Moments of Inertia and Molecular Properties S36

3.2 Virtual Library Enumeration and Lead-Likeness Analysis S37

4. ^{1}H and ^{13}C NMR Spectra S39

5. References S77

1. Experimental Details

1.1. General

All-non aqueous reactions were carried out under oxygen free Ar or N_2 using flame-dried glassware. Et_2O and THF were freshly distilled from sodium and benzophenone. Alkyllithiums were titrated against *N*-benzylbenzamide before use. Brine refers to a saturated solution. Water is distilled water.

Flash column chromatography was carried out using Fluka Chemie GmbH silica (220-440 mesh). Thin layer chromatography was carried out using commercially available Merck F_{254} aluminium backed silica plates. Proton (400 MHz) and carbon (100.6 MHz) NMR spectra were recorded on a Jeol ECX-400 instrument using an internal deuterium lock. For samples recorded in $CDCl_3$, chemical shifts are quoted in parts per million relative to $CHCl_3$ (δ_H 7.26) and $CDCl_3$ (δ_C 77.0, central line of triplet). Carbon NMR spectra were recorded with broad band proton decoupling and assigned using DEPT experiments. Coupling constants (*J*) are quoted in Hertz. Melting points were carried out on a Gallenkamp melting point apparatus. Infrared spectra were recorded on a Perkin Elmer UATR Two FT-IR spectrometer. Electrospray high and low resonance mass spectra were recorded at room temperature on a Bruker Daltronics microOTOF spectrometer.

1.2 General Procedures

General Procedure A: Pyridine methyl ester formation

Thionyl chloride (0.06-1.45 mL, 0.80-20.0 mmol, 1.1-3.0 eq.) was added dropwise over 5 min to a stirred solution of the carboxylic acid (0.73-10.0 mmol, 1.0 eq.) in MeOH (5-20 mL) at 0 °C under Ar. The resulting solution was stirred and heated at reflux for 1 h or 16 h. The mixture was then allowed to cool to rt and the solvent was evaporated under reduced pressure. And then the mixture was dissolved in CH_2Cl_2 (50 mL) and washed with saturated $NaHCO_{3(aq)}$ (10 mL). The organic layer was dried ($MgSO_4$) and evaporated under reduced pressure to give the crude product.

General procedure B: Pyridine hydrogenation and neutralization

PtO_2 (10-60 mg, 0.05-0.26 mmol, 10-30 mol%) or 10% Pd/C (70 mg, 0.07 mmol, 10 mol%) was added to a stirred solution of pyridine ester (0.45-2.64 mmol) in AcOH (1-5 mL) at rt under Ar. The reaction flask was evacuated under reduced pressure and back-filled with Ar three times. After the final evacuation, H_2 was charged and the reaction mixture was stirred vigorously under a balloon of H_2 for 24 h. The solids were removed by filtration through Celite and washed with MeOH (30 mL). The filtrate was evaporated under reduced pressure to give the crude product. The crude product was dissolved in CH_2Cl_2 (5 mL) and $NH_4OH_{(aq)}$ (2 mL) was added. The two layers were separated, and the aqueous layer was extracted with CH_2Cl_2 (3 × 50 mL). The combined organic layers were dried (Na_2SO_4) and evaporated under reduced pressure to give the crude product.

General Procedure C: Piperidine epimerisation *via* KO*t*Bu

N-Benzyl/*N*-Boc piperidine (50-173 mg, 0.20-0.70 mmol, 1.0 eq.) was added to a flask containing dry THF (3-5 mL) at rt under Ar. The reaction flask was evacuated under reduced pressure and back-filled with Ar three times and the solution was cooled to –78 °C. KO*t*Bu (0.24-0.84 mL of a 1 M solution in THF, 0.24-0.84 mmol, 1.2 eq.) was added dropwise. The resulting solution was stirred at –78 °C for 2 h. Then, water (1 mL) was added at –78 °C and the reaction mixture was allowed to warm to rt. The mixture was extracted with EtOAc (3 × 5 mL) and the combined organics were dried ($MgSO_4$) and evaporated under reduced pressure to give the crude product.

1.3 Experimental Procedures and Charterisation

Methyl 2-methylpyridine-3-carboxylate 3a

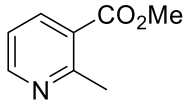

3a

Using general procedure A, thionyl chloride (0.77 mL, 10.60 mmol, 3.0 eq.) and 2-methyl-nicotinic acid (485 mg, 3.54 mmol, 1.0 eq.) in MeOH (20 mL) gave pyridine **3a** (443 mg, 82%) as an orange oil, IR (ATR) 1722 (C=O), 1571, 1433, 1278, 1084, 754 cm^{-1}; ^{1}H NMR (400 MHz, $CDCl_3$) δ 8.61 (dd, *J* = 5.0, 2.0 Hz, 1H, Ar), 8.19 (dd, *J* = 8.0, 2.0 Hz, 1H, Ar), 7.21 (dd, *J* = 8.0, 5.0 Hz, 1H, Ar), 3.92 (s, 3H, OMe), 2.83 (s, 3H, Me); ^{13}C NMR (100.6 MHz, $CDCl_3$) δ 167.1 (C=O), 160.1 (*ipso*-Ar), 152.0 (Ar), 138.6 (Ar), 125.5 (*ipso*-Ar), 121.0 (Ar), 52.4 (OMe), 25.0 (Me); HRMS *m/z* calcd for $C_8H_9NO_2$ (M + H)$^+$ 152.0706, found 152.0703 (+1.8 ppm error).

Lab Book – PJ-05-50

Methyl (2*R**,3*R**)-2-methylpiperidine-3-carboxylate *cis*-4a and methyl (2*R**,3*S**)-2-methylpiperidine-3-carboxylate *trans*-4a

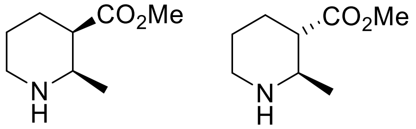

cis-**4a** *trans*-**4a**

Using general procedure B, PtO_2 (10 mg, 0.05 mmol, 10 mol%) and pyridine **3a** (70 mg, 0.45 mmol, 1.0 eq.) in AcOH (5 mL) gave the crude product which contained a 90:10 mixture of piperidines *cis*-**4a** and *trans*-**4a** (65 mg, 93%) as a yellow oil, ^{1}H NMR (400 MHz, $CDCl_3$) δ 3.68 (s, 3H, OMe), 3.10-3.03 (m, 1H, NCH), 2.96 (qd, *J* = 7.0, 3.5 Hz, 1H, C*H*Me), 2.66 (ddd, *J* = 13.5, 10.0, 3.5 Hz, 1H, NCH), 2.56-2.51 (m, 1H, $CHCO_2$), 2.02-1.94 (m, 1H, CH), 1.80-1.71 (m, 1H, CH), 1.71-1.60 (m, 2H, CH and NH), 1.42-1.33 (m, 1H, CH), 1.11 (d, *J* = 7.0 Hz, 2.7H, CH*Me*), 1.06 (d, *J* = 6.0 Hz, 0.3H); ^{13}C NMR (100.6 MHz, $CDCl_3$) for *cis*-**4a**: δ 174.8 (C=O), 52.4 (*C*HMe), 51.3 (OMe), 45.3 (NCH_2), 44.1

($CHCO_2$), 26.4 (CH_2), 22.8 (CH_2), 19.2 (CH*Me*). Spectroscopic data consistent with those reported in the literature.[1]

Lab Book – PJ-05-51

The relative stereochemistry of *cis*-**4a** and *trans*-**4a** has been proven unambiguously in our previous work.[2]

Methyl (2*R,3*R**)-1-benzyl-2-methylpiperidine-3-carboxylate *cis*-5a and methyl (2*R**,3*S**)-1-benzyl-2-methylpiperidine-3-carboxylate *trans*-5a**

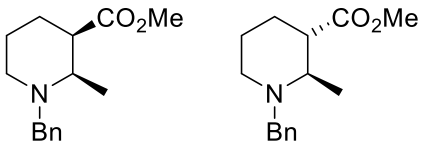

cis-**5a** *trans*-**5a**

BnBr (0.14 mL, 1.20 mmol, 3 eq.) was added dropwise to a 90:10 solution of piperidine *cis*-**4a** and *trans*-**4a** (63 mg, 0.40 mmol, 1.0 eq.) in 1:1 CH_2Cl_2-sat. $Na_2CO_{3(aq)}$ (4 mL) at rt under Ar. The resulting solution was stirred at rt for 16 h. The two layers were separated, and the aqueous layer was extracted with CH_2Cl_2 (4 × 10 mL). The combined organics were dried ($MgSO_4$) and evaporated under reduced pressure to give the crude product as a colourless oil. Purification by flash column chromatography on silica with 80:20 hexane-EtOAc as eluent gave *N*-benzyl piperidine *cis*-**5a** (63 mg, 64%) as a colourless oil, R_F (80:20 hexane-EtOAc) 0.13; IR (ATR) 2943, 1732 (C=O), 1138, 732, 697 cm^{-1}; ^1H NMR (400 MHz, $CDCl_3$) δ 7.36-7.28 (m, 4H, Ph), 7.26-7.21 (m, 1H, Ph), 3.66 (d, J = 13.5 Hz, 1H, NC*H*Ph), 3.65 (s, 3H, OMe), 3.54 (d, J = 13.5 Hz, 1H, NC*H*Ph), 3.42 (qd, J = 6.5, 5.0 Hz, 1H, NC*H*Me), 2.84 (ddd, J = 12.5, 5.0, 5.0 Hz, 1H, $CHCO_2$), 2.48-2.37 (m, 2H, NCH), 1.82-1.73 (m, 1H, CH), 1.73-1.58 (m, 2H, CH), 1.56-1.45 (m, 1H, CH), 0.91 (d, J = 6.5 Hz, 3H, CH*Me*); ^{13}C NMR (100.6 MHz, $CDCl_3$) δ 174.7 (C=O), 139.8 (*ipso*-Ph), 128.6 (Ph), 128.4 (Ph), 126.9 (Ph), 59.3 (NCH$_2$Ph), 54.1 (N*C*HMe), 51.6 (OMe), 46.1 (*C*HCO$_2$), 44.6 (NCH_2), 24.5 (CH_2), 20.7 (CH_2), 6.2 (CH*Me*); HRMS *m*/*z* calcd for $C_{15}H_{21}NO_2$ $(M + H)^+$ 248.1645, found 248.1652 (–2.0 ppm error).

Lab Book – PJ-05-53.

BnBr (0.23 mL, 1.91 mmol, 1.2 eq.) was added dropwise to a 90:10 solution of piperidine *cis*-**4a** and *trans*-**4a** (250 mg, 1.59 mmol, 1.0 eq.) in 1:1 CH_2Cl_2-sat $Na_2CO_{3(aq)}$ (6 mL) at rt under Ar. The resulting solution was stirred at rt for 16 h. The two layers were separated, and the aqueous layer was extracted with CH_2Cl_2 (4 × 10 mL). The combined organics were dried ($MgSO_4$) and evaporated under reduced pressure to give the crude product as a colourless oil. Purification by flash column chromatography on silica with 80:20 hexane-EtOAc as eluent gave a 90:10 mixture *N*-benzyl piperidine *cis*-**5a** and *N*-benzyl piperidine *trans*-**5a** (274 mg, 70%) as a colourless oil.

Lab Book – PJ-06-18.

Using general procedure C, a 90:10 mixture of *N*-benzyl piperidine *cis*-**5a** and *trans*-**5a** (150 mg, 0.61 mmol, 1.0 eq.) and KO*t*Bu (0.73 mL of a 1 M solution in THF, 0.73 mmol, 1.2 eq.) in THF (5 mL) gave the crude product as an orange oil. Purification by flash column chromatography on silica with 90:10 hexane-EtOAc as eluent gave an 80:20 mixture of *N*-benzyl piperidine *cis*-**5a** and *trans*-**5a** (61 mg, 41%) and *N*-benzyl piperidine *trans*-**5a** (84 mg, 56%) as a colourless oil, R_F (80:20 hexane-Et_2O) 0.19; IR (ATR) 2943, 2795, 1732 (C=O), 1434, 1138, 732, 697 cm^{-1}; ^{1}H NMR (400 MHz, $CDCl_3$) δ 7.32-7.27 (m, 4H, Ph), 7.24-7.20 (m, 1H, Ph), 3.95 (d, *J* = 13.5 Hz, 1H, NC*H*Ph), 3.67 (s, 3H, OMe), 3.25 (d, *J* = 13.5 Hz, 1H, NC*H*Ph), 2.76-2.68 (m, 2H, NC*H*Me and NCH), 2.35 (ddd, *J* = 10.0, 9.0, 4.0 Hz, 1H, $CHCO_2$), 2.12-2.04 (m, 1H, NCH), 1.88-1.81 (m, 1H, CH), 1.65-1.56 (m, 2H, CH), 1.51-1.44 (m, 1H, CH), 1.15 (d, *J* = 6.0 Hz, 3H, CH*Me*); ^{13}C NMR (100.6 MHz, $CDCl_3$) δ 175.7 (C=O), 139.5 (*ipso*-Ph), 129.0 (Ph), 128.3 (Ph), 126.9 (Ph), 57.6 (N$C$$H_2$Ph), 57.2 (*C*HMe), 51.7 (OMe), 51.2 (NCH_2), 49.5 (*C*HCO_2), 27.3 (CH_2), 24.0 (CH_2), 17.3 (CH*Me*); HRMS *m/z* calcd for $C_{15}H_{22}NO_2$ $(M + H)^+$ 248.1645, found 248.1646 (-2.9 ppm error).

Lab Book PJ-06-22

1-*tert*-Butyl 4-methyl (2*R,4*R**)-2-methylpiperidine-1,4-dicarboxylate *cis*-5b**

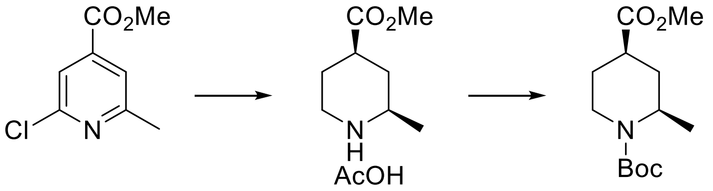

3b *cis*-**4b**·AcOH *cis*-**5b**

PtO_2 (114 mg, 0.5 mmol, 10 mol%) was added to a stirred solution of methyl 2-chloro-6-methylpyridine-4-carboxylate **3b** (928 mg, 5.0 mmol, 1.0 eq.) in glacial acetic acid (20 mL). The reaction flask evacuated under reduced pressure and back filled with Ar three times. After a final evacuation, a balloon of hydrogen was added, and the reaction mixture was stirred vigorously at rt for 16 h. The mixture was filtered through Celite and washed with MeOH (20 mL) and the filtrate was evaporated under reduced pressure to give a >95:5 diastereomeric mixture of crude piperidine *cis*-**4b**·AcOH. *cis*-**4b**·AcOH was dissolved in CH_2Cl_2 (30 mL), Et_3N (3.48 ml, 25.0 mmol, 5.0 eq.) and Boc_2O (1.31 g, 6.0 mmol, 1.2 eq.) were added. The resulting mixture was stirred at rt for 16 h. Then, water (20 mL) and CH_2Cl_2 (20 mL) were added, and the two layers were separated. The aqueous layer was extracted with CH_2Cl_2 (3 × 10 mL). The combined organic layers were dried ($MgSO_4$), filtered and evaporated under reduced pressure to give the crude product. Purification by flash column chromatography on silica with 85:15 hexane-EtOAc as eluent gave piperidine *cis*-**5b** (1.16 g, 90%, >95:5 dr) as a clear oil, R_F (8:2 hexane-EtOAc) 0.24; IR (ATR) 1732 (C=O, Ester), 1686 (C=O, Boc), 1409, 1363, 1198, 1170, 1072, 1051 cm^{-1}; ^1H NMR (400 MHz, $CDCl_3$) δ 4.25–4.08 (m, 1H, NCH), 3.83 (ddd, J = 14.0, 6.0, 2.5 Hz, 1H, NCH), 3.70 (s, 3H, CO_2Me), 3.08 (ddd, J = 14.0, 12.0, 4.0 Hz, 1H, NCH), 2.69–2.51 (m, 1H, CH), 2.04–1.85 (m, 3H, CH), 1.73 (ddd, J = 13.5, 12.0, 6.0 Hz, 1H, CH), 1.45 (s, 9H, C*Me*3), 1.07 (d, J = 7.0 Hz, 3H, CH*Me*); ^{13}C NMR (101 MHz, $CDCl_3$) δ 175.8 (*C*O2Me), 154.9 (*C*O2CMe3), 79.3 (*C*Me3), 51.8 (CO2*Me*), 46.8 (NCH), 35.7 (CH), 35.5 (NCH_2), 31.5 (CH_2), 28.5 (C*Me*3), 25.6 (CH_2), 17.7 (CH*Me*); HRMS *m/z* calcd for $C_{13}H_{23}NO_4$ $(M + Na)^+$ 280.1519, found 280.1519 (0.0 ppm error). Spectroscopic data consistent with those reported in the literature.[3]

Lab Book – JDF_B_140

The relative stereochemistry of *cis*-**5b** was established through comparison to literature data.[3]

1,4-Di-*tert*-butyl (2*R,4*S**)-2-methylpiperidine-1,4-dicarboxylate *trans*-S1 and 1-*tert*-Butyl 4-methyl (2*R**,4*S**)-2-methylpiperidine-1,4-dicarboxylate *trans*-5b**

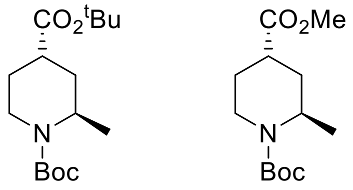

trans-***S*1** *trans*-**5b**

Using general procedure C, *N*-Boc piperidine *cis*-**5b** (100 mg, 0.39 mmol, 1.0 eq.) and KO*t*Bu (470 µL of a 1.0 M solution in THF, 0.47 mmol, 1.2 eq.) in THF (5 mL) gave a 40:60 mixture (by ^{1}H NMR spectroscopy) of *trans*-**S1** and *trans*-**5b** as single diastereomers. Purification by flash column chromatography on silica with 90:10 to 80:20 hexane-EtOAc as eluent gave *tert*-butyl ester *trans*-**S1** (36 mg, 31%) as a clear oil, R_F (9:1 hexane-EtOAc) 0.24; IR (ATR) 1954, 1727 (C=O, ester), 1690 (C=O, Boc), 1412, 1392, 1365, 1335, 1152, 1072 cm^{-1}; ^{1}H NMR (400 MHz, $CDCl_3$) δ 4.46 (br s, 1H, NCH), 4.00 (br d, *J* = 10.5 Hz, 1H, NCH), 2.82 (br t, *J* = 13.0 Hz, 1H, NCH), 2.56–2.40 (m, 1H, CH), 1.85 (br d, *J* = 13.0 Hz, 1H, CH), 1.73–1.68 (m, 2H, CH), 1.52–1.45 (m, 1H, CH), 1.45 (s, 9H, CMe_3), 1.43 (s, 9H, CMe_3), 1.12 (d, *J* = 7.0 Hz, 3H, CH*Me*); ^{13}C NMR (101 MHz, $CDCl_3$) δ 174.5 (*C*O_2CMe_3), 154.7 (CO_2N), 80.3 (*C*Me_3), 79.3 (*C*Me_3), 45.5 (br, NCH_2), 37.6 (br, CH), 37.1 (CH), 32.7 (CH_2), 28.4 (CMe_3), 28.3 (CH_2), 28.0 (CMe_3), 15.9 (CH*Me*); HRMS *m/z* calcd for $C_{16}H_{29}NO_4$ (M + Na)$^+$ 322.1989, found 322.1985 (+1.1 ppm error) and methyl ester *trans*-**5b** (53 mg, 53%) as a clear oil, R_F (9:1 hexane-EtOAc) 0.14; 1958, 1736 (C=O, ester), 1686 (C=O, Boc), 1411, 1365, 1328, 1162, 1125, 1072 cm^{-1}; ^{1}H NMR (400 MHz, $CDCl_3$) δ 4.47 (br s, 1H, NCH), 4.00 (br d, *J* = 10.5 Hz, 1H, NCH), 3.67 (s, 3H, CO_2Me), 2.83 (br t, *J* = 13.0 Hz, 1H, NCH), 2.70–2.51 (m, 1H, CH), 1.88 (d, *J* = 13.0 Hz, 1H, CH), 1.77–1.72 (m, 2H, CH), 1.57–1.46 (m, 1H, CH), 1.44 (s, 9H, CMe_3), 1.12 (d, *J* = 7.0 Hz, 3H, CH*Me*); ^{13}C NMR (101 MHz, $CDCl_3$) δ 175.5 (*C*O_2Me), 154.6 (CO_2N), 79.5 (*C*Me_3), 51.8 (CO_2*Me*), 45.6 (br, NCH), 37.5 (NCH_2), 36.2 (br, CH), 32.7 (CH_2), 28.4 (CMe_3), 28.1 (CH_2), 15.8 (CH*Me*); HRMS *m/z* calcd for $C_{13}H_{23}NO_4$ (M + Na)$^+$ 280.1519, found 280.1521 (–0.5 ppm error).

Lab Book – JDF_B_280

Methyl (3*R,6*S**)-6-methylpiperidine-3-carboxylate *cis*-4c and methyl (3*R**,6*R**)-6-methylpiperidine-3-carboxylate *trans*-4c**

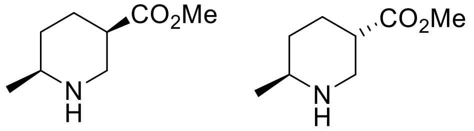

cis-**4c** *trans*-**4c**

Using general procedure B, PtO_2 (60 mg, 0.26 mmol, 10 mol%) and methyl-6-methylnicotinate (400 mg, 2.64 mmol, 1.0 eq.) in AcOH (4 mL) gave the crude product which contained an 85:15 mixture (by ^{1}H NMR spectroscopy) of piperidines *cis*-**4c** and *trans*-**4c** (386 mg, 93%) as a colourless oil, ^{1}H NMR (400 MHz, $CDCl_3$) δ 3.70 (s, 2.55H, OMe), 3.65 (s, 0.45H, OMe), 3.41 (ddd, *J* = 13.0, 2.5, 2.5 Hz, 1H, NCH), 2.81 (dd, *J* = 13.0, 4.0 Hz, 1H, NCH), 2.64 (dqd, *J* = 13, 6.5, 3.0 Hz, 1H, NC*H*Me), 2.48-2.44 (m, 1H, CH), 2.20-2.12 (m, 1H, CH), 2.09 (br, 1H, NH), 1.71-1.61 (m, 1H, CH), 1.54 (ddd, *J* = 13.5, 7.0, 3.5 Hz, 1H, CH), 1.23-1.12 (m, 1H, CH), 1.07-1.02 (m, 3H, Me); ^{13}C NMR (100.6 MHz, $CDCl_3$) δ 175.2 (C=O), 51.8 (OMe), 51.8 (CH), 47.4 (NCH_2), 39.1 (CH), 31.5 (CH_2), 26.1 (CH_2), 22.7 (Me). Spectroscopic data of *cis*-**4c** consistent with those reported in the literature.[4]

Lab Book – PJ-04-56.

The relative stereochemistry of *cis*-**4c** and *trans*-**4c** has been proven unambiguously in our previous work,[2] and through synthesis of the *N*-Cbz analogue and comparison with literature data.[4]

Methyl (3*R,6*S**)-1-benzyl-6-methylpiperidine-3-carboxylate *cis*-5c and methyl (3*R**,6*R**)-1-benzyl-6-methylpiperidine-3-carboxylate *trans*-5c**

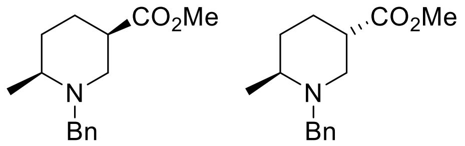

cis-**5c** *trans*-**5c**

BnBr (0.24 mL, 1.99 mmol, 1.2 eq.) was added dropwise to a stirred solution of an 85:15 mixture of piperidines *cis*-**4c** and *trans*-**4c** (260 mg, 1.66 mmol, 1.0 eq.) in saturated $Na_2CO_{3(aq)}$ (2 mL) and CH_2Cl_2 (2 mL) at rt under Ar. The resulting solution was stirred at rt for 16 h. The two layers were separated, and the aqueous layer was extracted with CH_2Cl_2 (3 × 10 mL). The combined organics were

dried ($MgSO_4$) and evaporated under reduced pressure to give the crude product as a colourless oil. Purification by flash column chromatography on silica with 90:10 hexane-EtOAc as eluent gave *N*-benzyl piperidine *cis*-**5c** (325 mg, 79%) as a colourless oil, R_F (90:10 hexane-EtOAc) 0.1; IR (ATR) 2947, 1732 (C=O), 1434, 1192, 1149, 697 cm^{-1}; ^{1}H NMR (400 MHz, $CDCl_3$) δ 7.34-7.27 (m, 4H, Ph), 7.22 (m, 1H, Ph), 3.78 (d, *J* = 13.5 Hz, 1H, NC*H*Ph), 3.62 (s, 3H, OMe), 3.37 (d, *J* = 13.5 Hz, 1H, NC*H*Ph), 2.83 (dd, *J* = 11.5, 7.5 Hz, 1H, NCH), 2.74-2.65 (m, 1H, NC*H*Me), 2.57-2.49 (m, 1H, $CHCO_2$), 2.44 (dd, *J* = 11.5, 4.0 Hz, 1H, NCH), 1.92-1.82 (m, 1H, CH), 1.74-1.63 (m, 2H, CH), 1.57 (m, 1H, CH), 1.06 (d, *J* = 6.5 Hz, 3H, CH*Me*); ^{13}C NMR (100.6 MHz, $CDCl_3$) δ 174.9 (C=O), 139.8 (*ipso*-Ph), 128.8 (Ph), 128.2 (Ph), 126.9 (Ph), 58.7 (NCH$_2$Ph), 53.9 (N*C*HMe), 51.5 (OMe), 50.1 (NCH_2), 41.3 (*C*HCO$_2$), 31.1 (CH_2), 23.4 (CH_2), 14.2 (CH*Me*); HRMS *m/z* calcd for $C_{15}H_{21}NO_2$ (M + H)$^+$ 248.1645, found 248.1636 (+4.1 ppm error).

Lab Book – PJ-06-23

Using general procedure C, *N*-benzyl piperidine *cis*-**5c** (150 mg, 0.61 mmol, 1.0 eq.) and. KO*t*Bu (0.73 mL of a 1 M solution in THF, 0.73 mmol, 1.2 eq.) in THF (5 mL) gave a 75:25 mixture (by ^{1}H NMR spectroscopy) of *N*-benzyl piperidines *trans*-**5c** and *cis*-**5c**. Purification by flash column chromatography on silica with 90:10 hexane-EtOAc as eluent gave an 85:15 mixture of *N*-benzyl piperidine *cis*-**5c** and *trans*-**5c** (43 mg, 28%) as a colourless oil and *N*-benzyl piperidine *trans*-**5c** (80 mg, 53%) as a colourless oil, R_F (80:20 hexane-EtOAc) 0.18; IR (ATR) 2947, 1732 (C=O), 1329, 1144, 697 cm^{-1}; ^{1}H NMR (400 MHz, $CDCl_3$) δ 4.05 (d, *J* = 13.5 Hz, 1H, NC*H*Ph), 3.58 (s, 3H, OMe), 3.16 (d, *J* = 13.5 Hz, 1H, NC*H*Ph), 2.98 (dd, *J* = 11.5, 3.5 Hz, 1H, NCH), 2.49 (dddd, *J* = 11.5, 11.5, 3.5, 3.5 Hz, 1H, $CHCO_2$), 2.28-2.19 (m, 1H, NC*H*Me), 2.04 (dd, *J* = 11.5, 11.5 Hz, 1H, NCH), 1.99-1.92 (m, 1H, CH), 1.74-1.68 (m, 1H, CH), 1.50 1.31 (m, 2H, CH), 1.18 (d, *J* = 6.0 Hz, 3H, CH*Me*); ^{13}C NMR (100.6 MHz, $CDCl_3$) δ 175.0 (C=O), 139.2 (*ipso*-Ph), 129.1 (Ph), 128.3 (Ph), 126.9 (Ph), 57.9 (N*C*H$_2$Ph), 56.4 (NC*H*Me), 54.1 (NCH_2), 51.6 ($CHCO_2$), 42.2 (OMe), 34.0 (CH_2), 27.7 (CH_2), 20.7 (CH*Me*); HRMS *m/z* calcd for $C_{15}H_{21}NO_2$ (M + H)$^+$ 248.1645, found 248.1638 (+2.3 ppm error).

Lab Book – PJ-06-25

Methyl (2*R,6*R**)-6-methylpiperidine-2-carboxylate *cis*-4d**

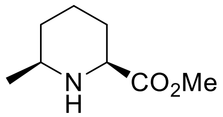

cis-**4d**

Using general procedure B, PtO_2 (30 mg, 0.13 mmol, 10 mol%) and methyl 6-methylpyridine-2-carboxylate (0.18 mL, 1.32 mmol, 1.0 eq.) in AcOH (4.9 mL) gave the crude product which contained (by ^{1}H NMR spectroscopy) only piperidine *cis*-**4d** (200 mg, 97%) as a colourless oil. IR (ATR) 2929, 1737 (C=O), 1436, 1212, 1056, 735 cm^{-1}; ^{1}H NMR (400 MHz, $CDCl_3$) δ 3.68 (s, 3H, OMe), 3.34 (dd, J = 11.0, 3.0 Hz, 1H, NC*H*CO_2), 2.62 (dqd, J = 12.5, 6.5, 2.5 Hz, 1H, NC*H*Me), 1.99-1.92 (m, 1H, CH), 1.88-1.80 (m, 1H, CH), 1.62-1.54 (m, 1H, CH), 1.45-1.26 (m, 2H, CH), 1.08 (d, J = 6.5 Hz, 3H, CH*Me*), 1.06-0.96 (m, 1H, CH); ^{13}C NMR (100.6 MHz, $CDCl_3$) δ 173.8 (C=O), 59.4 (NCH), 52.0 (OMe), 51.9 (N*C*HMe), 33.8 (CH_2), 29.0 (CH_2), 24.6 (CH_2), 22.9 (CH*Me*); HRMS *m/z* calcd for $C_8H_{15}NO_2$ $(M + H)^+$ 158.1176, found 158.1174 (+1.3 ppm error).

Lab Book – PJ-05-88
The relative stereochemistry of *cis*-**4d** has been proven unambiguously in our previous work.[2]

Methyl (2*R,6*R**)-1-benzyl-6-methylpiperidine-2-carboxylate *cis*-5d**

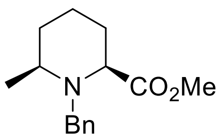

cis-**5d**

BnBr (0.23 mL, 1.91 mmol, 3.0 eq.) was added dropwise to a stirred solution of a >95:5 mixture of piperidines *cis*-**4d** and *trans*-**4d** (100 mg, 0.64 mmol, 1.0 eq.) in saturated $Na_2CO_{3(aq)}$ (2 mL) and CH_2Cl_2 (2 mL) at rt under Ar. The resulting solution was stirred at rt for 16 h. The two layers were separated, and the aqueous layer was extracted with CH_2Cl_2 (3 × 10 mL). The combined organics were dried ($MgSO_4$) and evaporated under reduced pressure to give the crude product as a colourless oil. Purification by flash column chromatography on silica with 80:20 hexane-EtOAc as eluent gave *N*-benzyl piperidine *cis*-**5d** (139 mg, 88%) as a yellow oil, R_F (95:5 hexane-EtOAc) 0.04; IR (ATR) 2932, 1747 (C=O), 1453, 1163, 729, 696 cm^{-1}; ^{1}H NMR (400 MHz, $CDCl_3$) δ 7.36-7.27 (m, 4H, Ph), 7.24-7.19 (m, 1H, Ph), 3.84 (d, J = 15.5 Hz, 1H, NC*H*Ph), 3.73 (d, J = 15.5 Hz, 1H, NC*H*Ph), 3.60 (s, 3H,

OMe), 3.17 (dd, J = 10.5, 3.5 Hz, 1H, $CHCO_2$), 2.41 (dqd, J = 13.0, 6.0, 3.0 Hz, 1H, C*H*Me), 1.85-1.78 (m, 1H, CH), 1.76-1.66 (m, 2H, CH), 1.64-1.56 (m, 1H, CH), 1.46-1.35 (m, 1H, CH), 1.29 (dddd, J = 13.0, 8.0, 2.0, 2.0 Hz, 1H, CH), 1.14 (d, J = 6.0 Hz, 3H, CH*Me*); ^{13}C NMR (100.6 MHz, $CDCl_3$) δ 174.9 (C=O), 138.5 (*ipso*-Ph), 129.2 (Ph), 128.1 (Ph), 126.9 (Ph), 66.0 (N*C*HCO_2), 56.4 (*C*H_2Ph), 55.7 (N*C*HMe), 51.8 (OMe), 34.2 (CH_2), 30.3 (CH_2), 23.2 (CH_2), 21.4 (CH*Me*); HRMS *m/z* calcd for $C_{15}H_{21}NO_2$ $(M + H)^+$ 248.1645, found 248.1648 (–1.5 ppm error).

Lab Book - PJ-05-91

The stereochemistry of *cis*-**5d** was established through analysis of the coupling constants from the ^{1}H NMR spectrum. Proton at C-2 has $^3J_{HH}$ coupling constant of 10.5 Hz and the proton at the C-6 has $^3J_{HH}$ coupling constant of 13.0 Hz which suggests that the protons at the C-2 and C-6 position are axial and therefore confirms the *cis* relative stereochemistry.

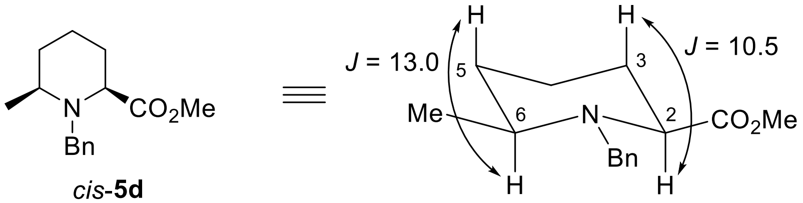

cis-**5d**

Methyl 5-methylpyridine-2-carboxylate 3e

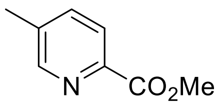

3e

Using general procedure A, thionyl chloride (1.03 mL, 14.2 mmol, 2.0 eq.) and 5-methylpyridine-2-carboxylic acid (973 mg, 7.10 mmol, 1.0 eq.) in MeOH (20 mL) gave pyridine **3e** (771 mg, 72%) as a white solid; mp 56–57 °C (lit.,[5] mp 54–55 °C); IR (ATR) 1725 (C=O), 1318, 1287, 1250, 1124, 779, 702, 644 cm^{-1}; ^{1}H NMR (400 MHz, $CDCl_3$) δ 8.55 (d, J = 1.5 Hz, 1H, Ar), 8.03 (d, J = 8.0 Hz, 1H, Ar), 7.63 (dd, J = 8.0, 1.5 Hz, 1H, Ar), 3.98 (s, 3H, CO_2Me), 2.41 (s, 3H, Ar*Me*); ^{13}C NMR (101 MHz, , $CDCl_3$) δ 165.8 (*C*O_2Me), 150.3 (Ar), 145.3 (*ipso*-Ar), 137.4 (*ipso*-Ar), 137.3 (Ar), 124.8 (Ar), 52.8 (CO_2*Me*), 18.6 (Ar*Me*); HRMS *m/z* calcd for $C_8H_9NO_2$ $(M + H)^+$ 152.0706, found 152.0705 (+0.8 ppm error). Spectroscopic data consistent with those reported in the literature.[5]

Lab Book – JDF_B_170

Methyl (2*R,5*R**)-1-benzyl-5-methylpiperidine-2-carboxylate *cis*-5e and methyl (2*R**,5*S**)-1-benzyl-5-methylpiperidine-2-carboxylate *trans*-5e**

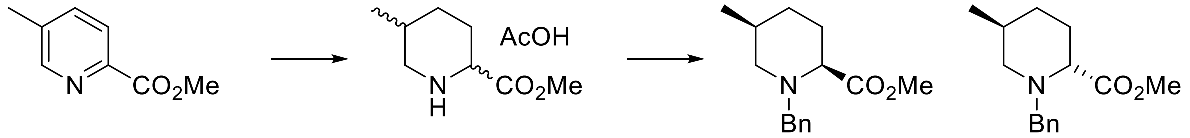

3e **4e**·AcOH *cis*-**5e** *trans*-**5e**

PtO_2 (83 mg, 0.37 mmol, 10 mol%) was added to a stirred solution of ester **3e** (552 mg, 3.65 mmol, 1.0 eq.) in glacial acetic acid (10 mL). The reaction flask evacuated under reduced pressure and back filled with Ar three times. After a final evacuation, a balloon of hydrogen was added, and the reaction mixture was stirred vigorously at rt for 16 h. The mixture was filtered through Celite and washed with MeOH (30 mL), and the filtrate was evaporated under reduced pressure to give a 75:25 mixture of crude diastereomeric piperidines **4e**·AcOH (1.14 g). Crude **4e**·AcOH was dissolved in CH_2Cl_2 (10 mL) and sat. $Na_2CO_{3(aq.)}$ (10 mL) and benzyl bromide (478 μL, 4.02 mmol, 1.1 eq.) were added. The resulting mixture was stirred at rt for 16 h. Then, water (30 mL) and CH_2Cl_2 (30 mL) were added, and the two layers were separated. The aqueous layer was extracted with CH_2Cl_2 (3 × 20 mL). The combined organic layers were dried ($MgSO_4$), filtered and evaporated under reduced pressure to give a 75:25 mixture of crude products. Purification by flash column chromatography on silica with 9:1 to 8:2 hexane-Et_2O as eluent gave piperidine *cis*-**5e** (631 mg, 70%) as a clear oil, R_F (8:2 hexane-Et_2O) 0.5; IR (ATR) 2927, 1735 (C=O), 1453, 1193, 1154, 1136, 1004, 735, 698 cm^{-1}; ^1H NMR (400 MHz, $CDCl_3$) δ 7.34–7.20 (m, 5H, Ph), 3.81 (d, J = 14.0 Hz, 1H, CH_2Ph), 3.75 (d, J = 14.0 Hz, 1H, CH_2Ph), 3.69 (s, 3H, OMe), 3.49 (dd, J = 5.5, 3.0 Hz, 1H, NCH), 2.64 (dd, J = 11.0, 11.0 Hz, 1H, NCH), 2.54 (dd, J = 11.0, 4.0 Hz, 1H, NCH), 2.00 (ddd, J = 13.0, 7.0, 3.5 Hz, 1H, CH), 1.83 (dddd, J = 13.0, 13.0, 5.5, 3.5 Hz, 1H, CH), 1.72–1.52 (m, 3H, CH), 1.12–0.98 (m, 1H, CH), 0.84 (d, J = 6.5 Hz, 3H, CH*Me*); ^{13}C NMR (101 MHz, $CDCl_3$) δ 174.0 (CO$_2$Me), 139.5 (*ipso*-Ph), 128.5 (Ar), 128.2 (Ar), 126.8 (Ar), 60.0 (NCH), 59.7 (CH$_2$Ph), 54.8 (NCH_2), 50.9 (CO$_2$*Me*), 30.8 (CH), 29.2 (CH_2), 28.2 (CH_2), 19.3 (CH*Me*); HRMS *m/z* calcd for $C_{15}H_{21}NO_2$ $(M + H)^+$ 248.1645, found 248.1637 (–3.1 ppm error) and piperidine *trans*-**5e** (203 mg, 22%) as a clear oil, R_F (8:2 hexane-Et_2O) 0.25; IR (ATR) 1747 (C=O), 1734, 1274, 1192, 1162, 1112, 738, 698 cm^{-1}; ^1H NMR (400 MHz, $CDCl_3$) δ 7.35–7.23 (m, 5H, Ph), 3.79–3.75 (m,

4H, CO_2Me + CH_2Ph), 3.22 (d, J = 13.0 Hz, 1H, CH_2Ph), 2.91 (dd, J = 11.0, 3.0 Hz, 1H, NCH), 2.82 (ddd, J = 11.0, 3.0, 1.0 Hz, 1H), 1.91 (ddd, J = 12.5, 6.5, 3.0 Hz, 1H, CH), 1.82–1.60 (m, 3H, CH), 1.56 (dd, J = 11.0, 11.0 Hz, 1H, NCH), 0.98–0.84 (m, 1H, CH), 0.79 (d, J = 6.5 Hz, 1H, CH*Me*); ^{13}C NMR (101 MHz, $CDCl_3$) δ 174.8 (CO_2Me), 137.2 (*ipso*-Ph), 129.5 (Ph), 128.1 (Ph), 127.1 (Ph), 66.2 (NCH), 60.8 (CH_2Ph), 59.1 (NCH_2), 51.9 (CO_2*Me*), 32.0 (CH_2), 30.3 (CH), 29.9 (CH_2), 19.4 (CH*Me*); HRMS *m/z* calcd for $C_{15}H_{21}NO_2$ $(M + H)^+$ 248.1645, found 248.1643 (–0.8 ppm error).

Lab Book – JDF_B_170 and JDF_B_174

The stereochemistry of *trans*-**5e** was established through analysis of the coupling constants from the ^{1}H NMR spectrum. Proton at C-2 has $^3J_{HH}$ coupling constants of 11.0 and 3.0 Hz and the proton at the C-5 has $^3J_{HH}$ coupling constant of 11.0 and 3.0 Hz which suggests that the protons at the C-2 and C-5 position are axial and therefore confirms the *trans* relative stereochemistry.

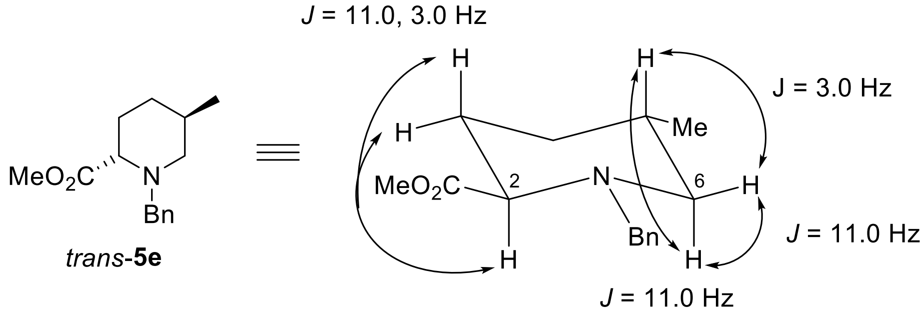

Using general procedure C, *N*-benzyl piperidine *cis*-**5e** (50 mg, 0.20 mmol, 1.0 eq.) and KO*t*Bu (0.24 mL of a 1 M solution in THF, 0.24 mmol, 1.2 eq.) in THF (3 mL) gave a 50:50 mixture (by ^{1}H NMR spectroscopy) of *N*-benzyl piperidines *trans*-**5e** and *cis*-**5e**. Purification by flash column chromatography on silica with 90:10 hexane-Et_2O as eluent gave *N*-benzyl piperidine *cis*-**5e** (23 mg, 48%) as a colourless oil and *N*-benzyl piperidine *trans*-**5e** (20 mg, 40%) as a colourless oil.

Lab Book – PJ-05-32.

Methyl (2*R,3*S**)-3-methylpiperidine-2-carboxylate *cis*-4f and methyl (2*R**,3*R**)-3-methylpiperidine-2-carboxylate *trans*-4f**

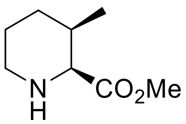

cis-**4f**

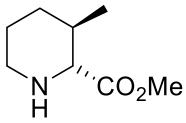

trans-**4f**

Using general procedure B, PtO_2 (20 mg, 0.10 mmol, 10 mol%) and methyl-3-methylpicolinate (0.13 mL, 0.99 mmol, 1.0 eq.) in AcOH (1.5 mL) gave the crude product which contained an 85:15 mixture (by ^{1}H NMR spectroscopy) of piperidines *cis*-**4f** and *trans*-**4f** (150 mg, 97%) as a colourless oil, IR (ATR) 2929, 1741 (C=O), 1435, 1202 1005, 758 cm^{-1}; ^{1}H NMR (400 MHz, $CDCl_3$) δ 3.72 (s, 0.45H, OMe), 3.71 (s, 2.55H, OMe), 3.52 (d, J = 3.5 Hz, 0.85H, $NCHCO_2$), 3.16-3.08 (m, 1H, NCH), 2.96 (d, J = 10.0 Hz, 0.15H, $NCHCO_2$), 2.65-2.57 (m, 1H, NCH), 2.24-2.13 (m, 1H, C*H*Me), 1.68-1.57 (m, 3H, CH), 1.39-1.32 (m, 1H, CH), 0.93 (d, J = 7.0 Hz, 2.55H, CH*Me*), 0.87 (d, J = 7.0 Hz, 0.45H, CH*Me*); ^{13}C NMR (100.6 MHz, $CDCl_3$) for *cis*-**4f**: δ 173.6 (C=O), 62.2 (N*C*HCO_2), 51.8 (OMe), 46.1 (NCH_2), 30.9 (CH_2), 30.6 (*C*HMe), 21.2 (CH_2), 13.6 (CH*Me*); for *trans*-**4f**: 66.5 (N*C*HCO_2), 45.9 (CH_2), 34.9 (CH), 33.1 (CH_2), 26.7 (CH_2), 19.0 (CH*Me*); HRMS *m*/*z* calcd for $C_8H_{15}NO_2$ $(M + H)^+$ 158.1176, found 158.1177 (–1.3 ppm error).

Lab Book – PJ-04-37

The relative stereochemistry of *cis*-**4f** and *trans*-**4f** has been proven unambiguously in our previous work.[2]

Methyl (2*R,3*S**)-1-benzyl-3-methylpiperidine-2-carboxylate *cis*-5f and methyl (2*R**,3R*)-1-benzyl-3-methylpiperidine-2-carboxylate *trans*-5f**

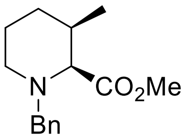

cis-**5f**

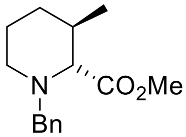

trans-**5f**

PhCHO (0.36 mL, 3.5 mmol, 1.1 eq.) was added dropwise to a stirred solution of an 85:15 mixture of piperidines *cis*-**4f** and *trans*-**4f** (500 mg, 3.18 mmol, 1.0 eq.), $NaBH(OAc)_3$ (1.35 g, 6.37 mmol, 2.0 eq.) and AcOH (0.03 mL, 0.64 mmol, 0.2 eq.) in DCE (30 mL) at rt under Ar. The resulting mixture was

stirred at rt for 18 h. The reaction mixture was poured into sat $NaHCO_{3(aq)}$ (100 mL) and extracted with CH_2Cl_2 (3 × 50 mL). The combined organics were washed with brine (100 mL), dried ($MgSO_4$) and evaporated under reduced pressure to give the crude product as a green oil which contained a 90:10 mixture (by ^{1}H NMR spectroscopy) of piperidines *cis*-**5f** and *trans*-**5f**. Purification by flash column chromatography on silica with 98:2 hexane-EtOAc as eluent gave *N*-benzyl piperidine *cis*-**5f** (626 mg, 80%) as a yellow oil, R_F (95:5 hexane-EtOAc) 0.23; IR (ATR) 2928, 1728 (C=O), 1453, 1148, 697 cm^{-1}; ^{1}H NMR (400 MHz, $CDCl_3$) δ 7.32-7.29 (m, 4H, Ph), 7.25-7.22 (m, 1H, Ph), 3.69 (s, 3H, OMe), 3.61 (d, *J* = 13.5 Hz, 1H, NC*H*Ph), 3.56 (d, *J* = 13.5 Hz, 1H, NC*H*Ph), 3.45 (d, *J* = 5.0 Hz, 1H, $NCHCO_2$), 3.00-2.92 (m, 1H, NCH), 2.54-2.45 (m, 1H, NCH), 2.03-1.93 (m, 1H, C*H*Me), 1.71-1.64 (m, 1H, CH), 1.60-1.46 (m, 3H, CH), 0.90 (d, *J* = 7.0 Hz, 3H, CH*Me*); ^{13}C NMR (100.6 MHz, $CDCl_3$) δ 173.0 (C=O), 139.1 (*ipso*-Ph), 128.9 (Ph), 128.3 (Ph), 127.1 (Ph), 66.2 (N*C*HCO$_2$), 60.2 (N*C*H$_2$Ph), 50.5 (OMe), 47.0 (NCH_2), 33.2 (*C*HMe), 27.8 (CH_2), 25.2 (CH_2), 18.2 (CH*Me*); HRMS *m/z* calcd for $C_{15}H_{21}NO_2$ (M + H)$^+$ 248.1645, found 248.1643 (–0.7 ppm error) and piperidine *trans*-**5f** (82 mg, 10%) as a yellow oil, R_F (95:5 hexane-EtOAc) 0.1; IR (ATR) 2928, 1727 (C=O), 1453, 1147, 734, 697 cm^{-1}; ^{1}H NMR (400 MHz, $CDCl_3$) δ 7.32-7.29 (m, 4H, Ph), 7.26-7.22 (m, 1H, Ph), 3.76 (s, 3H, OMe), 3.71 (d, *J* = 13.5 Hz, 1H, NC*H*Ph), 3.25 (d, *J* = 13.5 Hz, 1H, NC*H*Ph), 2.87 (ddd, *J* = 11.0, 5.5, 5.5 Hz, 1H, NCH), 2.64 (d, *J* = 9.0 Hz, 1H, $NCHCO_2$), 1.95-1.86 (m, 2H, CH), 1.76-1.68 (m, 1H, CH), 1.61-1.53 (m, 2H, CH), 1.05-0.94 (m, 1H, CH), 0.90 (d, *J* = 7.0 Hz, 3H, CH*Me*); ^{13}C NMR (100.6 MHz, $CDCl_3$) δ 174.3 (C=O), 137.7 (*ipso*-Ph), 129.6 (Ph), 128.3 (Ph), 127.2 (Ph), 73.7 (N*C*HCO$_2$), 61.2 (NCH_2Ph), 51.8 (OMe), 51.3 (NCH_2), 34.4 (*C*HMe), 32.0 (CH_2), 24.6 (CH_2), 18.9 (CH*Me*); MS (ESI) *m/z* 248 (M + H)$^+$; HRMS *m/z* calcd for $C_{15}H_{21}NO_2$ (M + H)$^+$ 248.1645, found 248.1641 (+1.3 ppm error). Spectroscopic data consistent with those reported in the literature.[6]

Lab Book – PJ-02-45.

Using general procedure C, *N*-benzyl piperidine *cis*-**5f** (173 mg, 0.70 mmol, 1.0 eq.) and KO*t*Bu (0.84 mL of a 1 M solution in THF, 0.84 mmol, 1.2 eq.) in THF (5 mL) gave a 70:30 mixture (by ^{1}H NMR spectroscopy) of *N*-benzyl piperidines *trans*-**5f** and *cis*-**5f**. Purification by flash column chromatography on silica with 95:5 hexane-EtOAc as eluent gave *N*-benzyl piperidine *cis*-**5f** (40 mg, 23%) as a colourless oil and *N*-benzyl piperidine *trans*-**5f** (104 mg, 60%) as a colourless oil.

Lab Book – PJ-06-08.

Methyl 4-methylpyridine-2-carboxylate 3g

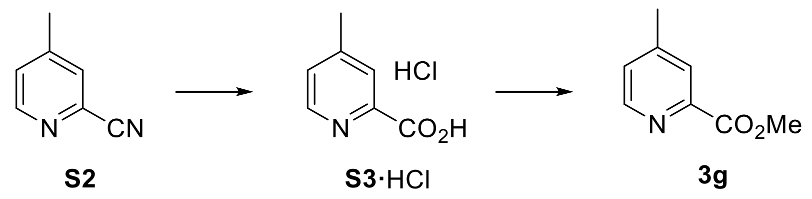

4-Methylpyridine-2-carbonitrile **S2** (1.18 g, 10.0 mmol, 1.0 eq.) was suspended in 6M $HCl_{(aq)}$ (10 mL) and the resulting solution was stirred and heated at reflux for 16 h. After cooling to rt, the solvent was evaporated under reduced pressure. Then, MeCN (20 mL) was added to the residue and the resulting white solid was collected by filtration, washed with MeCN (30 mL) and dried under reduced pressure to give crude carboxylic acid **S3**·HCl (1.84 g) as a white solid containing ~5% **S2**. Using general procedure A, thionyl chloride (1.45 mL, 20.0 mmol, 2.0 eq.) and crude **S3**·HCl (1.84 g, 10.0 mmol max., 1.0 eq.) in MeOH (20 mL) gave pyridine **3g** (980 mg, 65%) as a clear oil; ^{1}H NMR (400 MHz, $CDCl_3$) δ 8.57 (d, J = 5.0 Hz, 1H, Ar), 7.96 (s, 1H, Ar), 7.28 (d, J = 5.0 Hz, 1H, Ar), 3.98 (s, 3H, CO_2Me), 2.42 (s, 3H, Ar*Me*); ^{13}C NMR (101 MHz, $CDCl_3$) δ 165.9 (CO_2Me), 149.5 (Ar), 148.5 (*ipso*-Ar), 147.7 (*ipso*-Ar), 127.7 (Ar), 126.0 (Ar), 52.82 (CO_2*Me*), 21.0 (Ar*Me*); HRMS *m/z* calcd for $C_8H_9NO_2$ $(M + H)^+$ 152.0706, found 152.0707 (–0.9 ppm error). Spectroscopic data consistent with those reported in the literature.[7]

Lab Book – JDF_B_143

1-*tert*-Butyl 2-methyl (2*R,4*S**)-4-methylpiperidine-1,2-dicarboxylate *cis*-5g**

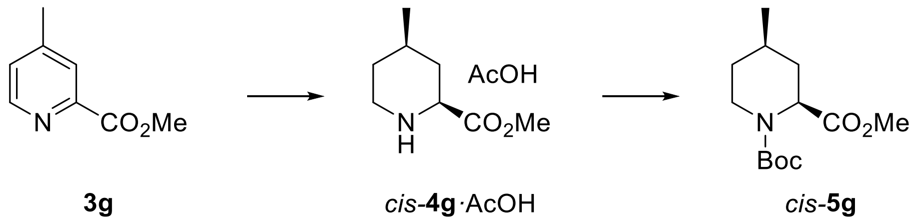

PtO_2 (68 mg, 0.2 mmol, 10 mol%) was added to a stirred solution of ester **3g** (453 mg, 3.0 mmol, 1.0 eq.) in glacial acetic acid (10 mL). The reaction flask evacuated under reduced pressure and back filled with Ar three times. After a final evacuation, a balloon of hydrogen was added, and the reaction

mixture was stirred vigorously at rt for 16 h. The mixture was filtered through Celite and washed with MeOH (20 mL) and the filtrate was evaporated under reduced pressure to give crude piperidine *cis*-**4g**·AcOH as a single diastereomer. *cis*-**4g**·AcOH was dissolved in CH_2Cl_2 (20 mL), Et_3N (2.09 ml, 15.0 mmol, 5.0 eq.) and Boc_2O (785 mg, 3.6 mmol, 1.2 eq.) were added. The resulting mixture was stirred at rt for 16 h. Then, water (20 mL) and CH_2Cl_2 (20 mL) were added, and the two layers were separated. The aqueous layer was extracted with CH_2Cl_2 (3 × 10 mL). The combined organic layers were dried ($MgSO_4$), filtered and evaporated under reduced pressure to give the crude product. Purification by flash column chromatography on silica with 9:1 hexane-EtOAc as eluent gave piperidine *cis*-**5g** (657 mg, 85%) as a clear oil, R_F (9:1 hexane-EtOAc) 0.17; IR (ATR) 1745 (C=O, ester), 1695 (C=O, Boc), 1393, 1366, 1198, 1154, 1130, 1070 cm^{-1}; ^{1}H NMR (400 MHz, $CDCl_3$) δ 4.33 (br t, *J* = 6.0 Hz, 1H, NCH), 3.72 (s, 3H, CO_2*Me*), 3.53 (br s, 1H, NCH), 3.43–3.34 (br m, 1H, NCH), 2.01–1.92 (m, 1H, CH), 1.89–1.68 (m, 3H, CH), 1.44 (s, 9H, CMe_3), 1.37–1.27 (m, 1H, CH), 0.92 (d, *J* = 6.5 Hz, 3H, CH*Me*); ^{13}C NMR (101 MHz, $CDCl_3$) δ 173.4 (*C*O_2Me), 80.1 (*C*Me_3), 54.2 (NCH), 52.0 (CO_2*Me*), 39.0 (NCH_2), 33.2 (CH_2), 30.9 (CH2), 28.3 (C*Me*$_3$), 26.1 (CH), 19.0 (CH*Me*); HRMS *m/z* calcd for $C_{13}H_{23}NO_4$ $(M + Na)^+$ 280.1519, found 280.1520 (–0.1 ppm error). Spectroscopic data consistent with those reported in the literature.[8]

Lab Book – JDF_B_146

The relative stereochemistry of *cis*-**5g** was established through comparison to literature data.[8]

1-*tert*-Butyl 2-methyl (2*R,4*R**)-4-methylpiperidine-1,2-dicarboxylate *trans*-5g**

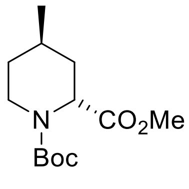

trans-**5g**

n-BuLi (155 µL of a 2.5 M solution in hexanes, 0.39 mmol, 2.0 eq.) was added dropwise to a stirred solution of diisopropylamine (55 µL, 0.39 mmol, 2.0 eq.) in THF (30 mL) at –78 °C under N_2. The resulting solution was stirred at –78 °C for 15 min, then warmed to 0 °C and stirred at 0 °C for 30 min. The solution was cooled to –78 °C and a solution of ester *cis*-**5g** (50 mg, 0.19 mmol, 1.0 eq.) in THF (2 mL) was added dropwise. The resulting solution was stirred at –78 °C for 2 h, then sat. $NH_4Cl_{(aq)}$ (5 mL) was added, and the resulting mixture warmed to rt. Water (5 mL) and EtOAc (10 mL) were added

and the two layers were separated. The aqueous layer was extracted with EtOAc (3 × 10 mL). The combined organic layers were dried ($MgSO_4$), filtered and evaporated under reduced pressure to give a 80:20 mixture of *trans*-**5g** and *cis*-**5g**. Purification by flash column chromatography on silica with 9:1 hexane-EtOAc as eluent gave piperidine *trans*-**5g** (33 mg, 65%) as a clear oil, R_F (9:1 hexane-EtOAc) 0.24; IR (ATR) 2929, 2873, 1972, 1745 (C=O, Ester), 1695 (C=O, Boc), 1393, 1365, 1198, 1178, 1158, 1142 cm^{-1}; ^1H NMR (400 MHz, $CDCl_3$) (50:50 mixture of rotamers) δ 4.92 (d, *J* = 5.0 Hz, 0.5H, NCH), 4.73 (d, *J* = 5.0 Hz, 0.5H, NCH), 4.03 (dd, *J* = 13.5, 3.0 Hz, 0.5H, NCH), 3.92 (dd, *J* = 13.5, 3.0 Hz, 0.5H, NCH), 3.72 (s, 1.5H, CO_2Me), 3.71 (s, 1.5H, CO_2Me), 2.98 (td, *J* = 13.5, 3.0 Hz, 0.5H, NCH), 2.87 (td, *J* = 13.5, 3.0 Hz, 0.5H, NCH), 2.20–2.10 (m, 1H, CH), 1.66–1.53 (m, 1H, CH), 1.46 (s, 4.5H, C*Me*$_3$), 1.42 (s, 4.5H, C*Me*$_3$), 1.40–1.24 (m, 2H, CH), 1.14–0.98 (m, 1H, CH), 0.91 (d, *J* = 6.0 Hz, 3H, CH*Me*); ^{13}C NMR (101 MHz, $CDCl_3$) (mixture of rotamers) δ 172.7 (*C*O_2Me), 172.4 (*C*O_2Me), 155.9 (*C*O_2CMe$_3$), 155.5 (*C*O_2CMe$_3$), 80.0 (*C*Me$_3$), 55.0 (NCH), 53.9 (NCH), 52.0 (CO_2*Me*), 41.9 (NCH_2), 41.1 (NCH_2), 34.9 (CH_2), 34.8 (CH_2), 33.4 (CH_2), 33.1 (CH_2), 28.4 (C*Me*$_3$), 28.3 (C*Me*$_3$), 27.5 (CH), 27.3 (CH), 21.9 (CH*Me*); HRMS m/z calcd for $C_{13}H_{23}NO_4$ $(M + Na)^+$ 280.1519, found 280.1522 (–0.8 ppm error). Spectroscopic data consistent with those reported in the literature.[9]

Lab Book – JDF_B_157

Methyl 3-methylpyridine-4-carboxylate 3h

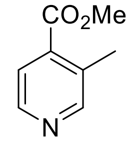

3h

Using general procedure A, thionyl chloride (0.23 mL, 3.16 mmol, 1.1 eq.) and 3-methyl-isonicotinic acid (393 mg, 2.87 mmol, 1.0 eq.) in MeOH (20 mL) gave pyridine **3h** (345 mg, 80%) as an orange oil, ^1H NMR (400 MHz, MeOH-*d$_4$*) δ 8.66-8.51 (m, 2H, Ar), 7.77 (d, *J* = 5.0 Hz, 1H, Ar), 3.94 (s, 3H, OMe), 2.56 (s, 3H, Me); ^{13}C NMR (100.6 MHz, MeOH-*d$_4$*) δ 167.6 (C=O), 153.2 (Ar), 148.3 (Ar), 138.9 (*ipso*-Ar), 135.3 (*ipso*-Ar), 124.7 (Ar), 53.0 (OMe), 18.1 (Me). Spectroscopic data consistent with those reported in the literature.[10]

Lab Book – PJ-04-79.

Methyl (3*R,4*R**)-3-methylpiperidine-4-carboxylate *cis*-4h and methyl (3*R**,4*S**)-3-methylpiperidine-4-carboxylate *trans*-4h**

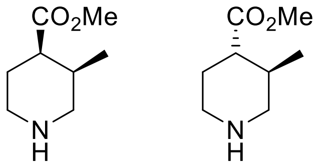

cis-**4h** *trans*-**4h**

Using general procedure B, PtO_2 (24 mg, 0.11 mmol, 10 mol%) and pyridine **3h** (163 mg, 1.1 mmol, 1.0 eq.) in AcOH (2 mL) gave the crude product which contained an 85:15 mixture (by ^{1}H NMR spectroscopy) of piperidines *cis*-**4h** and *trans*-**4h** (147 mg, 89%) as a colourless oil, IR (ATR) 3319 (NH), 2951, 1726 (C=O), 1434, 1269, 1171 cm^{-1}; ^{1}H NMR (400 MHz, $CDCl_3$) δ 3.66 (s, 3H, OMe), 3.07 (ddd, *J* = 12.5, 4.5, 4.5 Hz, 1H, NCH), 2.89-2.76 (m, 2H, NCH, $CHCO_2$), 2.64-2.56 (m, 2H, NCH), 2.26-2.19 (m, 0.15H, C*H*Me), 2.14-2.06 (m, 0.85H, C*H*Me), 1.79 (dddd, *J* = 14.5, 10.5, 10.5, 4.0 Hz, 1H, CH), 1.65-1.58 (m, 1H, CH), 1.53 (br s, 1H, NH), 0.94 (d, *J* = 7.0 Hz, 2.55H, CH*Me*), 0.82 (d, *J* = 7.5 Hz, 0.45H, CH*Me*); ^{13}C NMR (100.6 MHz, $CDCl_3$) δ 175.1 (C=O), 52.0 (NCH_2), 51.5 (OMe), 45.4 (NCH_2), 44.7 (*C*HCO_2), 31.3 (*C*HMe), 24.5 (CH_2), 13.8 (CH*Me*); HRMS *m/z* calcd for $C_8H_{15}NO_2$ $(M + H)^+$ 158.1176, found 158.1178 (–1.3 ppm error).
Lab Book- PJ-05-09

The relative stereochemistry of *cis*-**4h** and *trans*-**4h** has been proven unambiguously in our previous work.[2]

1-*tert*-Butyl 4-methyl (3*R,4*R**)-3-methylpiperidine-1,4-dicarboxylate *cis*-5h and 1-*tert*-butyl 4-methyl (3*R**,4*S**)-3-methylpiperidine-1,4-dicarboxylate *trans*-5h**

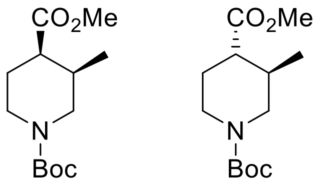

cis-**5h** *trans*-**5h**

Et_3N (0.16 mL, 1.16 mmol, 2.0 eq.) was added dropwise to a stirred solution of an 85:15 mixture of piperidines *cis*-**4h** and *trans*-**4h** (91 mg, 0.58 mmol, 1.0 eq.) and Boc_2O (252 mg, 1.16 mmol, 2.0 eq.)

in CH_2Cl_2 (5 mL) at rt under Ar. The resulting solution was stirred at rt for 16 h. Then, the solvent was evaporated under reduced pressure to give the crude product as a green oil. Purification by flash column chromatography on silica with 99:1 CH_2Cl_2-acetone as eluent gave an 65:35 mixture (by ^1H NMR spectroscopy) of Boc piperidines *cis*-**5h** and *trans*-**5h** (20 mg, 14%) as a colourless oil and Boc piperidine *cis*-**5h** (73 mg, 49%) as a colourless oil, R_F (99:1 CH_2Cl_2-acetone) 0.14; IR (ATR) 2970, 1733 (C=O, CO_2Me), 1687 (C=O, Boc), 1425, 1161, 880 cm^{-1}; ^1H NMR (400 MHz, $CDCl_3$) (60:40 mixture of rotamers) δ 4.12-3.97 (m, 0.6H, NCH), 3.97-3.83 (m, 0.4H, NCH), 3.81-3.72 (m, 1H, NCH), 3.67 (s, 1.8H, OMe), 3.67 (s, 1.2H, OMe), 3.09-3.01 (m, 1H, NCH), 3.00-2.88 (m, 0.4H, NCH), 2.88-2.77 (m, 0.6H, NCH), 2.59 (ddd, *J* = 10.5, 6.5, 3.0 Hz, 1H, $CHCO_2$), 2.18 (br m, 1H, C*H*Me), 1.89-1.76 (m, 1H, CH), 1.75-1.59 (m, 1H, CH), 1.44 (s, 5.4H, CMe_3), 1.43 (s, 3.6H, CMe_3), 0.88 (d, *J* = 7.0 Hz, 1.8H, CH*Me*), 0.87 (d, *J* = 7.0 Hz, 1.2H, CH*Me*); ^{13}C NMR (100.6 MHz, $CDCl_3$) (rotamers) δ 174.4 (C=O, CO_2Me), 155.3 (C=O, Boc), 79.6 (*C*Me_3), 51.7 (OMe), 49.6 (NCH_2), 48.3 (NCH_2), 44.7 ($CHCO_2$), 43.0 (NCH_2), 42.3 (NCH_2), 31.3 (*C*HMe), 28.5 (C*Me*$_3$), 23.4 (CH_2), 22.9 (CH_2), 13.2 (CH*Me*); HRMS *m/z* calcd for $C_{13}H_{23}NO_4$ $(M + Na)^+$ 280.1519, found 280.1517 (+0.6 ppm error).

Lab Book – PJ-05-21

Et_3N (0.16 mL, 1.16 mmol, 2.0 eq.) was added dropwise to a stirred solution of an 85:15 mixture of piperidines *cis*-**5h** and *trans*-**5h** (91 mg, 0.58 mmol, 1.0 eq.) and Boc_2O (253 mg, 1.16 mmol, 2.0 eq.) in CH_2Cl_2 (5 mL) at rt under Ar. The reaction mixture was stirred at rt for 16 h. Then, the solvent was evaporated under reduced pressure to give the crude product as a green oil. Purification by flash column chromatography on silica with 90:10 hexane-Et_2O as eluent gave a 90:10 mixture (by ^1H NMR spectroscopy) of *N*-Boc piperidines *cis*-**5h** and *trans*-**5h** (117 mg, 78%) as a colourless oil.

Lab Book – PJ-04-100

Using general procedure C, a 90:10 mixture of *N*-Boc piperidines *cis*-**5h** and *trans*-**5h** (50 mg, 0.19 mmol, 1.0 eq.) and KO*t*Bu (0.23 mL of a 1 M solution in THF, 0.23 mmol, 1.2 eq.) in THF (5 mL) gave a 90:10 mixture (by ^1H NMR spectroscopy) of Boc piperidine *trans*-**5h** and *cis*-**5h** (45 mg, 0.17 mmol, 90%) as a colourless oil, R_F (90:10 hexane-EtOAc) 0.14; IR (ATR) 2931, 1735 (C=O, CO_2Me), 1689 (C=O, Boc), 1420, 1158, 767 cm^{-1}; ^1H NMR (400 MHz, $CDCl_3$) for *trans*-**5h**; δ 4.17-3.92 (m, 2H, NCH), 3.68 (s, 3H, OMe), 2.73-2.63 (m, 1H, NCH), 2.41-2.24 (m, 1H, NCH), 2.08 (ddd, *J* = 12.0,

11.0, 4.0 Hz, 1H, $CHCO_2$), 1.87-1.75 (m, 2H, CH and C*H*Me), 1.69-1.57 (dddd, *J* = 12.5, 12.5, 12.5, 4.5 Hz, 1H, CH), 1.45 (s, 9H, CMe_3), 0.87 (d, *J* = 6.5 Hz, 3H, CH*Me*); ^{13}C NMR (100.6 MHz, $CDCl_3$) for *trans*-**5h**; δ 175.3 (C=O, CO_2Me), 154.7 (C=O, Boc), 79.8 (*C*Me_3), 51.8 (OMe), 50.0 (NCH_2), 49.5 (*C*HCO_2), 43.2 (NCH_2), 33.1 (C*H*Me), 28.8 (CH_2), 28.6 (C*Me₃*), 17.1 (CH*Me*); HRMS *m/z* calcd for $C_{13}H_{23}NO_4$ (M + Na)$^+$ 280.1519, found 280.1523 (–1.7 ppm error).

Lab Book – PJ-05-17

Methyl (3*R,4*R**)-4-methylpiperidine-3-carboxylate *cis*-4i and methyl (3*R**,4*S**)-4-methylpiperidine-3-carboxylate *trans*-4i**

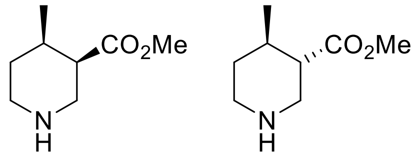

cis-**4i** *trans*-**4i**

Using general procedure B, PtO_2 (45 mg, 0.20 mmol, 30 mol%) and methyl-4-methylnicotinate (100 mg, 0.66 mmol, 1.0 eq.) in AcOH (1 mL) gave the crude product which contained a 65:35 mixture (by ^{1}H NMR spectroscopy) of piperidines *cis*-**4i** and *trans*-**4i** (93 mg, 90%) as a yellow oil, IR (ATR) 3314 (NH), 2952, 1726 (C=O), 1435, 1197, 1139, 754 cm^{-1}; ^{1}H NMR (400 MHz, $CDCl_3$) δ 3.66 (s, 3H, OMe), 3.20-3.10 (m, 1H, NCH), 3.07-2.96 (m, 1H, NCH), 2.86-2.79 (m, 0.65H, NCH), 2.69-2.57 (m, 1.65H, CH), 2.55-2.50 (m, 0.65H, CH), 2.15 (br m, 1H, NH), 2.12-2.04 (m, 0.35H, CH), 2.04-1.93 (m, 0.65H, CH), 1.83-1.72 (m, 0.35H, CH), 1.66 (dddd, *J* = 7.0, 7.0, 5.0, 2.0, Hz, 0.35H, CH), 1.59-1.46 (m, 1H, CH), 1.16-1.03 (m, 0.35H, CH), 0.94 (d, *J* = 7.0 Hz, 1.95H, CH*Me*), 0.90 (d, *J* = 6.5 Hz, 1.05H, CH*Me*); ^{13}C NMR (100.6 MHz, $CDCl_3$) δ 174.9 (C=O), 174.7 (C=O), 51.6 (OMe), 51.3 (OMe), 49.1 (NCH_2), 46.6 (NCH_2), 46.3 (NCH_2), 45.4 (NCH_2), 44.7 (*C*HCO_2), 34.4 (CH_2), 33.4 (*C*HMe), 31.5 (*C*HMe), 31.2 (CH_2), 20.6 (CH*Me*), 18.2 (CH*Me*); HRMS *m/z* calcd for $C_8H_{16}NO_2$ (M + H)$^+$ 158.11776, found 158.1173 (+1.9 ppm error).

Lab Book – PJ-04-43

1-*tert*-Butyl 3-methyl 4-methylpiperidine-1,3-dicarboxylate *cis*-5i and *trans*-5i

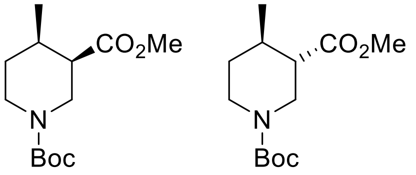

cis-**5i** *trans*-**5i**

Et_3N (0.14 mL, 0.98 mmol, 2.0 eq.) was added dropwise to a stirred solution of a 65:35 mixture of piperidines *cis*-**4i** and *trans*-**4i** (77 mg, 0.49 mmol, 1.0 eq.) and Boc_2O (214 mg, 0.98 mmol, 2.0 eq.) in CH_2Cl_2 (5 mL) at rt under Ar. The reaction mixture was stirred at rt for 16 h. Then, the solvent was evaporated under reduced pressure to give the crude product as an orange oil. Purification by flash column chromatography on silica with 9:1 hexane-EtOAc as eluent gave Boc piperidine *trans*-**5i** (28 mg, 22%) as a colourless oil, a 65:35 mixture of Boc piperidines *cis*-**5i** and *trans*-**5i** (22 mg, 18%) and a 90:10 mixture of Boc piperidines *cis*-**5i** and *trans*-**5i** (67 mg, 54%). The 65:35 and 90:10 mixtures of Boc piperidines *cis*-**5i** and *trans*-**5i** were combined and purified by flash column chromatography on silica with 98:2 CH_2Cl_2-acetone as eluent to give a 65:35 mixture (by ^1H NMR spectroscopy) of Boc piperidines *cis*-**5i** and *trans*-**5i** (18 mg, 14%) as a colourless oil and Boc piperidine *cis*-**5i** (59 mg, 47%) as a colourless oil, R_F (99:1 CH_2Cl_2-acetone) 0.18; IR (ATR) 2971, 1734 (C=O, CO_2Me), 1689 (C=O, Boc), 1422, 1365, 1163, 1138, 866 cm^{-1}; ^1H NMR (400 MHz, $CDCl_3$) δ 3.67 (br s, 3H, OMe), 3.61-3.55 (m, 2H, NCH), 3.47-3.40 (m, 1H, NCH), 3.40-3.32 (m, 1H, NCH), 2.60-2.53 (m, 1H, $CHCO_2$), 2.19-2.10 (m, 1H, C*H*Me), 1.72-1.64 (m, 1H, CH), 1.61-1.54 (m, 1H, CH), 1.45 (s, 9H, CMe_3), 0.97 (d, J = 7.0 Hz, 3H, CH*Me*); ^{13}C NMR (100.6 MHz, $CDCl_3$) δ 173.2 (C=O, CO_2Me), 154.5 (C=O, Boc), 79.6 (*C*Me_3), 51.6 (OMe), 44.7 (*C*HCO_2), 42.9 (CH_2), 40.5 (CH_2), 30.5 (*C*HMe), 30.3 (CH_2), 28.5 (C*Me*$_3$), 15.6 (CH*Me*); HRMS *m/z* calcd for $C_{13}H_{23}NO_4$ $(M + Na)^+$ 280.1519, found 280.1523 (–1.5 ppm error).

Lab Book – PJ-05-15

Et_3N (0.94 mL, 6.78 mmol, 2.0 eq.) was added dropwise to a stirred solution of a 65:35 mixture of piperidines *cis*-**4i** and *trans*-**4i** (533 mg, 3.39 mmol, 1.0 eq.) and Boc_2O (1.5 g, 6.78 mmol, 2.0 eq.) in CH_2Cl_2 (10 mL) at rt under Ar. The reaction mixture was stirred at rt for 16 h. Then, the solvent was evaporated under reduced pressure to give the crude product as an orange oil. Purification by flash

column chromatography on silica with 9:1 hexane-EtOAc as eluent gave a 65:35 mixture of Boc piperidines *cis*-**5i** and *trans*-**5i** (740 mg, 84%) as a colourless oil.

Lab Book – PJ-04-71.

Using general procedure C, a 65:35 mixture of piperidines *cis*-**5i** and *trans*-**5i** (150 mg, 0.58 mmol, 1.0 eq.) and KO*t*Bu (0.47 mL of a 1 M solution in THF, 0.47 mmol, 1.2 eq.) in THF (9 mL) gave the crude product as an orange oil. Purification by flash column chromatography on silica with 80:20 hexane-EtOAc as eluent gave Boc piperidine *trans*-**5i** (68 mg, 68%) as a colourless oil, R_F (90:10 hexane-Et_2O) 0.19; IR (ATR) 2930, 1733 (C=O, CO_2Me), 1692 (C=O, Boc), 1419, 1241, 1145, 963 cm^{-1}; ^1H NMR (400 MHz, $CDCl_3$) δ 4.32-3.98 (m, 2H, NCH), 3.68 (s, 3H, OMe), 2.86-2.61 (m, 2H, NCH and $CHCO_2$), 2.14-2.05 (m, 1H, NCH), 1.86-1.73 (m, 1H, C*H*Me), 1.71-1.58 (m, 1H, CH), 1.44 (s, 9H, CMe_3), 1.20-1.05 (m, 1H, CH), 0.92 (d, *J* = 6.5 Hz, 3H, CH*Me*); ^{13}C NMR (100.6 MHz, $CDCl_3$) δ 174.1 (C=O, CO_2Me), 154.6 (C=O, Boc), 79.9 (*C*Me_3), 51.7 (OMe), 49.8 (*C*HCO_2), 46.2 (NCH_2), 44.0 (CH_2), 33.7 (*CH*Me), 33.0 (CH_2), 28.5 (C*Me₃*), 20.1 (CH*Me*); HRMS *m/z* calcd for $C_{13}H_{23}NNaO_4$ (M + Na)$^+$ 280.1519, found 280.1519 (+0.3 ppm error).

Lab Book – PJ-05-26

The relative stereochemistry of *cis*-**5i** and *trans*-**5i** has been proven unambiguously in our previous work.[2]

Methyl 3-methylpyridine-5-carboxylate 3j

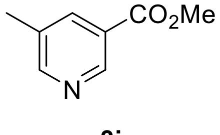

3j

Using general procedure A, thionyl chloride (60 μL, 0.80 mmol, 1.1 eq.) and methyl 3-methylpyridine-5-carboxylate (100 mg, 0.73 mmol, 1.0 eq.) in MeOH (5 mL) gave pyridine **3j** (106 mg, 96%) as a white solid, mp 37-39 °C; IR (ATR) 2960, 1712 (C=O), 1574, 1293, 1108, 766 cm^{-1}; ^1H NMR (400 MHz, $CDCl_3$) δ 9.03 (d, *J* = 2.0 Hz, 1H, Ar), 8.61 (d, *J* = 2.0 Hz, 1H, Ar), 8.13-8.09 (m, 1H,

Ar), 3.95 (s, 3H, OMe), 2.40 (s, 3H, Me); ^{13}C NMR (100.6 MHz, MeOH-*d*$_4$) δ 166.9 (C=O), 154.3 (Ar), 148.2 (Ar), 139.1 (Ar), 135.7 (Ar), 127.5 (Ar), 52.9 (OMe), 18.2 (Me); HRMS *m/z* calcd for $C_8H_9NO_2$ (M + H)$^+$ 152.0706, found 152.0702 (+2.7 ppm error). Spectroscopic data consistent with those reported in the literature.[11]

Lab Book – PJ-04-81

Methyl (3*R,5*S**)-5-methylpiperidine-3-carboxylate *cis*-4j and methyl (3*S**,5*S**)-5-methylpiperidine-3-carboxylate *trans*-4j**

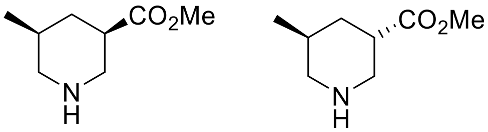

cis-**4j** *trans*-**4j**

Using general procedure B, PtO_2 (15 mg, 0.066 mmol, 10 mol%) and pyridine **3j** (100 mg, 0.66 mmol, 1.0 eq.) in AcOH (5 mL), gave the crude product which contained a 60:40 mixture (by ^{1}H NMR spectroscopy) of piperidines *trans*-**4j** and *cis*-**4j** (96 mg, 93%) as a colourless oil, IR (ATR) 2951, 1725 (C=O), 1435, 1198, 1177, 860 cm^{-1}; ^{1}H NMR (400 MHz, $CDCl_3$) δ 3.69 (s, 1.8H, OMe), 3.65 (s, 1.2H, OMe), 3.30-3.23 (m, 1H, NCH), 2.97-2.90 (m, 1H, NCH), 2.73 (dd, *J* = 13.0, 3.5 Hz, 0.6H, NCH), 2.59-2.54 (m, 1H, $CHCO_2$), 2.51-2.43 (m, 0.4H, NCH), 2.24 (dd, *J* = 13.0, 10.0 Hz, 0.6H, NCH), 2.15-2.04 (m, 1.2H, NCH, CH), 1.69-1.57 (m, 0.6H, C*H*Me), 1.57-1.47 (m, 0.4H, C*H*Me), 1.39-1.30 (m, 0.6H, NCH), 1.17 (m, 0.4H, CH), 0.86 (d, *J* = 6.6 Hz, 1.2H, CH*Me*), 0.84 (d, *J* = 6.7 Hz, 1.8H, CH*Me*); ^{13}C NMR (100.6 MHz, $CDCl_3$) δ 175.4 (C=O), 174.7 (C=O), 53.8 (NCH_2), 53.7 (NCH_2), 51.8 (OMe), 51.7 (OMe), 48.3 (CH_2), 47.4 (CH_2), 43.4 (*C*HCO_2), 39.7 (*C*HCO_2), 36.2 (CH_2), 34.5 (CH_2), 31.8 (*C*HMe), 28.8 (*C*HMe), 19.5 (CH*Me*), 19.1 (CH*Me*); HRMS *m/z* calcd for $C_8H_{15}NO_2$ (M + H)$^+$ 158.1176, found 158.1171 (–1.5 ppm error).

Lab Book – PJ-05-28

Using general procedure B, 10% Pd/C (70 mg, 0.07 mmol, 10 mol%) and pyridine **3j** (100 mg, 0.66 mmol, 1.0 eq.) in AcOH (5 mL), gave the crude product which contained a 70:30 mixture (by ^{1}H NMR spectroscopy) of piperidines *trans*-**4j** and *cis*-**4j** (94 mg, 91%) as a colourless oil.

Lab Book – PJ-05-25

The relative stereochemistry of *cis*-**4j** and *trans*-**4j** has been proven unambiguously in our previous work.[2]

Methyl (3*R,5*S**)-1-benzyl-5-methylpiperidine-3-carboxylate *cis*-5j and methyl (3*R**,5*R**)-1-benzyl-5-methylpiperidine-3-carboxylate *trans*-5j**

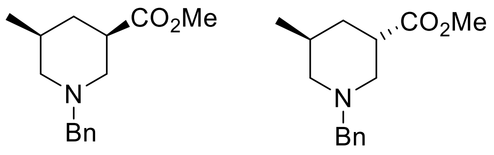

cis-**5j** *trans*-**5j**

BnBr (0.09 mL, 0.76 mmol, 1.2 eq.) was added dropwise to a 70:30 solution of piperidine *trans*-**4j** and *cis*-**4j** (100 mg, 0.63 mmol, 1.0 eq.) and Et_3N (0.11 mL, 0.79 mmol, 1.2 eq.) in CH_2Cl_2 (4 mL) at rt under Ar. The resulting solution was stirred at rt for 16 h. The two layers were separated, and the aqueous layer was extracted with CH_2Cl_2 (4 × 10 mL). The combined organics were dried ($MgSO_4$) and evaporated under reduced pressure to give the crude product as a colourless oil. Purification by flash column chromatography on silica with 90:10 hexane-EtOAc as eluent gave *N*-benzyl piperidine *trans*-**5j** (77 mg, 51%) as a colourless oil, R_F (90:10 hexane-EtOAc) 0.14; IR (ATR) 2949, 1732 (C=O), 1453, 1199, 1150, 697 cm^{-1}; ^{1}H NMR (400 MHz,$CDCl_3$) δ 7.31-7.28 (m, 4H, Ph), 7.25-7.22 (m, 1H, Ph), 3.67 (s, 3H, OMe), 3.56 (d, *J* = 13.5 Hz, 1H, NC*H*Ph), 3.39 (d, *J* = 13.5 Hz, 1H, NC*H*Ph), 2.96-2.88 (m, 1H, NCH), 2.69-2.63 (m, 1H, $CHCO_2$), 2.63-2.57 (m, 1H, NCH), 2.31-2.23 (m, 1H, NCH), 2.06-1.94 (m, 2H, C*H*Me and CH), 1.91-1.83 (m, 1H, NCH), 1.24-1.14 (m, 1H, CH), 0.92 (d, *J* = 6.5 Hz, 3H, CH*Me*); ^{13}C NMR (100.6 MHz, $CDCl_3$) δ 174.9 (C=O), 138.8 (*ipso*-Ph), 128.9 (Ph), 128.2 (Ph), 127.0 (Ph), 63.1 (CH_2Ph), 61.3 (NCH_2), 54.9 (NCH_2), 51.6 (OMe), 39.7 ($CHCO_2$), 33.4 (CH_2), 28.1 (*C*HMe), 19.2 (CH*Me*); HRMS *m*/*z* calcd for $C_{15}H_{21}NO_2$ $(M + H)^+$ 248.1645, found 248.1646 (–0.9 ppm error) and *N*-benzyl piperidine *cis*-**5j** (27 mg, 17%) as a colourless oil, R_F (90:10 hexane-EtOAc) 0.05; IR (ATR) 2951, 1732 (C=O), 1434, 1136, 1154, 697 cm^{-1}; ^{1}H NMR (400 MHz, $CDCl_3$) δ 7.34-7.29 (m, 4H, Ph), 7.280-7.24 (m, 1H, Ph), 3.64 (s, 3H, OMe), 3.52 (s, 2H, NCH_2Ph), 3.12-3.06 (m, 1H, NCH), 2.84-2.79 (m, 1H, NCH), 2.64 (dddd, *J* = 12.0, 12.0, 4.0, 4.0 Hz, 1H, $CHCO_2$), 2.04-

1.98 (m, 1H, CH), 1.95 (dd, *J* = 12.0, 12.0 Hz, 1H, NCH), 1.78-1.66 (m, 1H, C*H*Me), 1.55 (d, *J* = 12.0 Hz, 1H, NCH), 1.02 (ddd, *J* = 12.0, 12.0, 12.0 Hz, 1H, CH), 0.87 (d, *J* = 6.5 Hz, 3H, CH*Me*); ^{13}C NMR (100.6 MHz, $CDCl_3$) δ 174.9 (C=O), 140.1 (*ipso*-Ph), 129.2 (Ph), 128.4 (Ph), 127.1 (Ph), 63.2 (*C*H_2Ph), 61.2 (NCH_2), 55.1 (NCH_2), 51.7 (OMe), 42.3 (*C*HCO_2), 35.9 (CH_2), 30.6 (*C*HMe), 19.5 (CH*Me*); HRMS *m/z* calcd for $C_{15}H_{21}NO_2$ $(M + H)^+$ 248.1645, found 248.1647 (–1.1 ppm error).

Lab Book – PJ-06-36

Using general procedure C, piperidine *trans*-**5j** (150 mg, 0.61 mmol, 1.0 eq.) and KO*t*Bu (0.73 mL of a 1 M solution in THF, 0.73 mmol, 1.2 eq.) in THF (5 mL) gave a crude 85:15 mixture (by ^{1}H NMR spectroscopy) of *N*-benzyl piperidines *cis*-**5j** and *trans*-**5j** (149 mg). Purification by flash column chromatography on silica with 90:10 hexane-EtOAc as eluent gave *N*-benzyl piperidine *cis*-**5j** (93 mg, 62%) as a yellow oil.

Lab Book – PJ-06-16

The stereochemistry of *cis*-**5j** was established through analysis of the coupling constants from the ^{1}H NMR spectrum. Proton at C-4 has $^2J_{HH}$ geminal coupling constants of 12.0 Hz and $^3J_{HH}$ coupling constants of 12.0 Hz and 12.0 Hz which shows that the protons at the C-3 and C-5 position are axial and therefore confirms the *cis* relative stereochemistry.

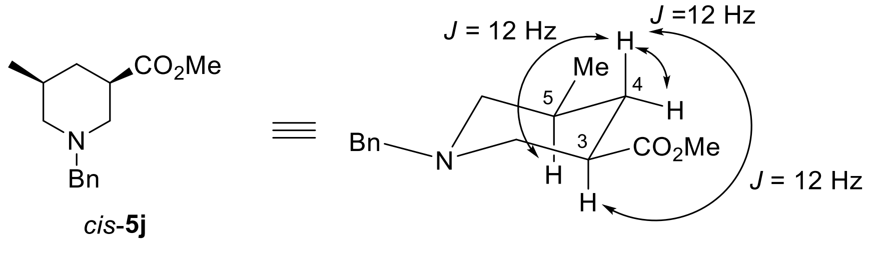

***tert*-Butyl 2-methylpiperidine-1-carboxylate 7a**

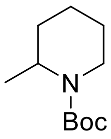

7a

2-Methyl piperidine (5.0 mL, 42.5 mmol, 1.0 eq.) was added dropwise to a stirred solution of di-*tert*-butyl dicarbonate (10.2 g, 46.8 mmol, 1.1 eq.) in CH_2Cl_2 (200 mL) at 0 °C under Ar. The resulting solution was allowed to warm to rt and stirred at rt for 16 h. H_2O (20 mL) was added, and the mixture was extracted with CH_2Cl_2 (3 × 15 mL). The combined organic extracts were dried ($MgSO_4$) and evaporated under reduced pressure to give the crude product. Purification by flash chromatography on silica with 90:10 petrol-Et_2O as eluent gave *N*-Boc methyl piperidine **7a** (7.6 g, 82%) as a colourless oil, R_F (90:10 petrol-Et_2O) 0.3; ^{1}H NMR (400 MHz, $CDCl_3$) δ 4.41-4.30 (m, 1H, NCH), 3.90 (dd, *J* = 13.5, 3.0 Hz, 1H, NCH), 2.79 (ddd, *J* = 13.5, 13.5, 2.5 Hz, 1H, NCH), 1.68-1.45 (m, 5H CH), 1.44 (s, 9H, CMe_3), 1.40-1.32 (m, 1H, CH), 1.10 (d, *J* = 7.0 Hz, 3H, Me); ^{13}C NMR (101.6 MHz, $CDCl_3$) δ 155.0 (C=O), 79.0 (*C*Me_3), 46.0 (NCH), 38.6 (NCH_2), 30.1 (CH_2), 28.5 (C*Me*$_3$), 25.7 (CH_2), 18.7 (CH_2), 15.7 (Me). Spectroscopic data consistent with those reported in the literature.[12]

Lab Book – MCW/2/2

(2*R,6*S**)-1-(*tert*-Butoxycarbonyl)-6-methylpiperidine-2-carboxylic acid *trans*-8d**

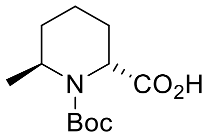

trans-**8d**

s-BuLi (1.0 mL of a 1.3 M solution in hexane, 1.3 mmol, 1.3 eq.) was added dropwise to a stirred solution of *N*-Boc piperidine **7a** (199 mg, 1.0 mmol, 1.0 eq.) and TMEDA (0.19 mL, 1.3 mmol, 1.3 eq.) in Et_2O (7 mL) at –40 °C under Ar. The resulting yellow solution was stirred at –40 °C for 1.5 h. Then, CO_2 (excess) was bubbled through the reaction mixture at –40 °C for 10 min. H_2O (10 mL) was added. 1 M $HCl_{(aq)}$ (15 mL) was added, and the mixture extracted with Et_2O (3 × 10 mL). The combined organic extracts were dried ($MgSO_4$) and evaporated under reduced pressure to give the crude product. Purification by flash chromatography on silica with 90:10 CH_2Cl_2-Et_2O and 0.5% acetic acid and then

70:30 CH_2Cl_2-Et_2O and 0.5% acetic acid as eluent gave piperidine carboxylic acid *trans*-**8d** (199 mg, 82%) as a white solid, mp 82-84 °C; R_F (90:10 CH_2Cl_2-Et_2O) 0.2; IR (ATR) 2977, 2944, 1698 (C=O), 1445, 1380, 1365, 1227, 868, 779, 512 cm^{-1}; ^1H NMR (400 MHz, $CDCl_3$) δ 4.24-4.18 (m, 1H, NCH), 4.18 (dd, *J* = 7.0, 5.0 Hz, 1H, NCH), 2.09-1.80 (m, 3H, CH), 1.69-1.49 (m, 3H, CH), 1.45 (s, 9H, CMe_3), 1.18 (d, *J* = 7.0 Hz, 3H, Me); ^{13}C NMR (101.6 MHz, $CDCl_3$) δ 178.3 (C=O, CO_2Me), 156.4 (C=O, Boc), 80.9 (*C*Me_3), 53.8 (NCH), 47.6 (NCH), 28.3 (C*Me_3*), 27.7 (CH_2), 25.6 (CH_2), 18.8 (Me), 15.0 (CH_2); HMRS (ESI) *m/z* calcd for $C_{12}H_{21}NO_4$ $(M + Na)^+$ 266.1363, found 266.1355 (+2.9 ppm error).

Lab Book Reference: MCW/3/57/2

The relative stereochemistry of *cis*-**8d** has been proven unambiguously in our previous work.[2]

(2*R,6*S**)-1-*tert*-Butyl 2-methyl 6-methylpiperidine-1,2-dicarboxylate *trans*-5d**

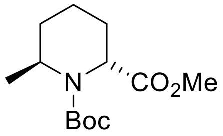

trans-**5d**

Potassium carbonate (2.56 g, 18.5 mmol, 3.0 eq.) was added to a stirred solution of piperidine carboxylic acid *trans*-**8d** (1.50 g, 61.7 mmol, 1.0 eq.) in dimethylformamide (20 mL) at rt under Ar. The resulting suspension was stirred at rt for 30 min before the addition of methyl iodide (1.15 mL, 18.5 mmol, 3.0 eq.). The mixture was then stirred at rt for 18 h. The solvent was evaporated under reduced pressure and H_2O (10 mL) and EtOAc (10 mL) were added. The mixture was extracted with EtOAc (3 × 20 mL). The combined organic extracts were washed with 10% $Na_2S_2O_{3(aq)}$ (15 mL) and the mixture extracted with EtOAc (20 mL). The combined organic extracts were dried ($MgSO_4$) and evaporated under reduced pressure to give the crude product. Purification by flash chromatography on silica with 80:20 hexane-Et_2O as eluent gave piperidine methyl ester *trans*-**5d** (1.55 g, 97%) as a yellow oil, R_F (80:20 hexane-Et_2O) 0.2; IR (ATR) 2972, 2950, 1746 (C=O, CO_2Me), 1692 (C=O, Boc), 1390, 1364, 1163, 1112, 735 cm^{-1}; ^1H NMR (400 MHz, $CDCl_3$) δ 4.26-4.17 (m, 1H, NCH), 4.12 (dd, *J* = 7.0, 5.0 Hz, 1H, NCH), 3.72 (s, 3H, OMe), 2.04-1.92 (m, 1H, CH), 1.91-1.78 (m, 2H, CH), 1.67-1.48 (m, 3H, CH), 1.43 (s, 9H, CMe_3), 1.16 (d, *J* = 7.0 Hz, 3H, Me); ^{13}C NMR (101.6 MHz, $CDCl_3$) δ 173.8 (C=O, CO_2Me), 156.0 (C=O, Boc), 80.1 (*C*Me_3), 54.1 (NCH), 52.0 (OMe), 47.5 (NCH), 28.3 (C*Me_3*),

28.0 (CH_2), 26.2 (CH_2), 18.8 (Me), 15.5 (CH_2); MS (ESI) *m/z* 280 [$(M + Na)^+$, 100], 258 [$(M + H)^+$, 15]; HMRS (ESI) *m/z* calcd for $C_{13}H_{23}NO_4$ $(M + Na)^+$ 280.1519, found 280.1507 (+4.2 ppm error).

Lab Book Reference: mcw/3/59/1

***tert*-Butyl 3-methylpiperidine-1-carboxylate 7b**

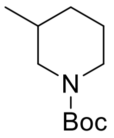

7b

A solution of Boc_2O (1.3 g, 6.0 mmol, 1.2 eq.) in CH_2Cl_2 (5 mL) was added dropwise to a stirred solution of piperidine (0.59 mL, 5.0 mmol, 1.0 eq.), and Et_3N (2.1 mL, 15.0 mmol, 3.0 eq.) in CH_2Cl_2 (15 mL) at rt under Ar. The resulting solution was stirred at rt for 16 h. Then, the reaction mixture was evaporated under reduced pressure to give the crude product as a colourless oil. Purification by flash column chromatography on silica with 99:1 hexane-EtOAc as eluent gave *N*-Boc piperidine **7b** (975 mg, 98%) as a colourless oil, R_F (99:1 hexane-EtOAc) 0.1; IR (ATR) 2959, 1690 (C=O), 1416, 1149, 1075, 969 cm^{-1}; ^1H NMR (400 MHz, $CDCl_3$) δ 4.07-3.74 (m, 2H, NCH), 2.68 (ddd, *J* = 13.0, 11.5, 3.0 Hz, 1H, NCH), 2.49-2.21 (m, 1H, CH), 1.80-1.71 (m, 1H, CH), 1.68-1.50 (m, 2H, NCH and C*H*Me), 1.45 (s, 9H, CMe_3), 1.43-1.35 (m, 1H, CH), 1.09-0.97 (m, 1H, CH), 0.86 (d, *J* = 6.5 Hz, 3H, CH*Me*); ^{13}C NMR (100.6 MHz, $CDCl_3$) δ 155.1 (C=O), 79.3 (*C*Me_3), 51.5 (NCH_2), 50.8 (NCH_2), 44.5 (NCH_2), 43.8 (NCH_2), 33.1 (CH_2), 31.1 (*C*HMe), 28.6 (C*Me₃*), 25.3 (CH_2), 19.1 (CH*Me*); HRMS *m/z* calcd for $C_{11}H_{21}NO_2$ $(M + Na)^+$ 222.1464, found 222.1471 (–3.8 ppm error).

Lab Book - PJ-05-79

(2*R,5*S**)-1-[(*tert*-Butoxy)carbonyl]-5-methylpiperidine-2-carboxylic acid *trans*-8e and (2*R**,5*R**)-1-[(*tert*-butoxy)carbonyl]-5-methylpiperidine-2-carboxylic acid *cis*-8e**

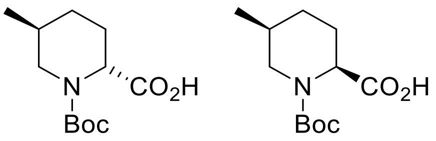

trans-**8e** *cis*-**8e**

s-BuLi (1.0 mL of a 1.3 M solution in hexane, 1.3 mmol, 1.3 eq.) was added drop wise to a stirred solution of *N*-Boc piperidine **7b** (199 mg, 1.0 mmol, 1.0 eq.) and TMEDA (0.19 mL, 1.3 mmol, 1.3 eq.) in Et_2O (4 mL) at –60 °C under Ar. The resulting solution was stirred at –60 °C for 3 h. Then, CO_2 (excess) was bubbled through the reaction mixture at –60 °C for 10 min. 1 M $HCl_{(aq)}$ (10 mL) was added, and the mixture extracted with EtOAc (3 × 10 mL). The combined organic extracts were dried ($MgSO_4$) and evaporated under reduced pressure to give the crude product. Purification by flash chromatography on silica with 99:1 to 98:2 CH_2Cl_2-acetic acid as eluent gave a 90:10 mixture of piperidine carboxylic acid *trans*-**8e** and *cis*-**8e** (140 mg, 57 %) as a white solid, m.p 99-101 °C; R_F (95:5 CH_2Cl_2-AcOH) 0.22; IR (ATR) 2936, 1725 (C=O, CO_2H), 1622 (C=O, Boc), 1438, 1365, 1151, 850 cm^{-1}; ^1H NMR (400 MHz, $CDCl_3$) *trans*-**8e**: δ 11.56-10.82 (m, 1H, CO_2H), 4.97-4.51 (m, 1H, $NCHCO_2$), 3.69-3.45 (m, 1H, NCH), 3.18 (dd, *J* = 13.0, 3.0 Hz, 1H, NCH), 1.97-1.93 (m, 2H, CH_2), 1.92-1.82 (m, 1H, C*H*Me), 1.68-1.54 (m, 1H, CH), 1.53-1.34 (m, 10H, CMe_3 and CH), 0.97 (d, *J* = 7.0 Hz, 2.7H, CH*Me*); ^{13}C NMR (100.6 MHz, $CDCl_3$) δ 178.0 (C=O, CO_2H), 156.5 (C=O, Boc), 80.5 (*C*Me_3), 53.9 (N*C*HCO_2), 47.5 (NCH_2), 28.4 (C*Me_3*), 27.1 (*C*HMe), 27.0 (CH_2), 21.6 (CH_2), 16.7 (CH*Me*); HRMS *m/z* calcd for $C_{12}H_{21}NO_4$ $(M + Na)^+$ 266.1363, found 266.1367 (–3.8 ppm error). Spectroscopic data consistent with those reported in the literature.[13]

Lab Book - PJ-05-90

The relative stereochemistry of *trans*-**8e** was established through comparison to literature data.[13]

1-*tert*-Butyl 2-methyl (2*R,5*S**)-5-methylpiperidine-1,2-dicarboxylate dicarboxylate *trans*-S4 and 1-*tert*-Butyl 2-methyl (2*R**,5*R**)-5-methylpiperidine-1,2-dicarboxylate *cis*-S4**

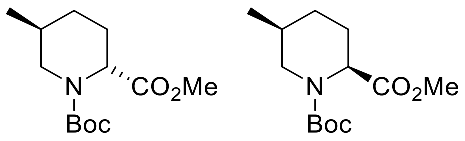

trans-**S4** *cis*-**S4**

Potassium carbonate (86 mg, 0.62 mmol, 3.0 eq.) was added to a stirred solution of piperidine carboxylic acid *trans*-**8e** (50 mg, 0.21 mmol, 1.0 eq.) in dimethylformamide (3 mL) at rt. The resulting suspension was stirred at rt for 30 min before the addition of methyl iodide (0.038 mL, 0.62 mmol, 3.0 eq.). The mixture was then stirred at rt for 18 h. The reaction mixture was taken up into water (3 mL) and extracted with EtOAc (4 × 10 mL). The combined organic extracts were washed with brine (4 × 5 mL), dried ($MgSO_4$) and evaporated under reduced pressure to give the crude product. Purification by flash chromatography on silica with 90:10 hexane-EtOH as eluent gave a 90:10 mixture of piperidine ester *trans*-**S4** and *cis*-**S4** (47 mg, 87%) as a yellow oil, R_F (90:10, hexane-EtOH) 0.17; IR (ATR) 2940, 1743 (C=O, CO_2Me), 1690 (C=O, Boc), 1363, 1147, 864 cm^{-1}; ^{1}H NMR (400 MHz, $CDCl_3$) δ 4.86-4.59 (m, 1H, $NCHCO_2$), 3.72 (s, 3H, OMe), 3.64-3.55 (m, 1H, NCH), 3.20-3.11 (m, 1H, NCH), 1.97-1.90 (m, 2H, CH), 1.90-1.83 (m, 1H, C*H*Me), 1.59-1.50 (m, 1H, CH), 1.44 (s, 9H, CMe_3), 1.41-1.32 (m, 1H), 0.98 (d, *J* = 7.0 Hz, 3H, CH*Me*); ^{13}C NMR (100.6 MHz, $CDCl_3$) δ 172.7 (C=O, CO_2Me), 156.3 (C=O, Boc), 80.1 (*C*Me_3), 54.5 (N*C*HCO_2), 52.2 (OMe), 47.3 (NCH_2), 28.4 (C*Me*$_3$), 27.2 (*C*HMe), 27.1 (CH_2), 21.8 (CH_2), 16.7 (CH*Me*); HRMS *m/z* calcd for $C_{13}H_{23}NNaO_4$ $(M + Na)^+$ 280.1519, found 280.1525 (–2.0 ppm error).

Lab Book – PJ-05-95

Methyl (2*R,5*R**)-1-benzyl-5-methylpiperidine-2-carboxylate *cis*-5e and methyl (2*R**,5*S**)-1-benzyl-5-methylpiperidine-2-carboxylate *trans*-5e**

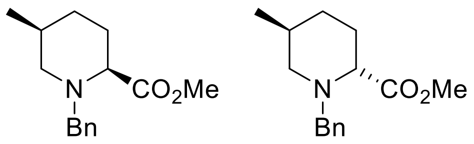

cis-**5e** *trans*-**5e**

TFA (1 mL) was added to a stirred solution of a 90:10 mixture of piperidine ester *trans*-**S4** and *cis*-**S4** (47 mg, 0.18 mmol, 1.0 eq.) in CH_2Cl_2 (5 mL) at 0 °C under Ar. The resulting solution was warmed to rt and stirred for 3 h at rt, then evaporated under reduced pressure. The residue was dissolved in CH_2Cl_2 (2 mL) and sat $Na_2CO_{3(aq)}$ (2 mL) and benzyl bromide (0.06 mL, 0.54 mmol, 3.0 eq.) were added. The resulting mixture was stirred at rt for 16 h. The two layers were separated, and the aqueous layer was extracted with CH_2Cl_2 (3 × 5 mL). The combined organics were dried ($MgSO_4$) and evaporated under reduced pressure to give the crude product as a colourless oil. Purification by flash column chromatography on silica with 90:10 hexane-EtOAc as eluent gave *N*-benzyl piperidine *cis*-**5e** (4 mg, 7%) as a colourless oil and *N*-benzyl piperidine *trans*-**5e** (38 mg, 85%) as a colourless oil.

Lab Book – PJ-05-98.

2. Isomers of dimethyl pyridine 1 and dimethyl NH piperidine 2

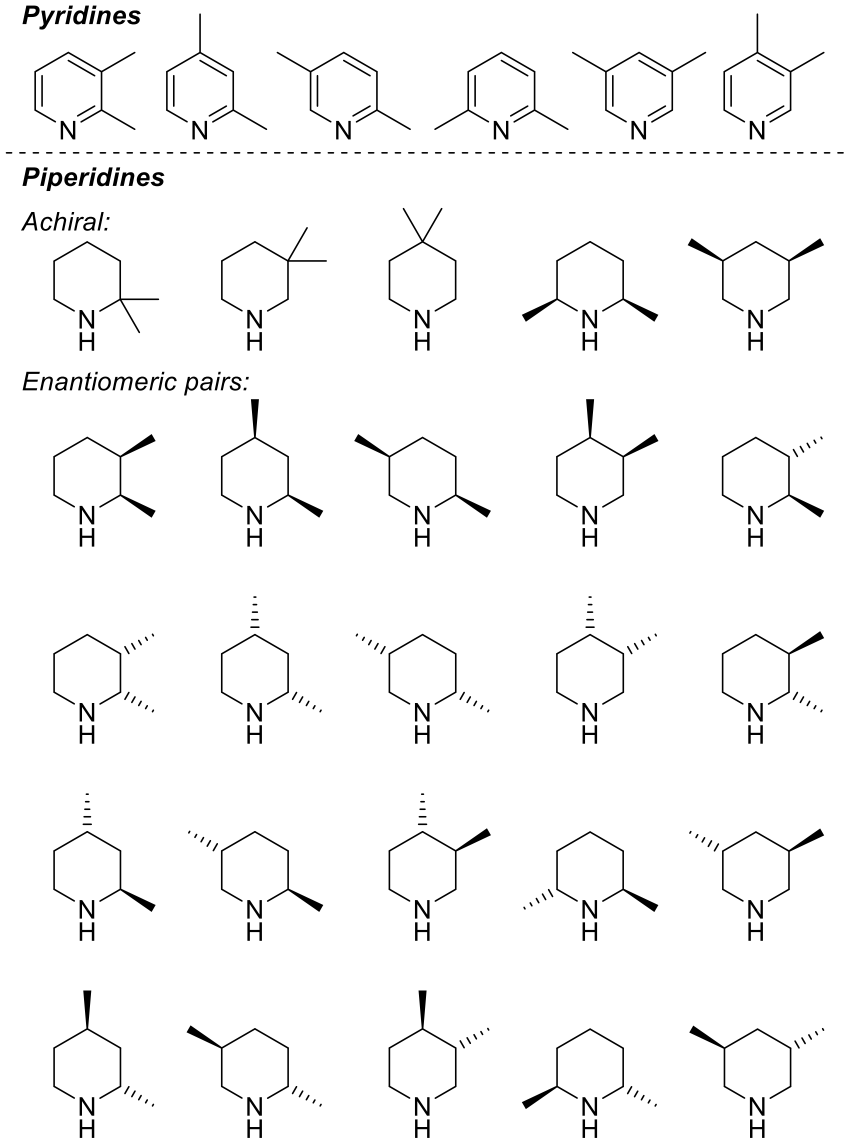

Figure S1. Isomers of dimethyl pyridine **1** and dimethyl NH piperidine **2**.

3. Virtual Library Analysis

3.1. Principle Moments of Inertia and Molecular Properties

Shape analysis

3-Dimensional structures of piperidines **6a-6d** were generated Pipeline Pilot 16.5.0.143, 2016, Accelrys Software Inc. Prior to conformer generation a wash step was performed, which involved stripping salts and ionising the molecule at pH 7.4. SMILES strings were converted to their canonical representation and the original stereochemistry at each chiral centre was recorded. Any stereocentre created during the ionisation would have undefined stereochemistry. A SMILES files was written that contained all possible stereoisomers of the molecule. Conformers were generated using Catalyst with the BEST conformational analysis method and relative stereochemistry. Catalyst was run directly on the server and not through the built-in Conformation Generator component. The maximum relative energy threshold was left at the default 20 kcal mol^{-1} and a maximum of 255 conformers were generated for each compound. The aim of this was to give the best possible coverage of conformational space. The resulting conformations from Catalyst were read and only those where the stereochemistry matched the original molecule or its enantiomer were kept. These were then all standardised to the original stereochemistry by mirroring the coordinates of the enantiomers. Duplicate conformations were filtered with a RMSD threshold of 0.1. Each conformation was minimised using 200 steps of Conjugate Gradient minimisation with an RMS gradient tolerance of 0.1. This was performed using the CHARMm forcefield with Momany-Rone partial charge estimation and a Generalised Born implicit solvent model. After minimisation, duplicates were filtered again with a RMSD threshold of 0.1. The lowest energy conformer of each molecule was selected and the generated conformations were used to generate the three Principal Moments of Inertia (I1, I2 and I3) which were then normalised by dividing the two lower values by the largest (I1/I3 and I2/I3) using Pipeline Pilot built-in components.

3-Dimensional structures of pyridines **3** were generated using RDKIT v4.5 in KNIME v4.4.1. A maximum of 50 conformers were generated for each molecule and the geometry of each was optimised using MMFF94 force field with 1000 iterations. Prior to calculation, salts were stripped and explicit hydrogens added. The lowest energy conformer of each molecule was selected and the three Principal Moments of Inertia calculated using Vernalis PMI KNIME nodes.

Principal moments of inertia (PMI) about the principal axes of a molecule were calculated according to the following rules:

1. The moments of inertia are computed for a series of straight lines through the centre of mass.

2. Distances are established along each line proportional to the reciprocal of the square root of I on either side of the centre of mass. The locus of these distances forms an ellipsoidal surface. The principal moments are associated with the principal axes of the ellipsoid.

Molecular properties

Heavy atom count (HAC) was calculated using RDKit v3.4 in KNIME v3.5.2. Prior to calculation, salts were stripped. ClogP values were calculated using Daylight/BioByte ClogP v4.3.

3.2 Virtual Library Enumeration and Lead-Likeness Analysis

To assess the suitability of our piperidines for the generation of lead-like compounds we used the open access tool LLAMA.[14,15] Using our deprotected piperidines (as amino acids) as scaffolds, LLAMA computationally decorated (once or twice) the amine and acid functionalities and analysed the resulting virtual molecules for their lead-likeness (for leading examples of lead-oriented synthesis see[16–19]). LLAMA generated 1599 virtual compounds from our 20 deprotected piperidines. The default set of capping groups was used, and of the standard decoration reactions, “Secondary amide alkylation” and “Secondary amide arylation” were disabled to prevent two diversification events occurring at the same diversification point of the scaffolds.

Of the 1599 virual compounds, 78% fell within lead-like space (defined as molecular weight between 200 and 350, and AlogP between -1 and 3)[14] (Figure S2). For comparison, only 23% of the ZINC database of commercially available screening compounds fall within lead-like space.[14,17] LLAMA also generates a lead-likeness penalty (LLP) score for each molecule (the lower the better), considering the number of aromatic rings and the presence of unwanted structural features as well as molecular weight and AlogP. Our virtual library had a mean LLP of 1.02 (*cf.* 4.17 for ZINC) indicating that compounds derived from di-substituted piperidines **5** would be highly lead-like. Furthermore, analysis of the degree of saturation of the virtual library showed that it had a mean Fsp^3 of 0.62, whereas the mean value for the Zinc library is only 0.33.

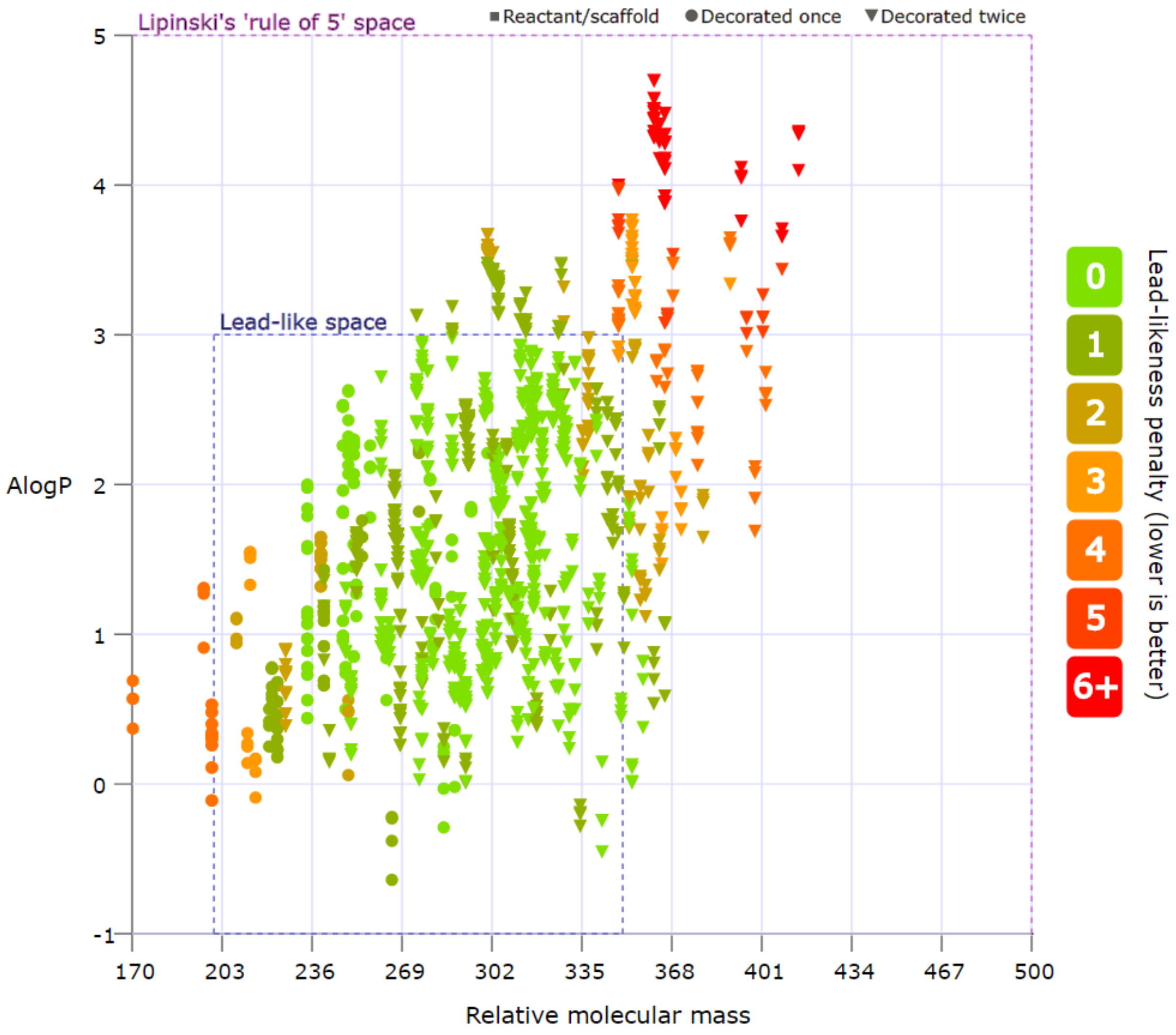

Figure S2. Molecular properties of the virtual lead-like library (coloured per lead likeness penalty: 0, green; 3, orange; 6+ red).

4. ^{1}H and ^{13}C NMR Spectra

400 MHz ^{1}H NMR spectrum; 100.6 MHz ^{13}C NMR spectrum; $CDCl_3$

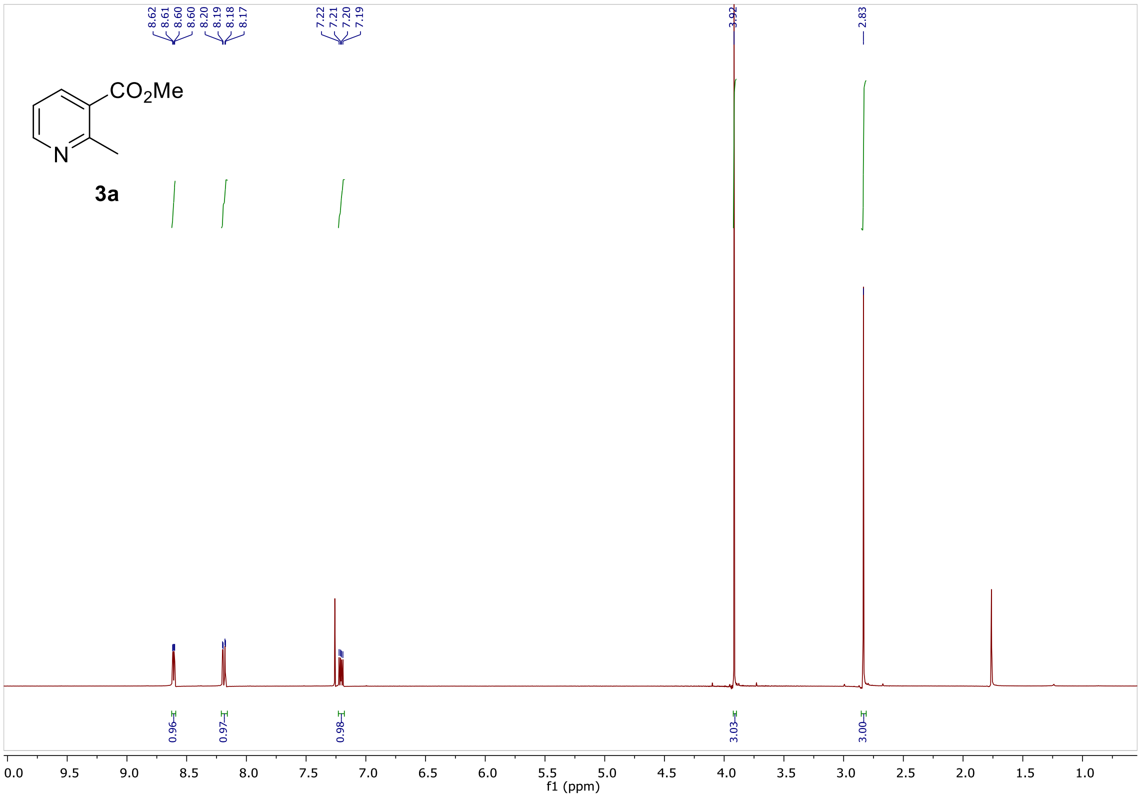

3a

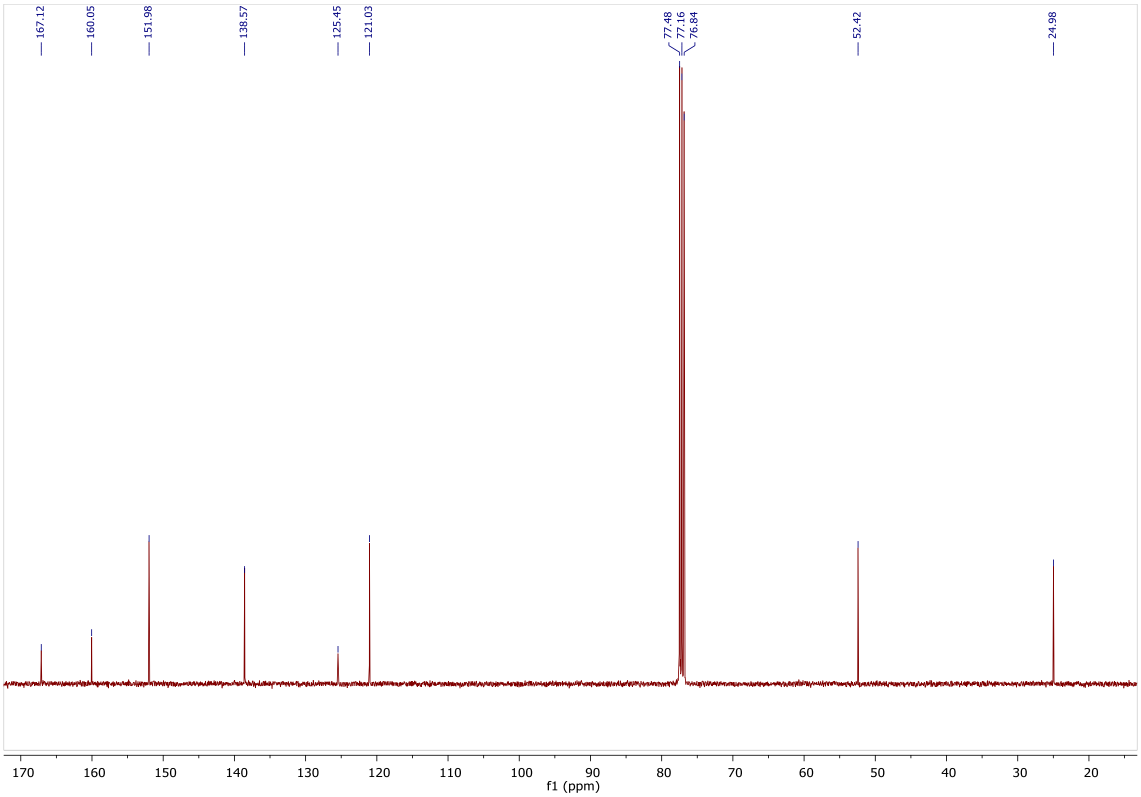

400 MHz ^1H NMR spectrum; 100.6 MHz ^{13}C NMR spectrum; $CDCl_3$

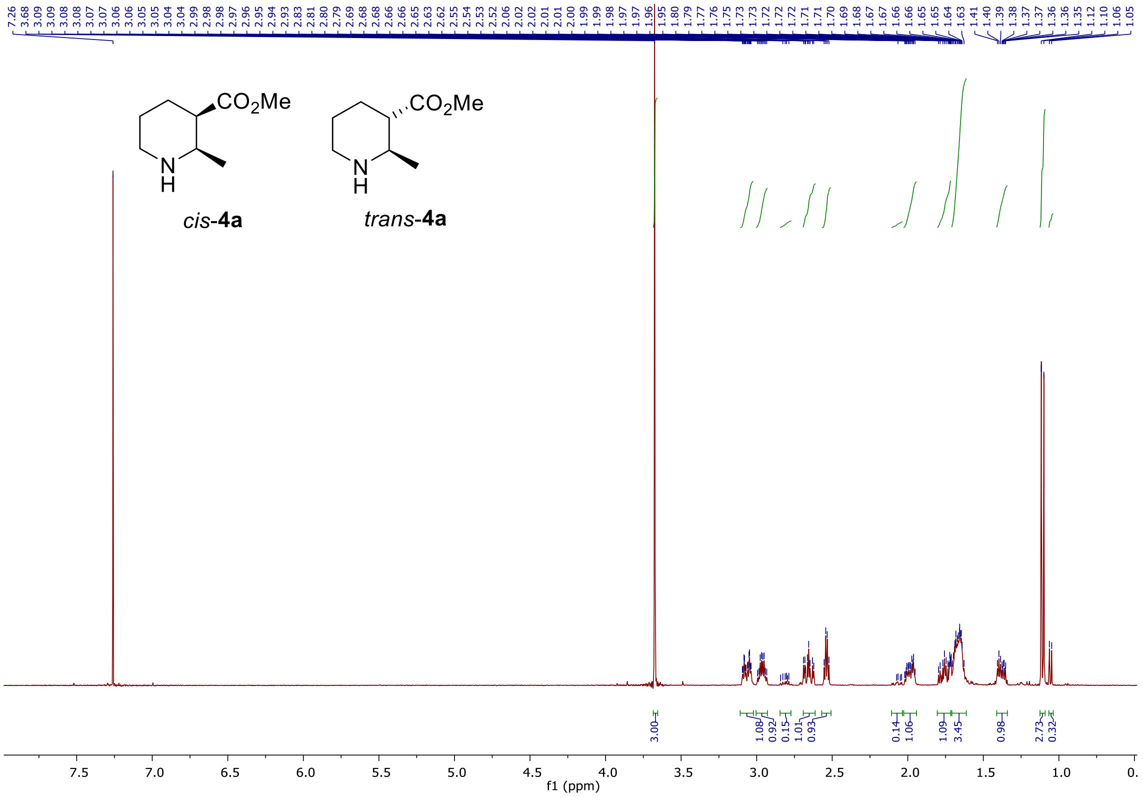

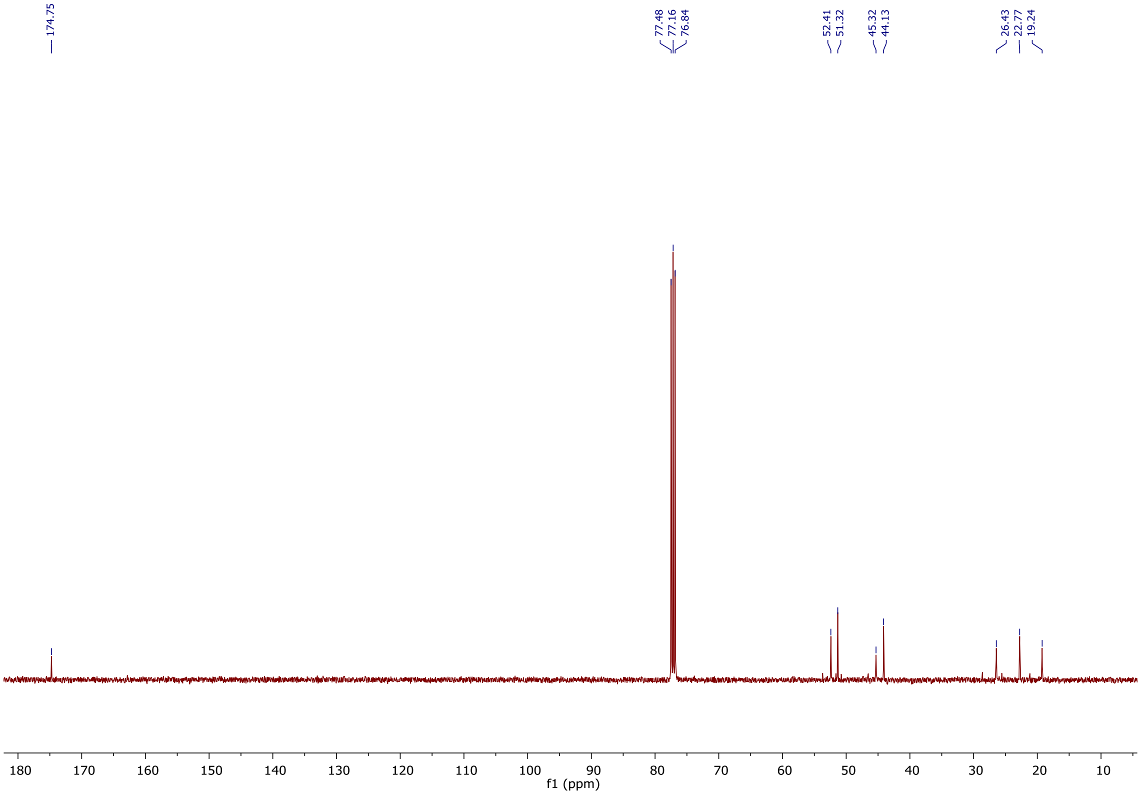

400 MHz ^{1}H NMR spectrum; 100.6 MHz ^{13}C NMR spectrum; $CDCl_3$

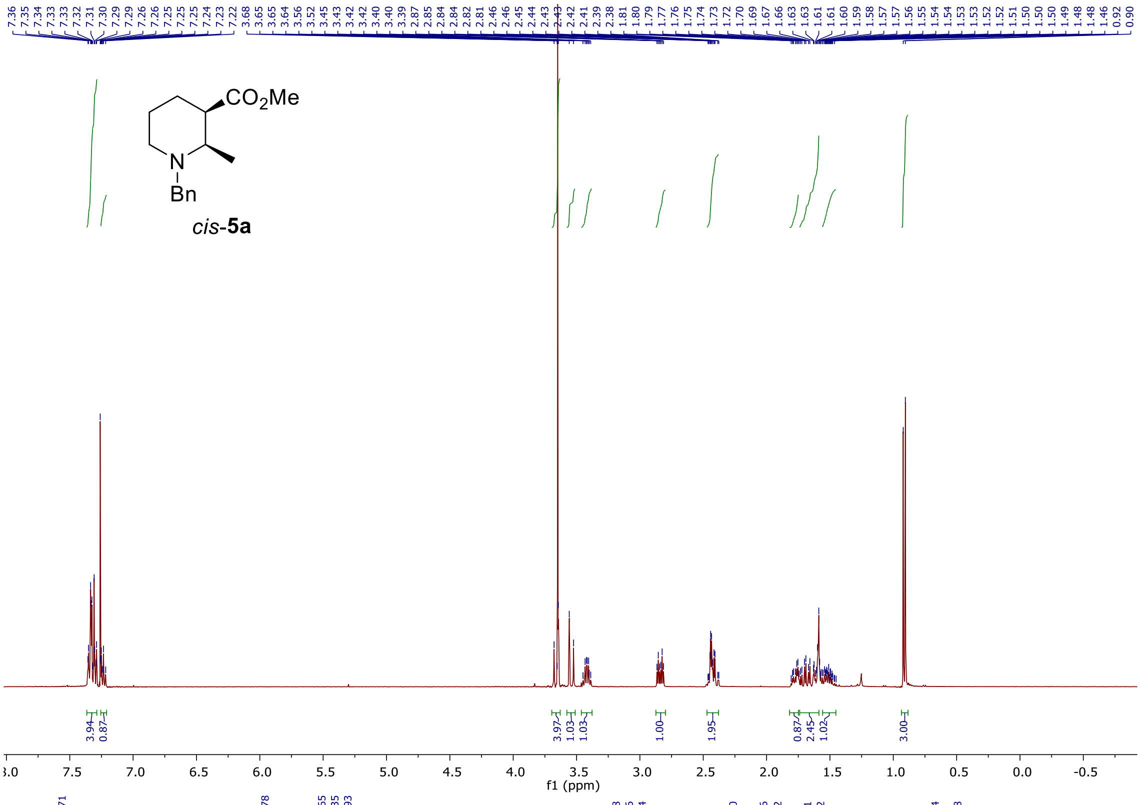

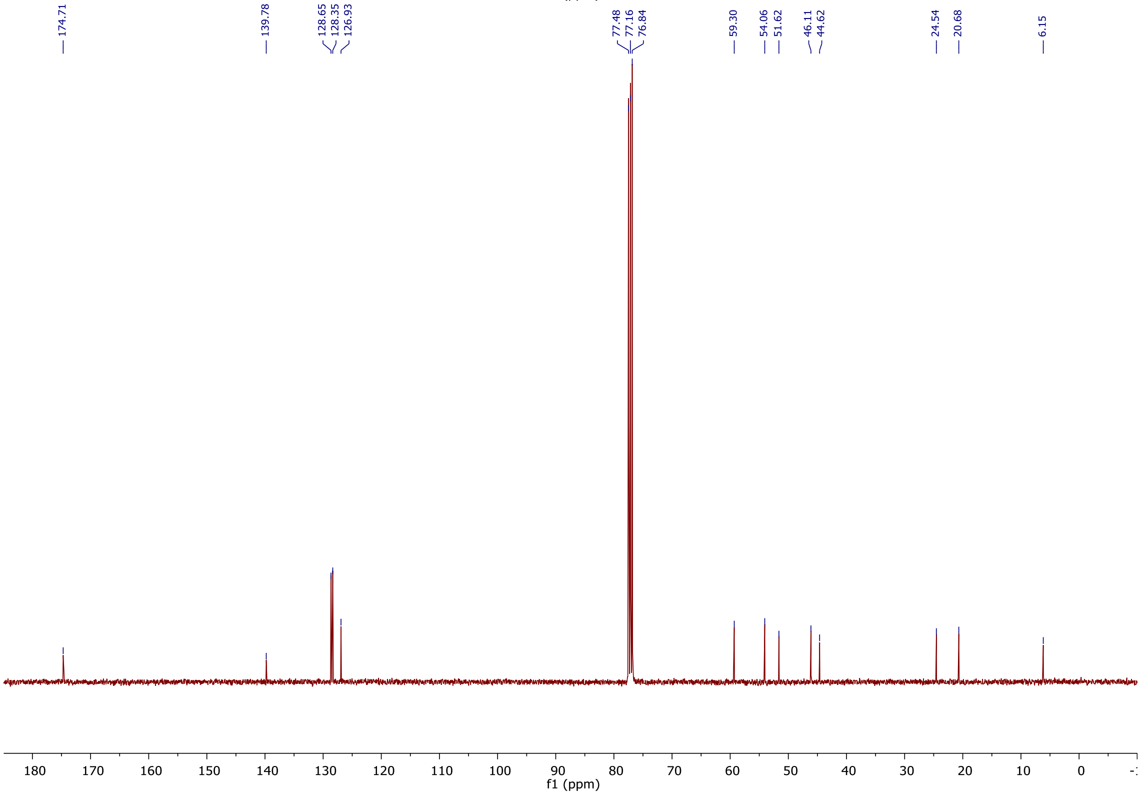

400 MHz ^{1}H NMR spectrum; 100.6 MHz ^{13}C NMR spectrum; $CDCl_3$

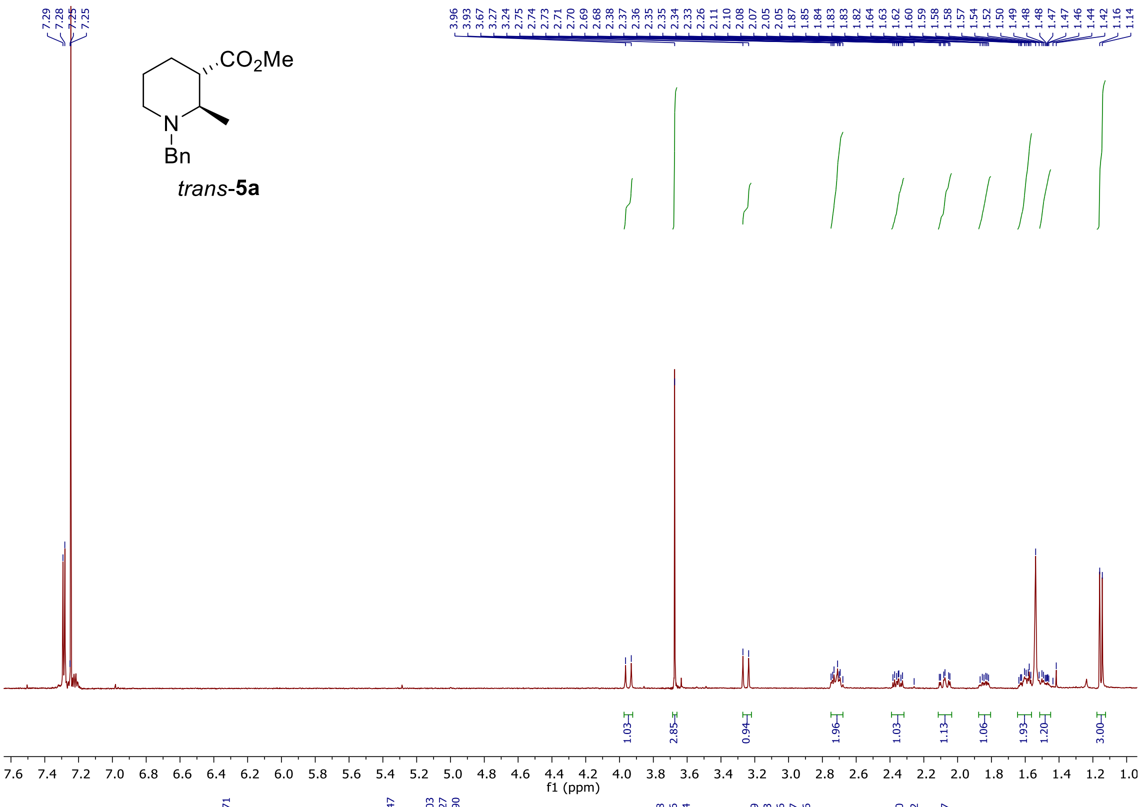

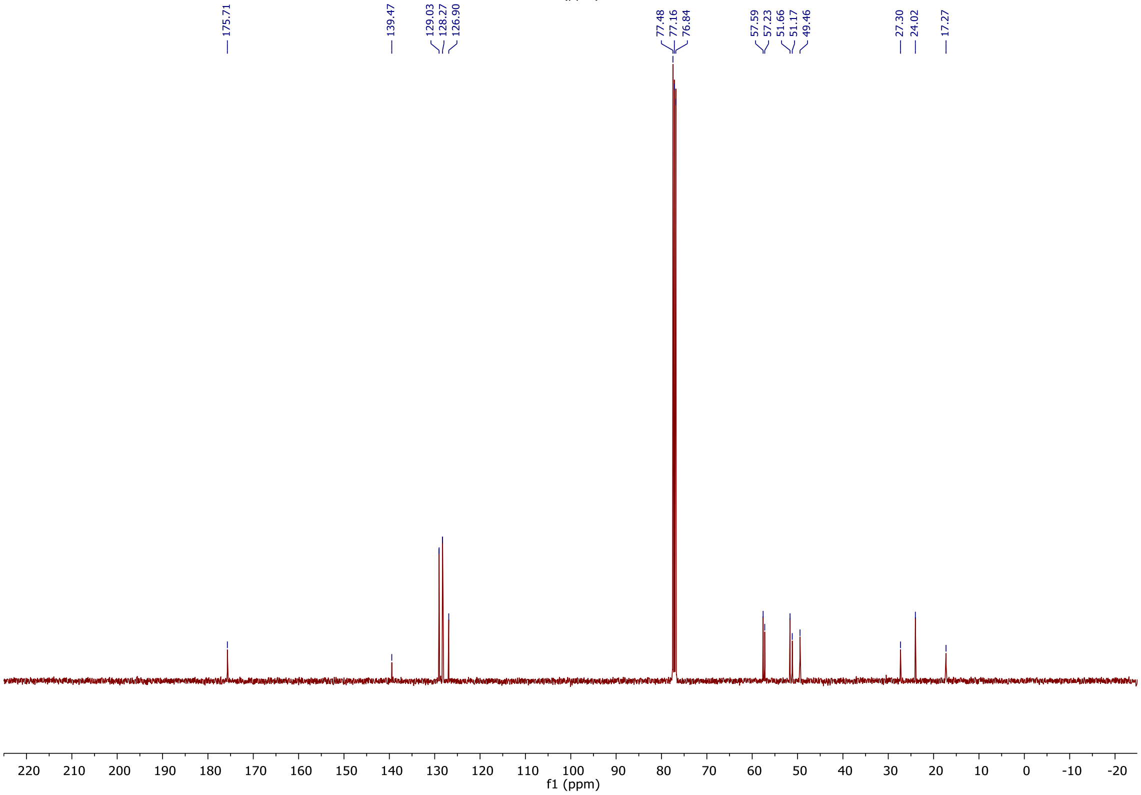

400 MHz ^1H NMR spectrum; 100.6 MHz ^{13}C NMR spectrum; $CDCl_3$

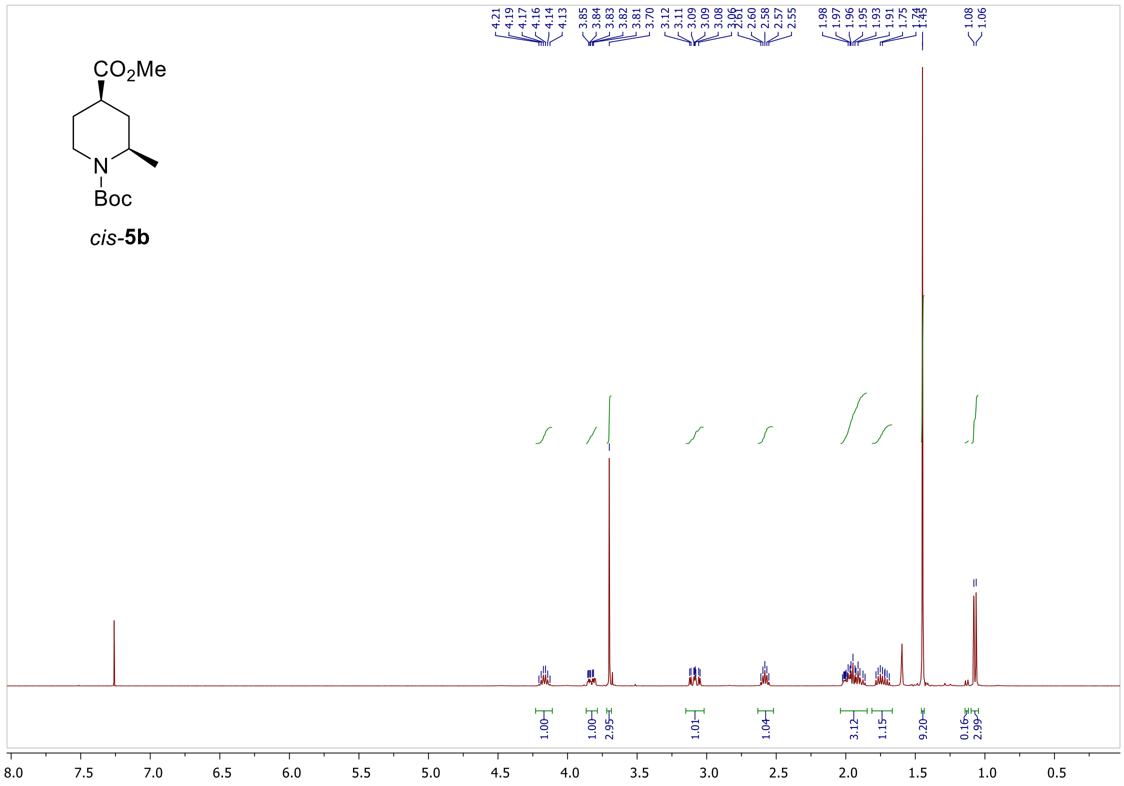

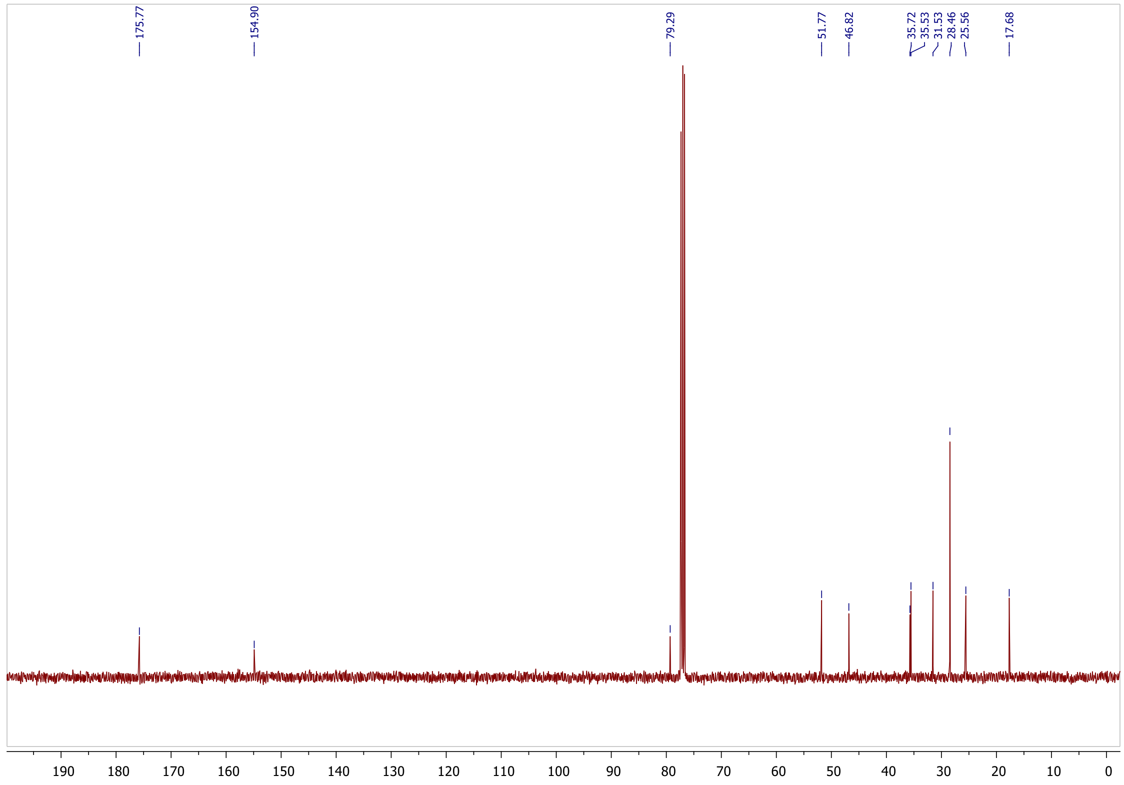

400 MHz ^1H NMR spectrum; 100.6 MHz ^{13}C NMR spectrum; $CDCl_3$

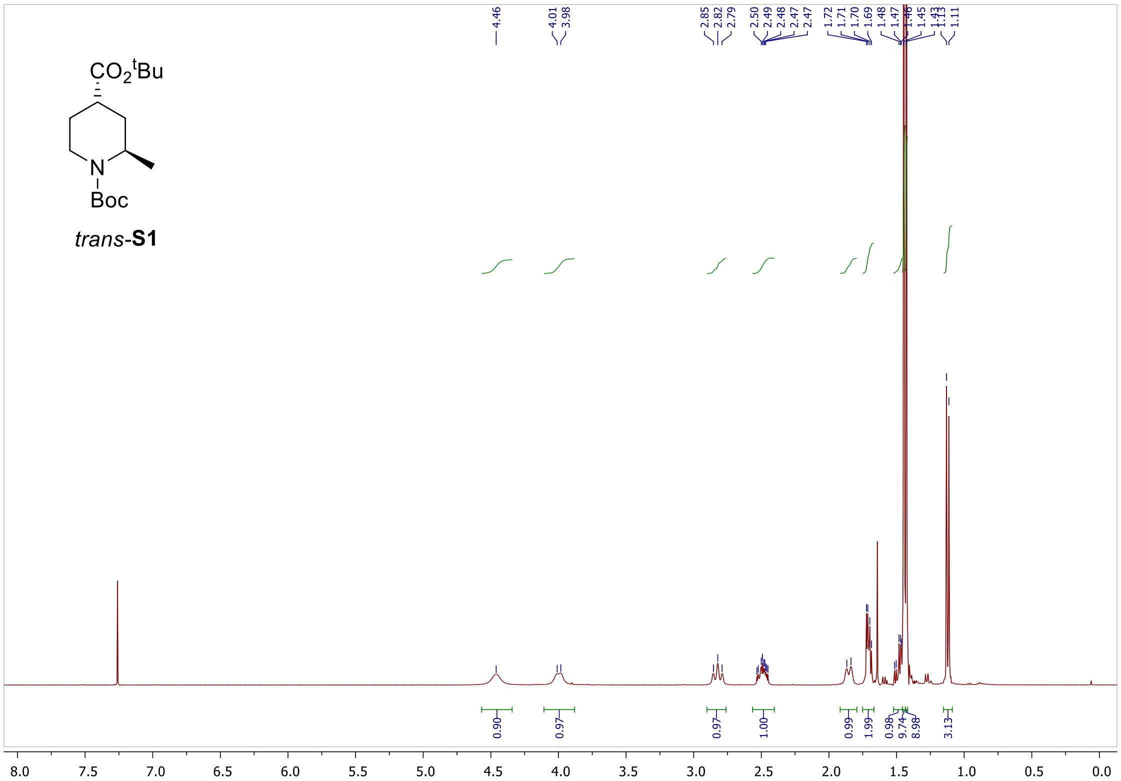

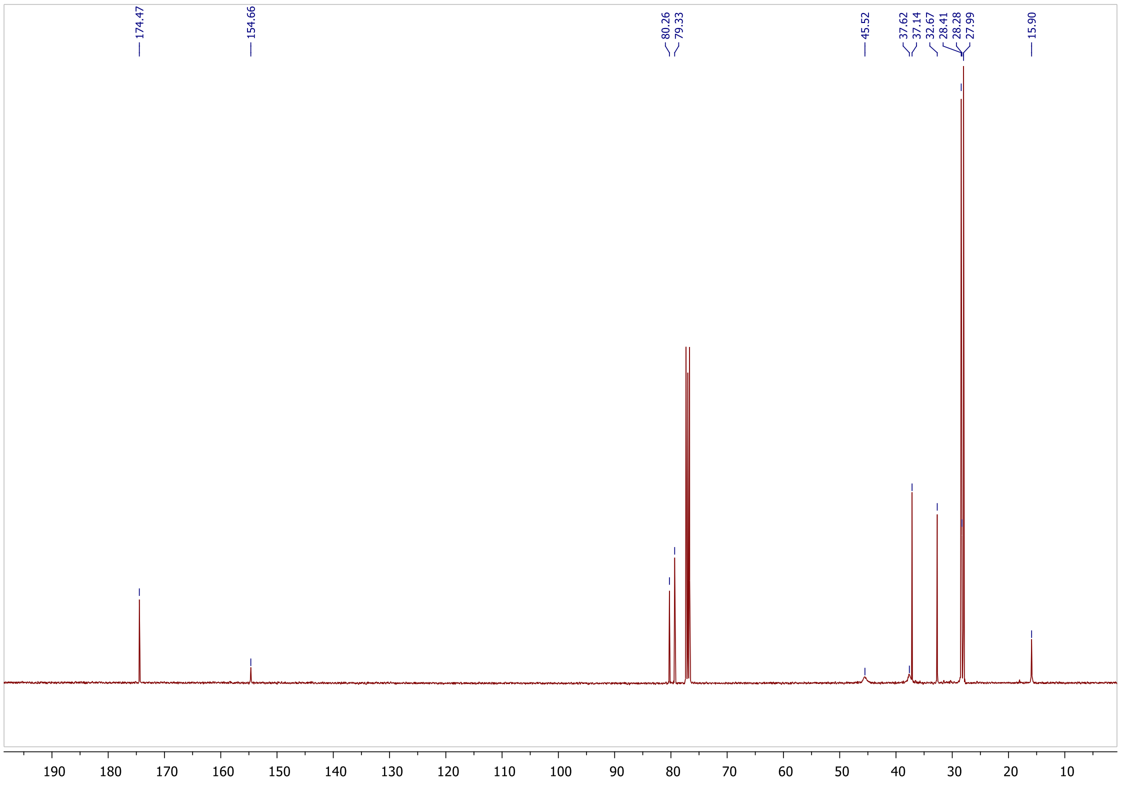

400 MHz ^{1}H NMR spectrum; 100.6 MHz ^{13}C NMR spectrum; $CDCl_3$

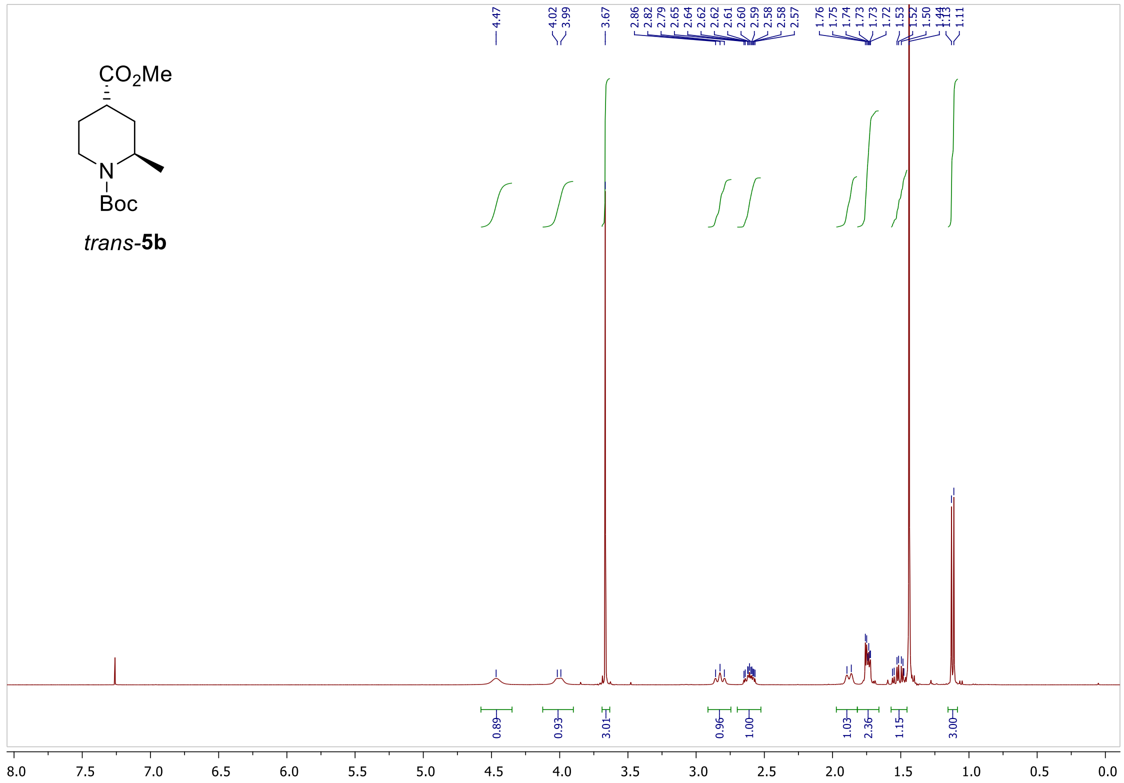

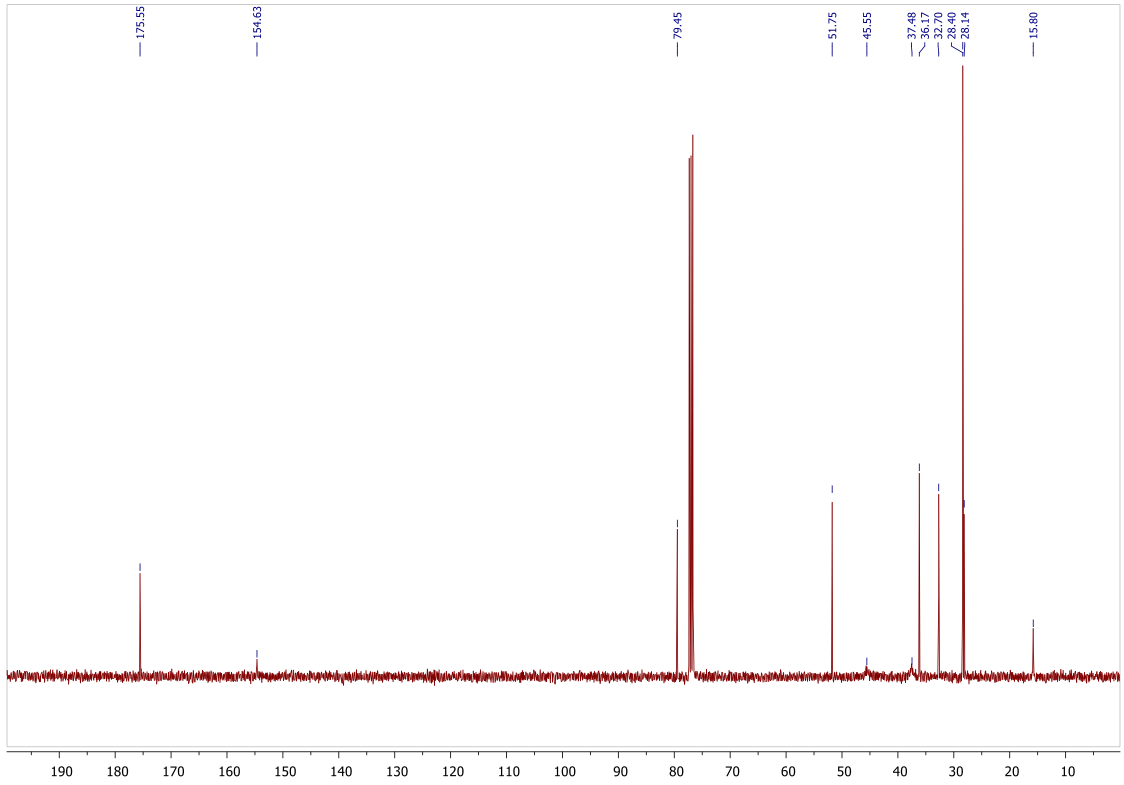

400 MHz ^{1}H NMR spectrum; 100.6 MHz ^{13}C NMR spectrum; $CDCl_3$

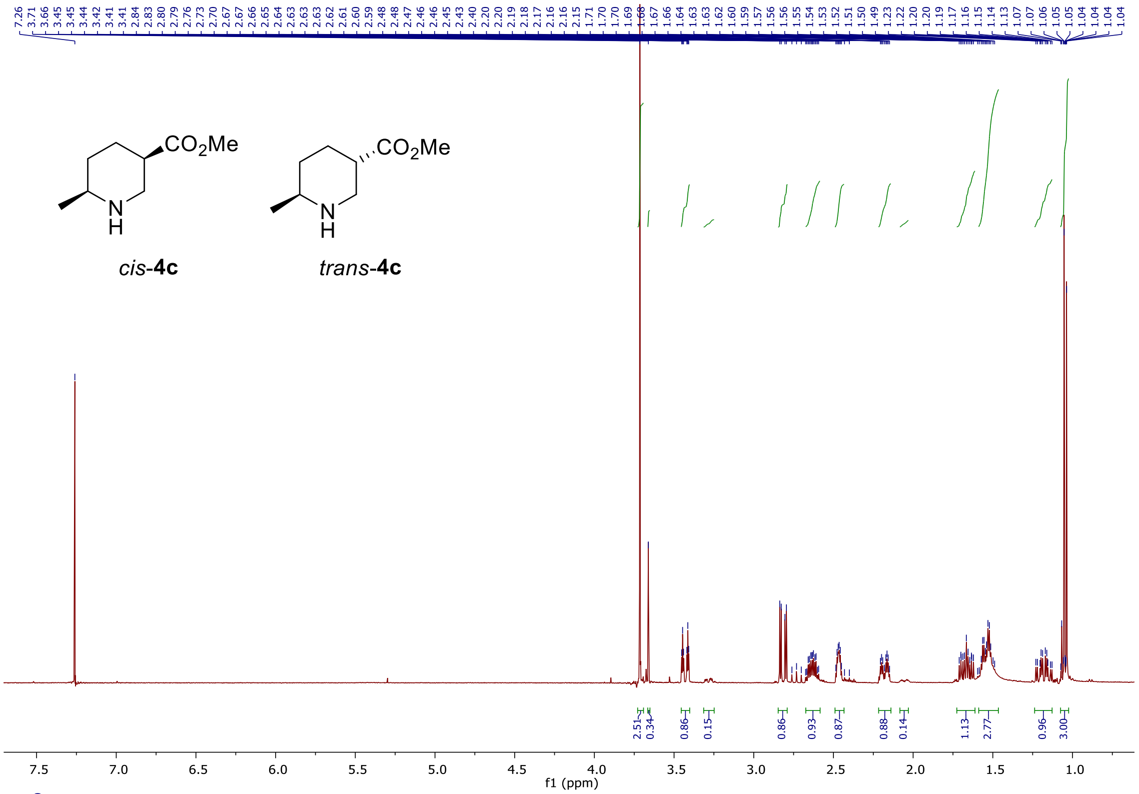

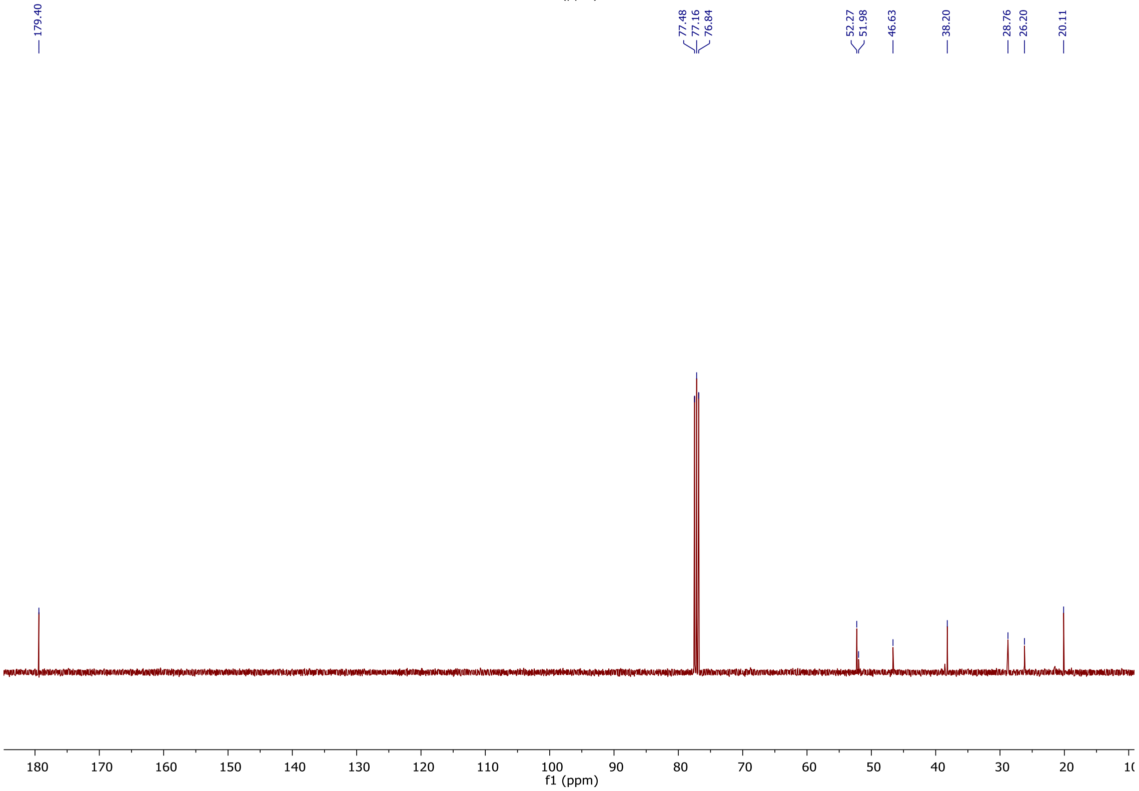

400 MHz ^1H NMR spectrum; 100.6 MHz ^{13}C NMR spectrum; $CDCl_3$

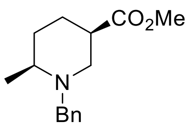

cis-**5c**

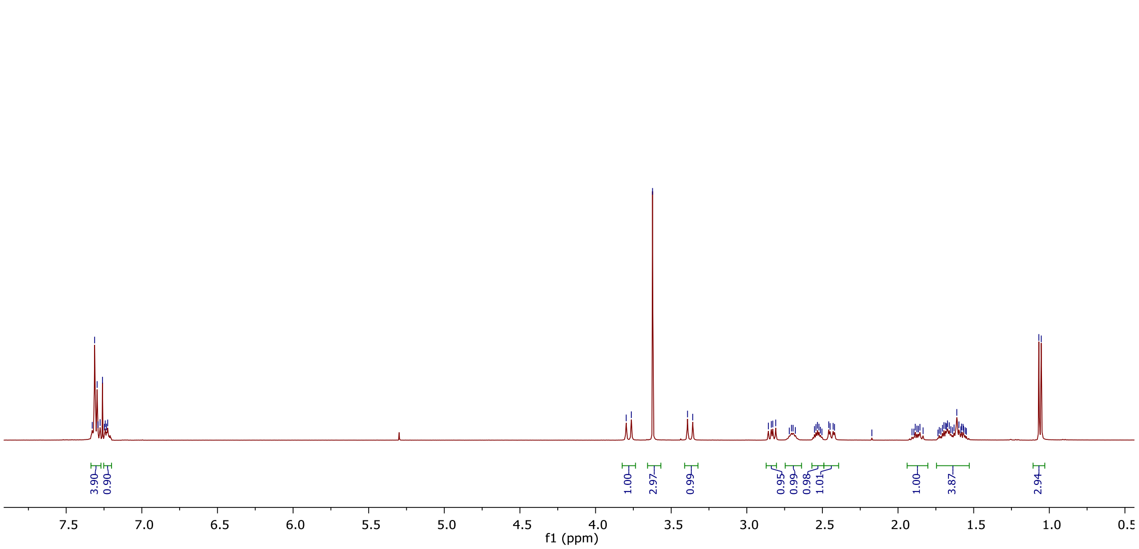

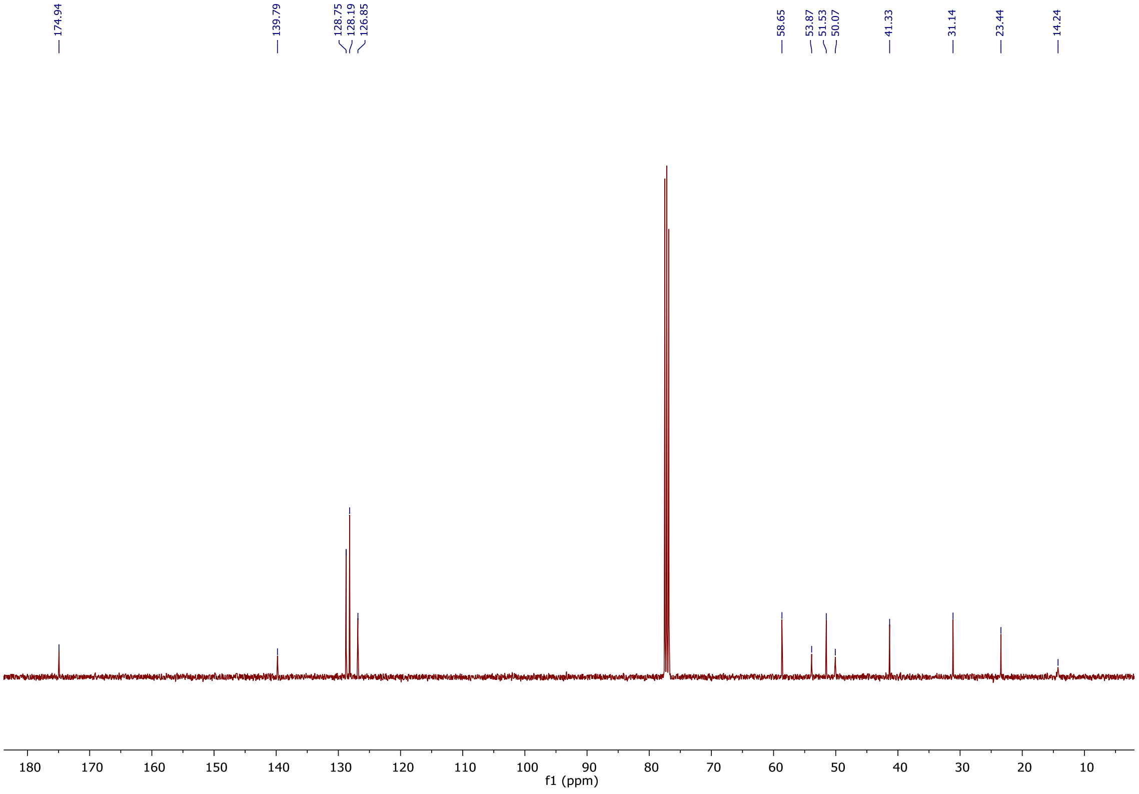

400 MHz ^{1}H NMR spectrum; 100.6 MHz ^{13}C NMR spectrum; $CDCl_3$

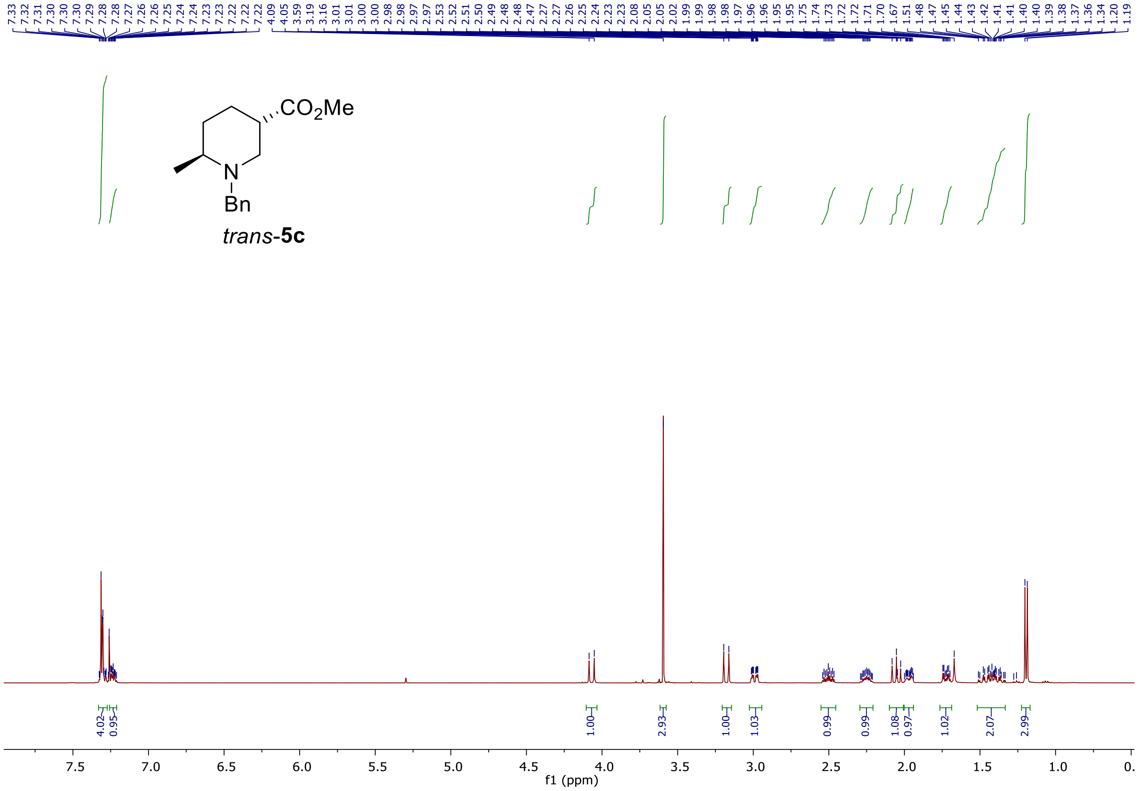

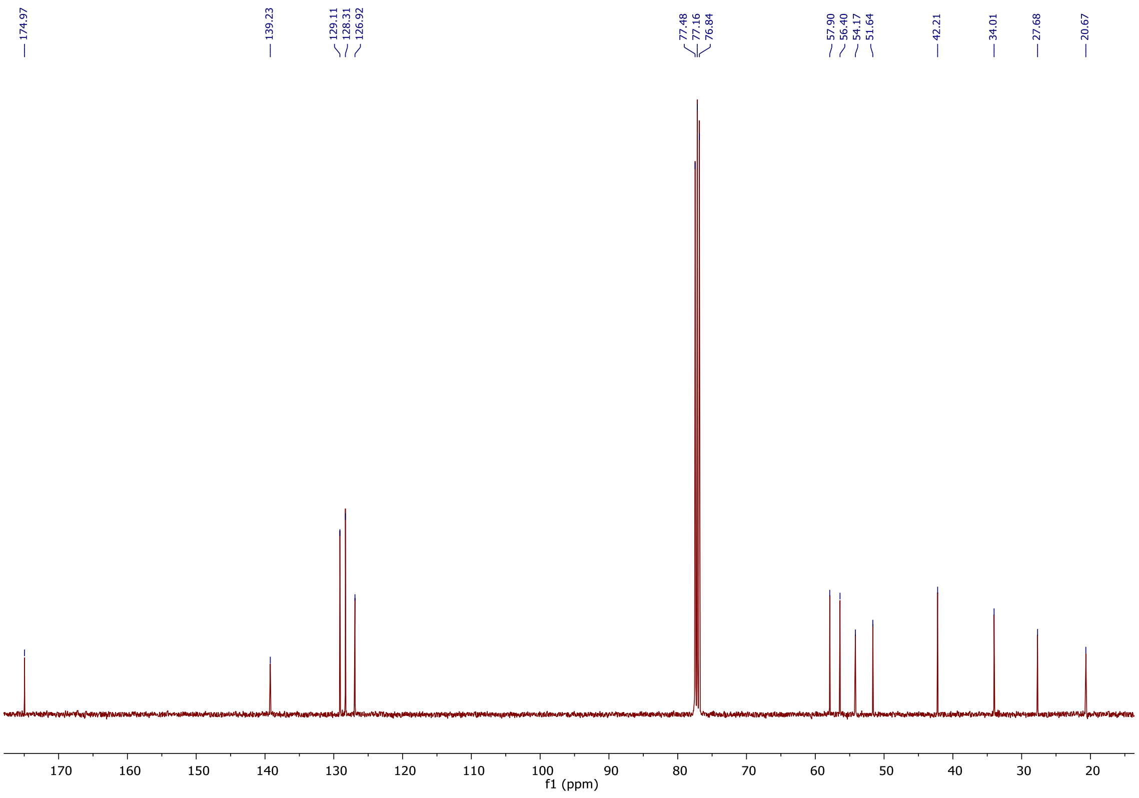

400 MHz ^{1}H NMR spectrum; 100.6 MHz ^{13}C NMR spectrum; $CDCl_3$

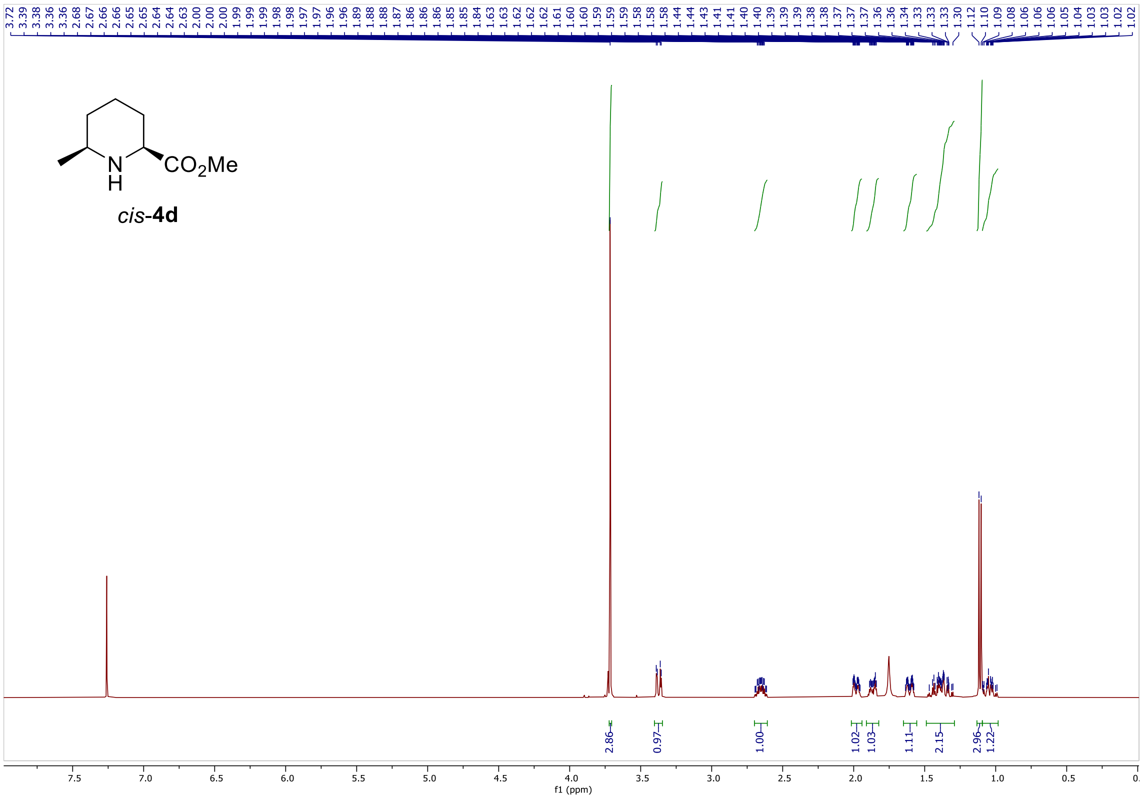

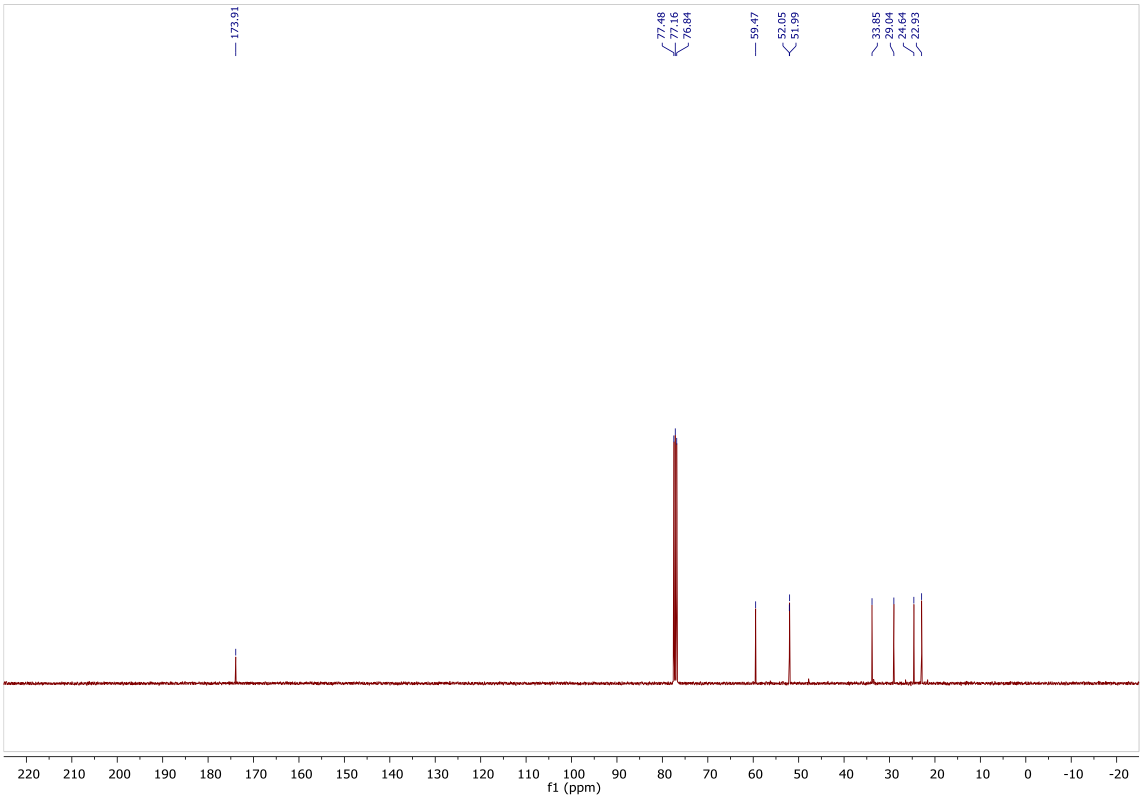

400 MHz ^{1}H NMR spectrum; 100.6 MHz ^{13}C NMR spectrum; $CDCl_3$

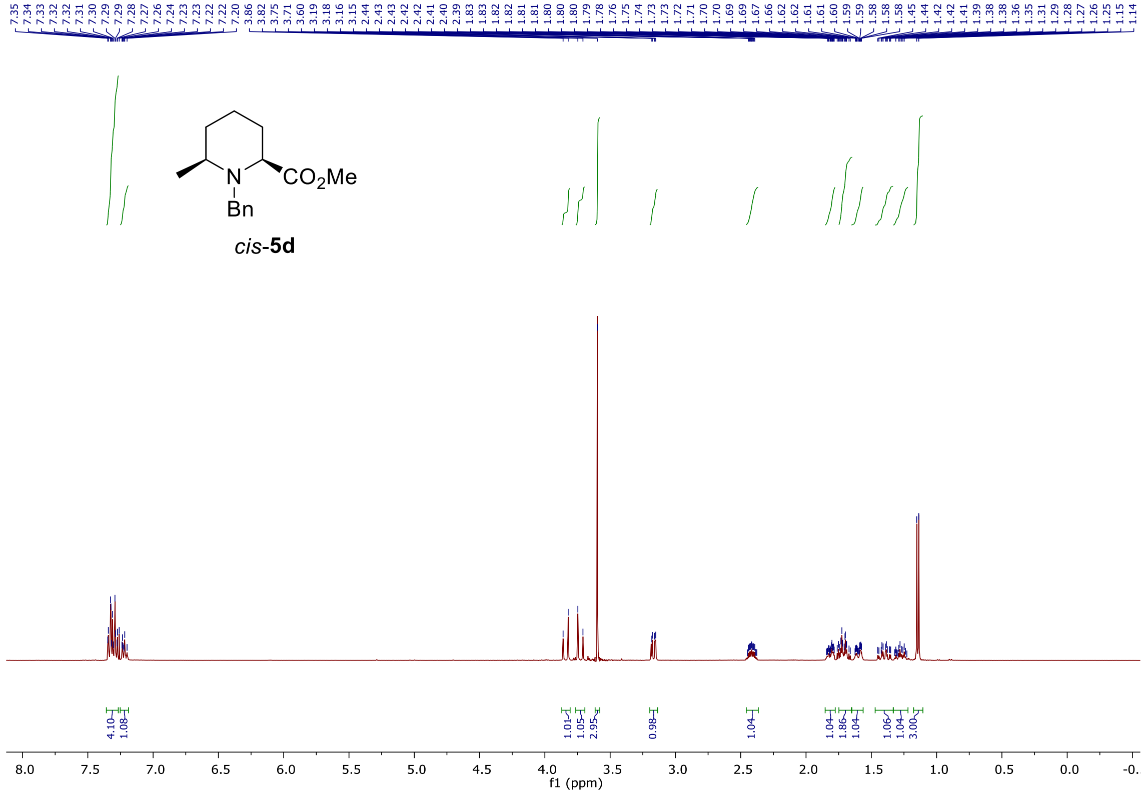

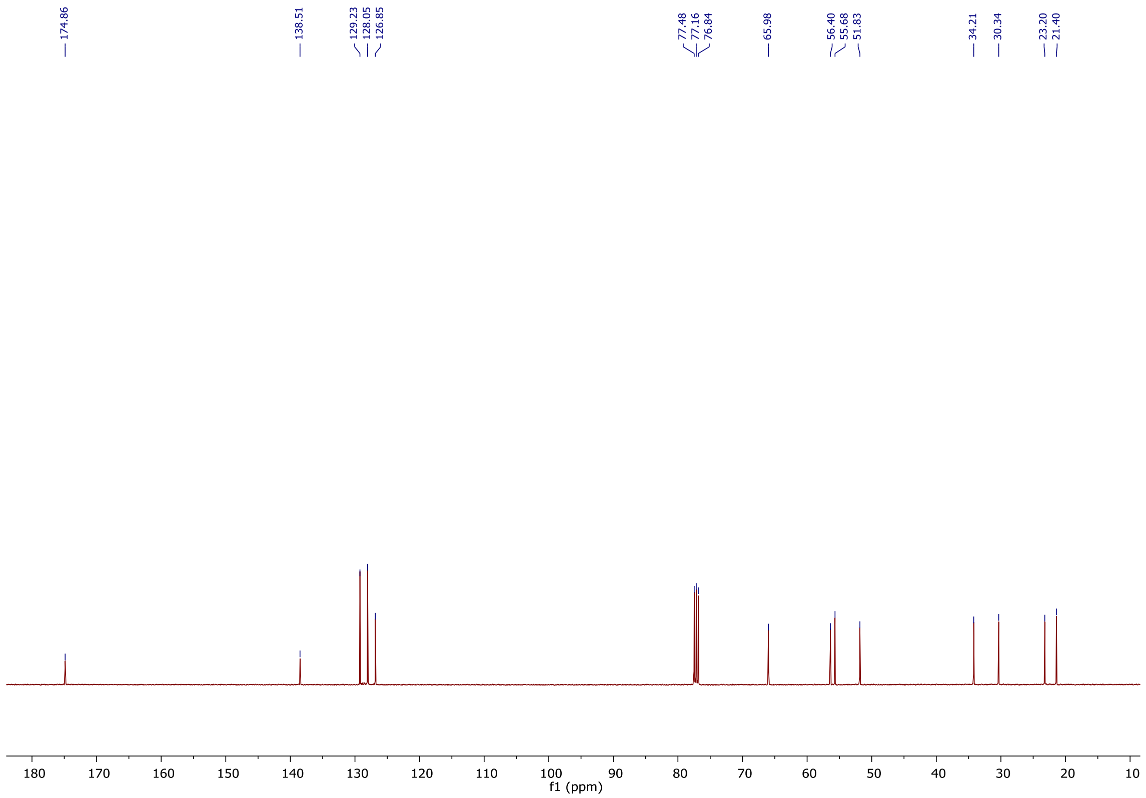

400 MHz ^{1}H NMR spectrum; 100.6 MHz ^{13}C NMR spectrum; $CDCl_3$

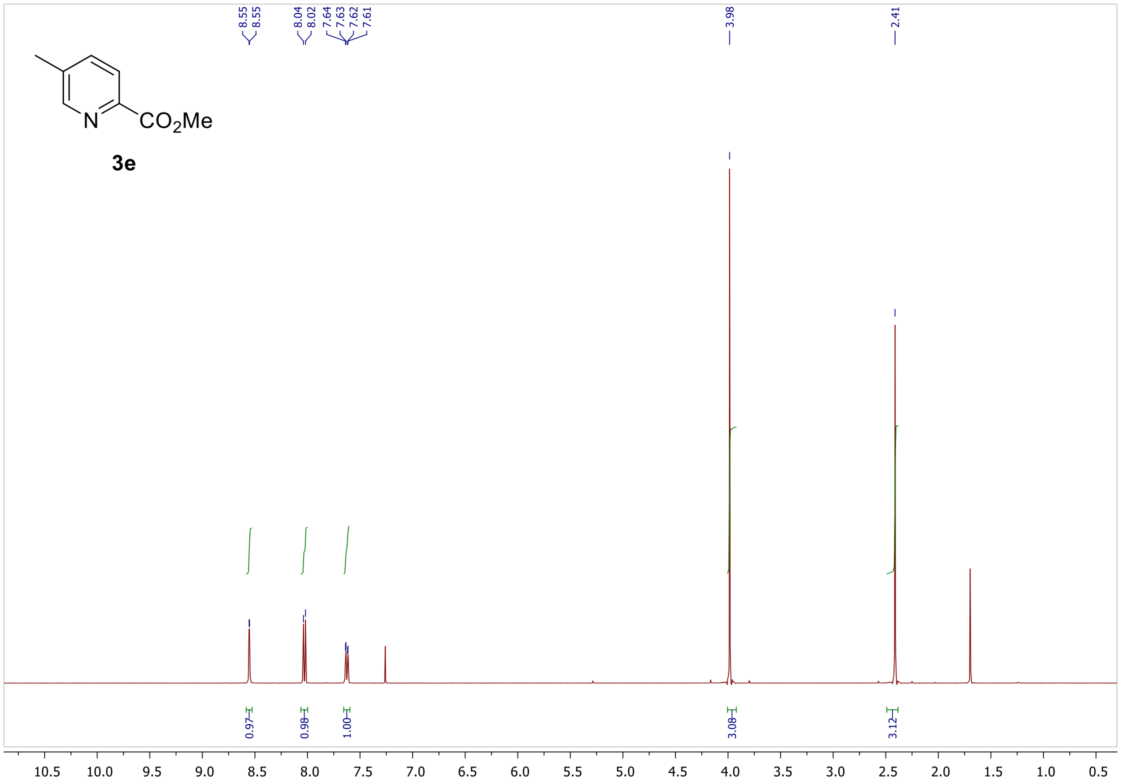

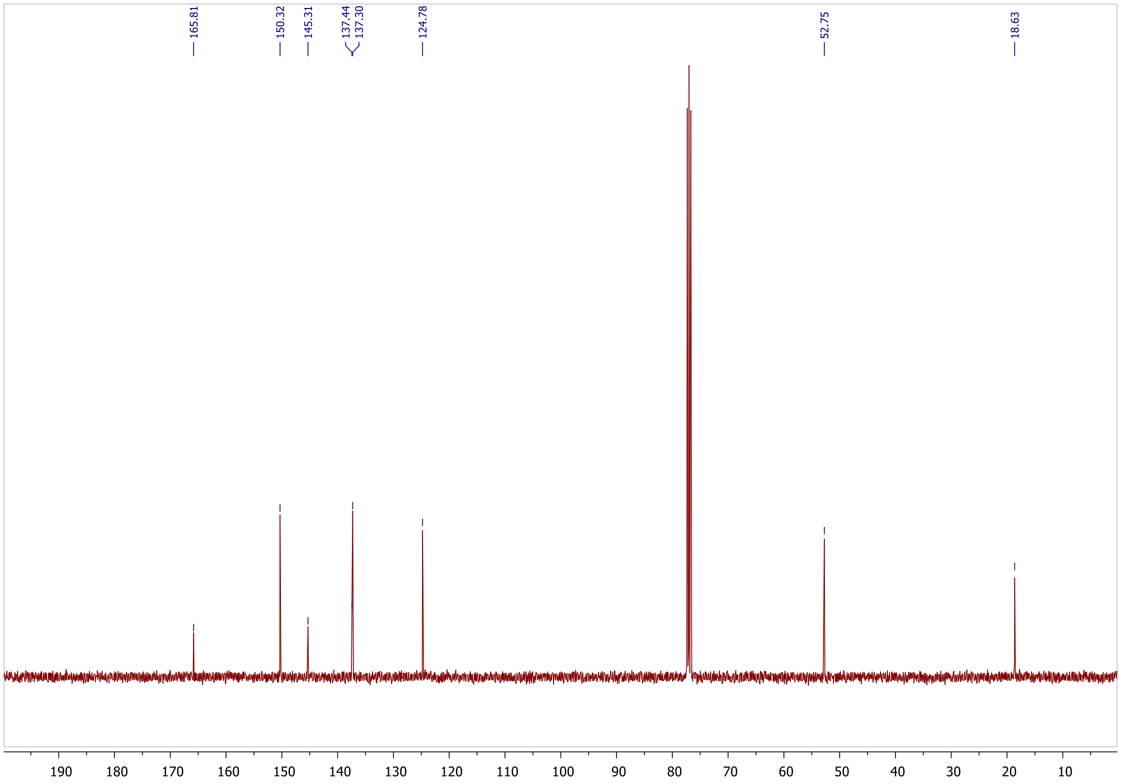

400 MHz ^1H NMR spectrum; 100.6 MHz ^{13}C NMR spectrum; $CDCl_3$

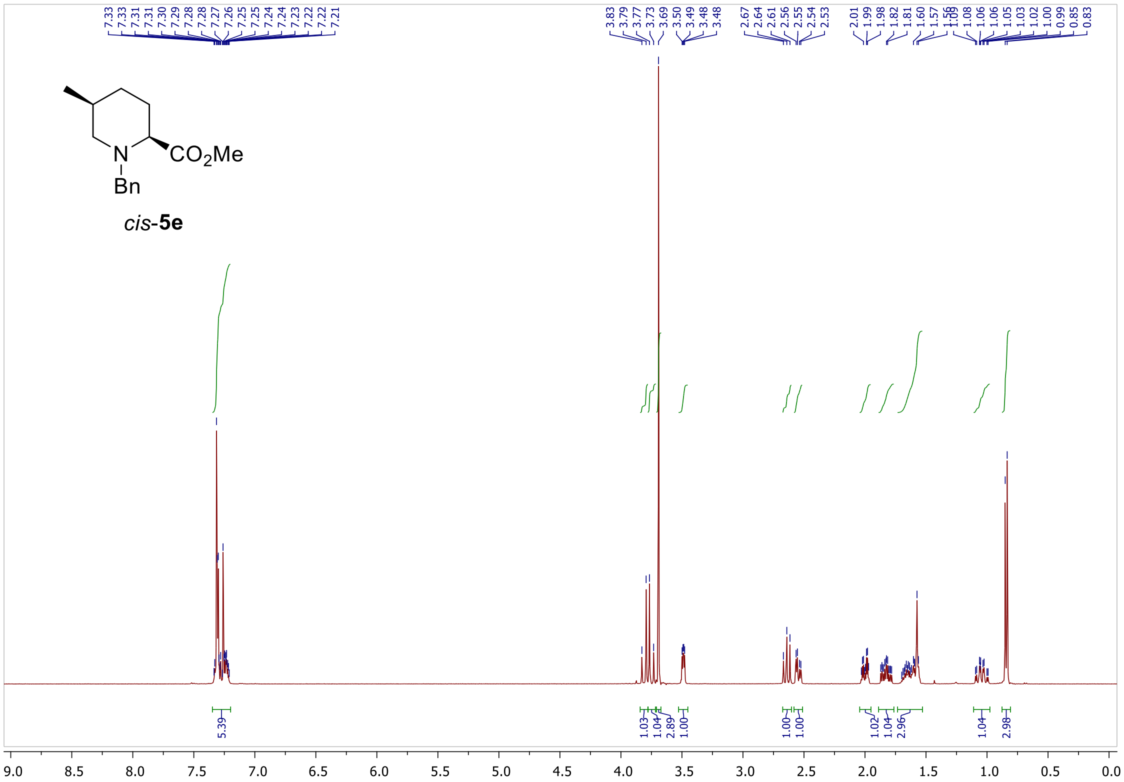

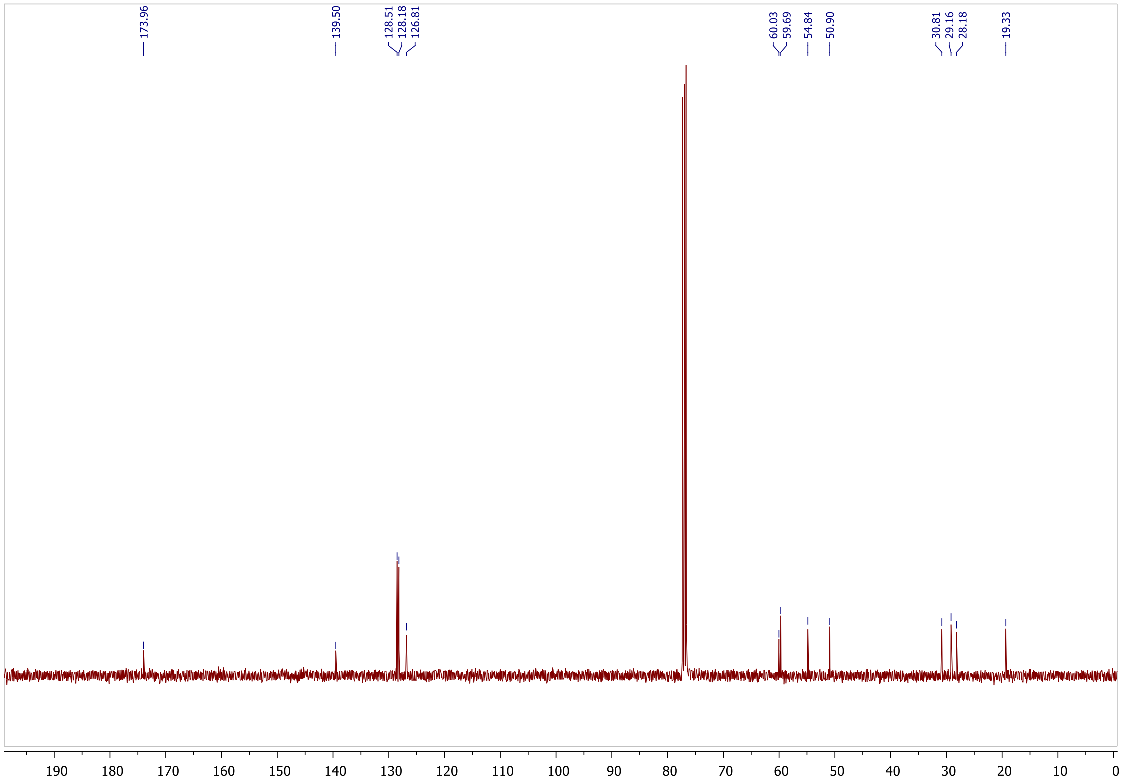

400 MHz ^1H NMR spectrum; 100.6 MHz ^{13}C NMR spectrum; $CDCl_3$

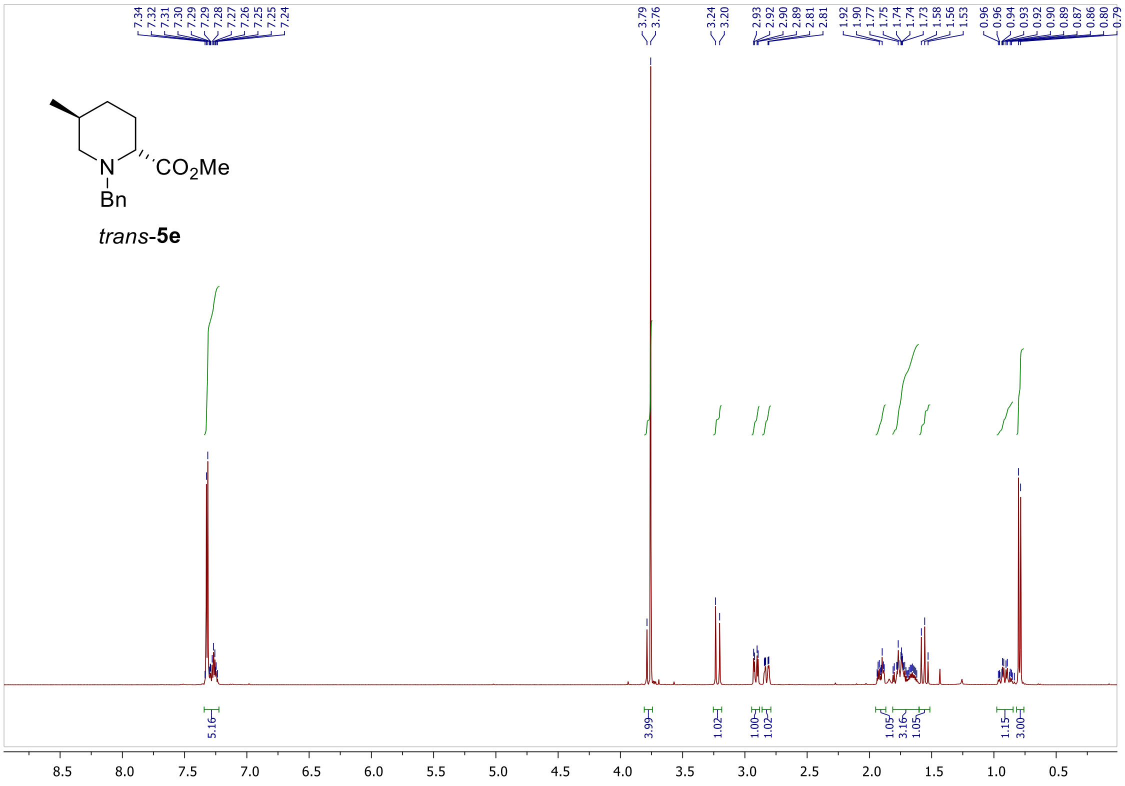

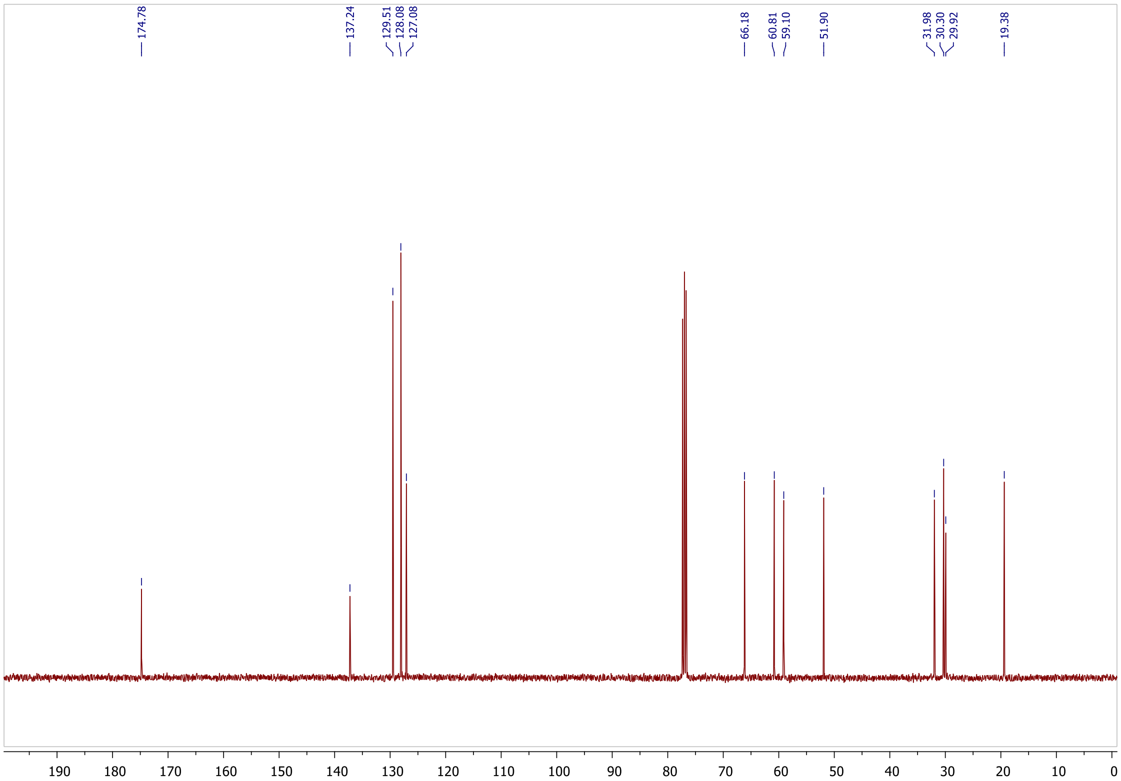

400 MHz ^{1}H NMR spectrum; 100.6 MHz ^{13}C NMR spectrum; $CDCl_3$

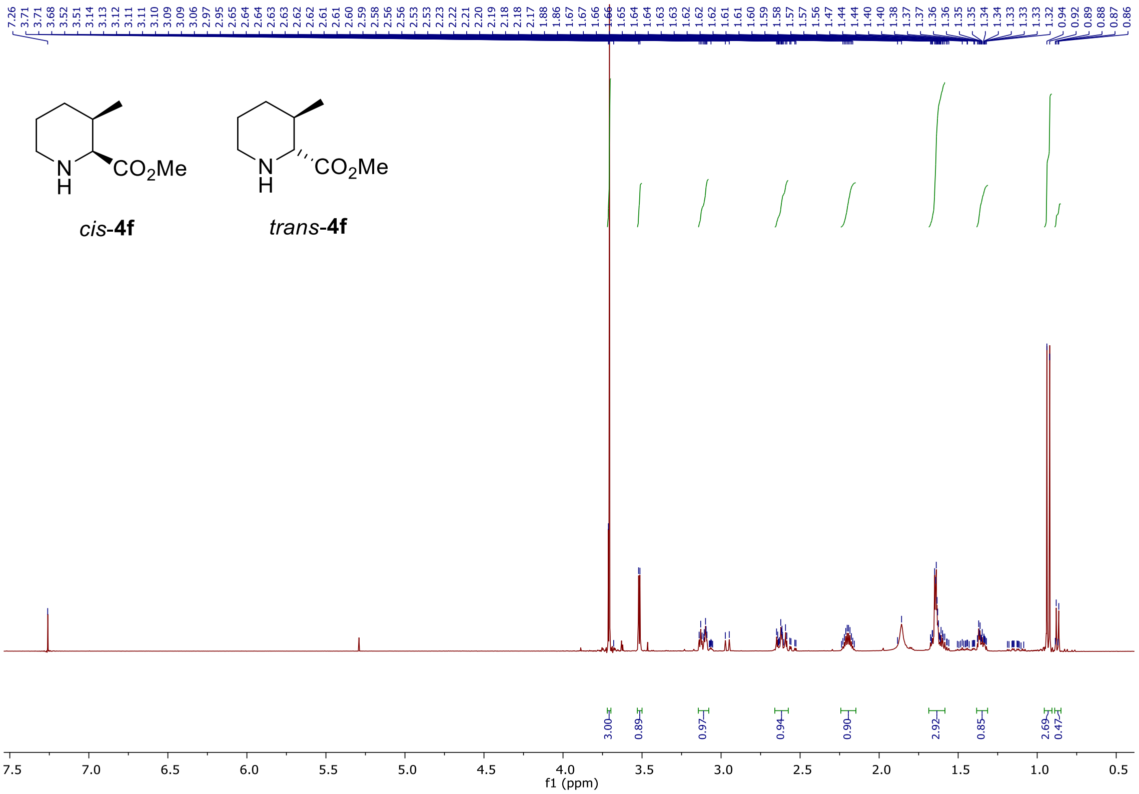

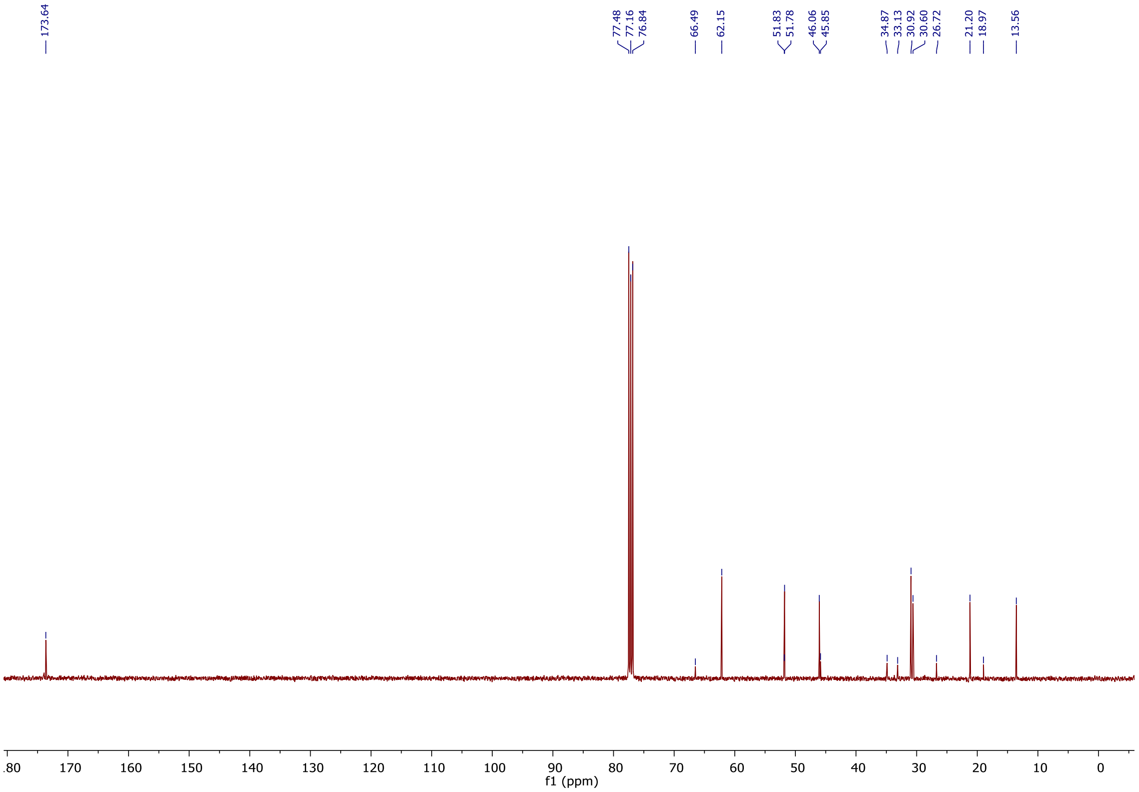

400 MHz ^{1}H NMR spectrum; 100.6 MHz ^{13}C NMR spectrum; $CDCl_3$

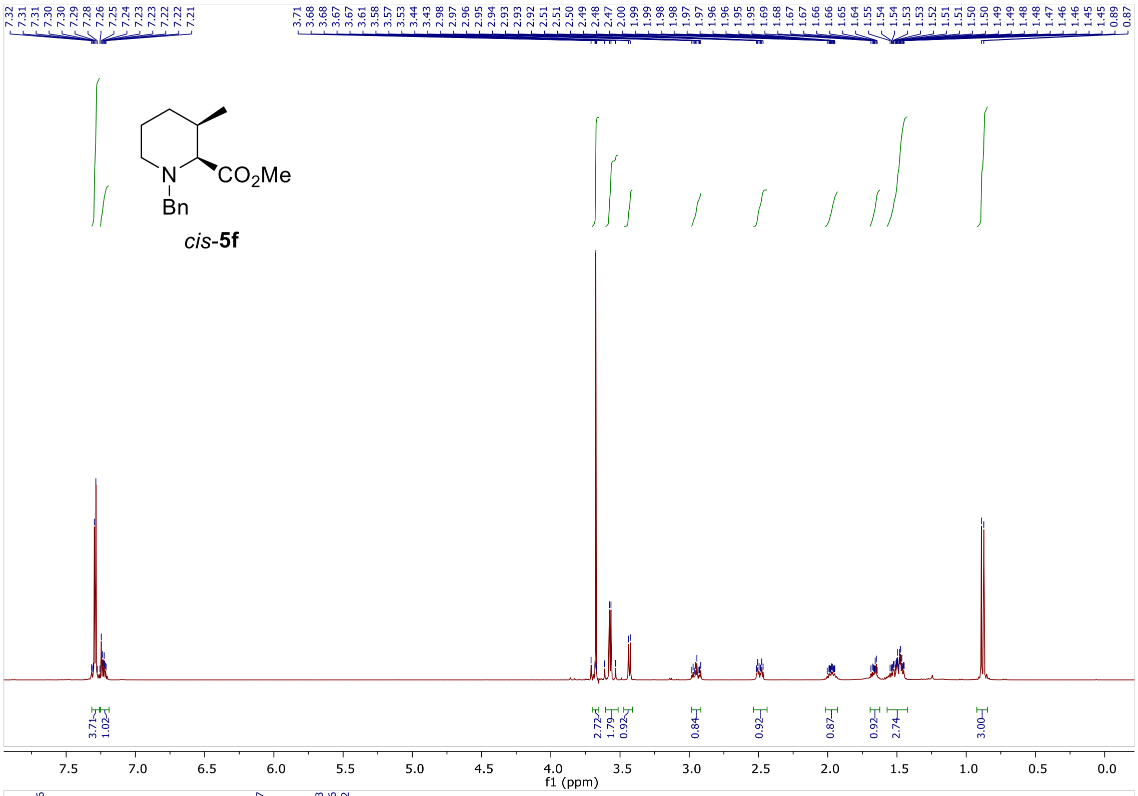

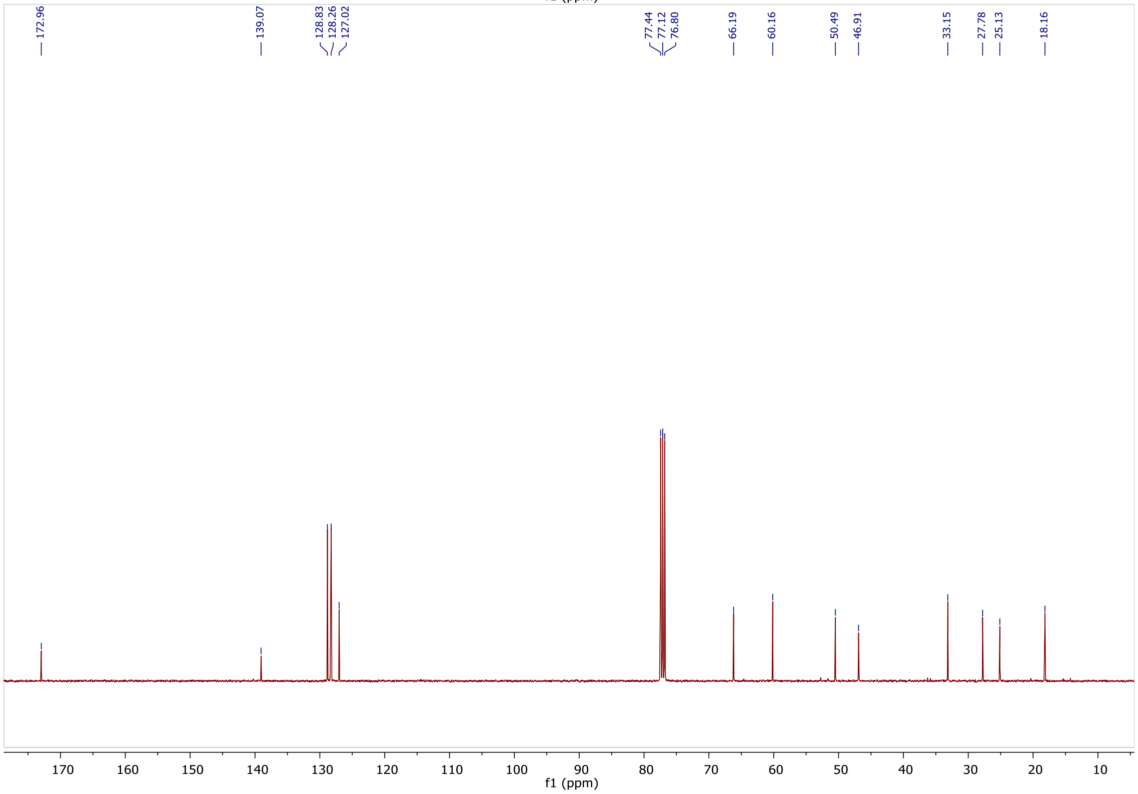

400 MHz ^{1}H NMR spectrum; 100.6 MHz ^{13}C NMR spectrum; $CDCl_3$

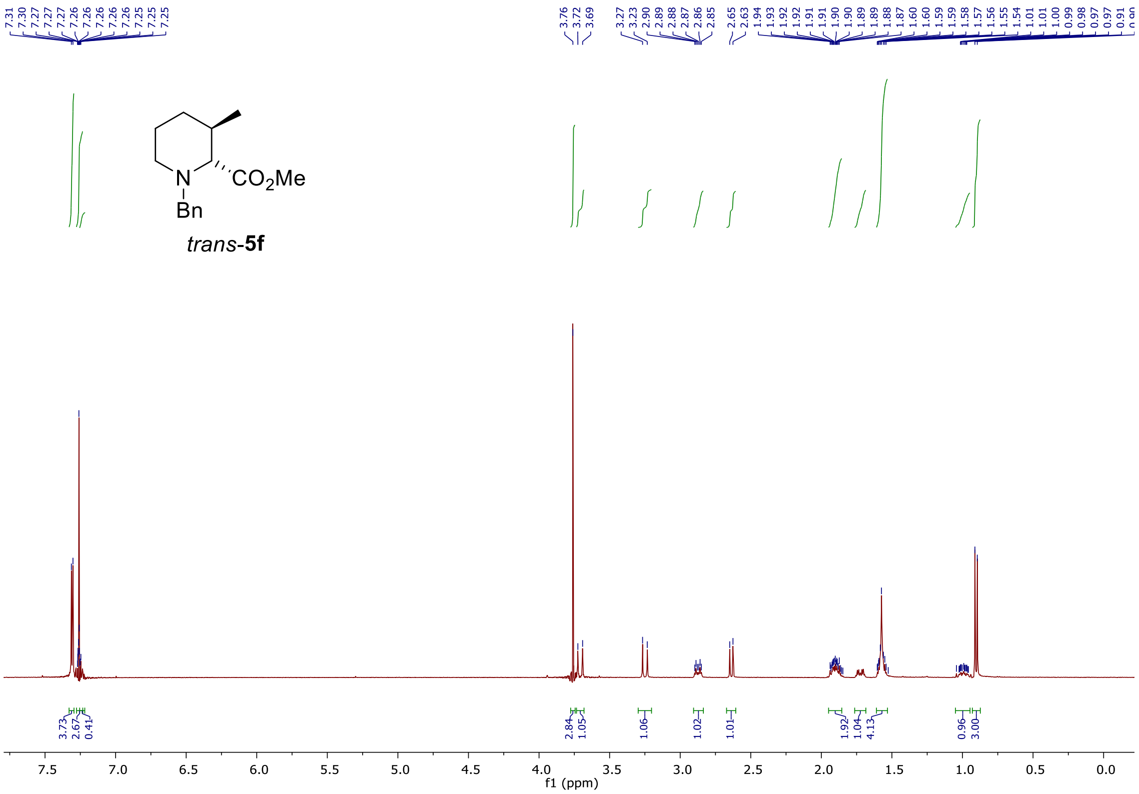

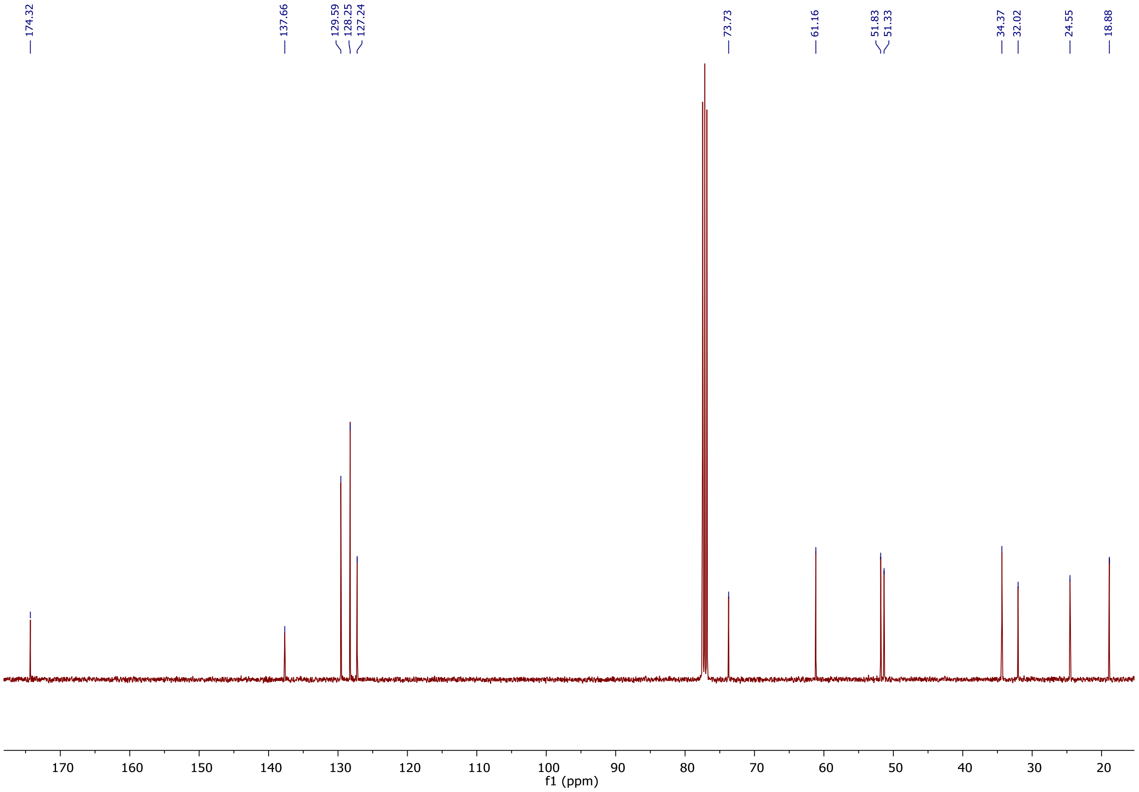

400 MHz ^{1}H NMR spectrum; 100.6 MHz ^{13}C NMR spectrum; $CDCl_3$

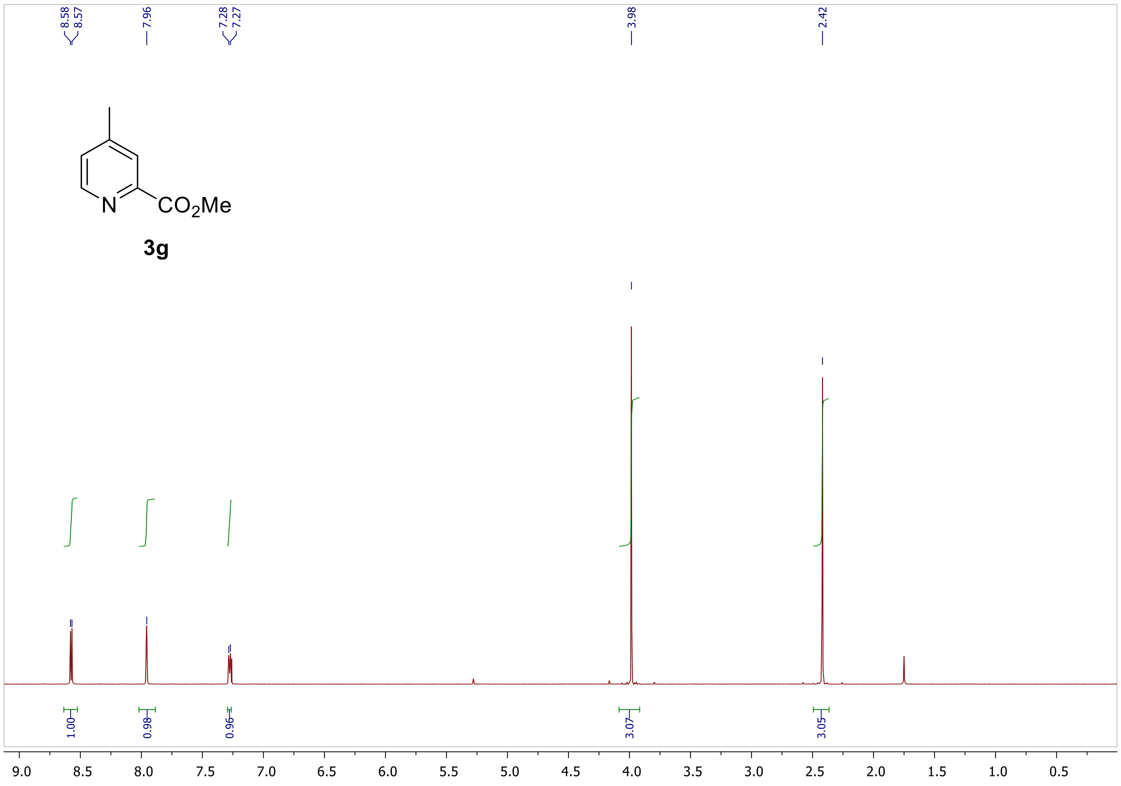

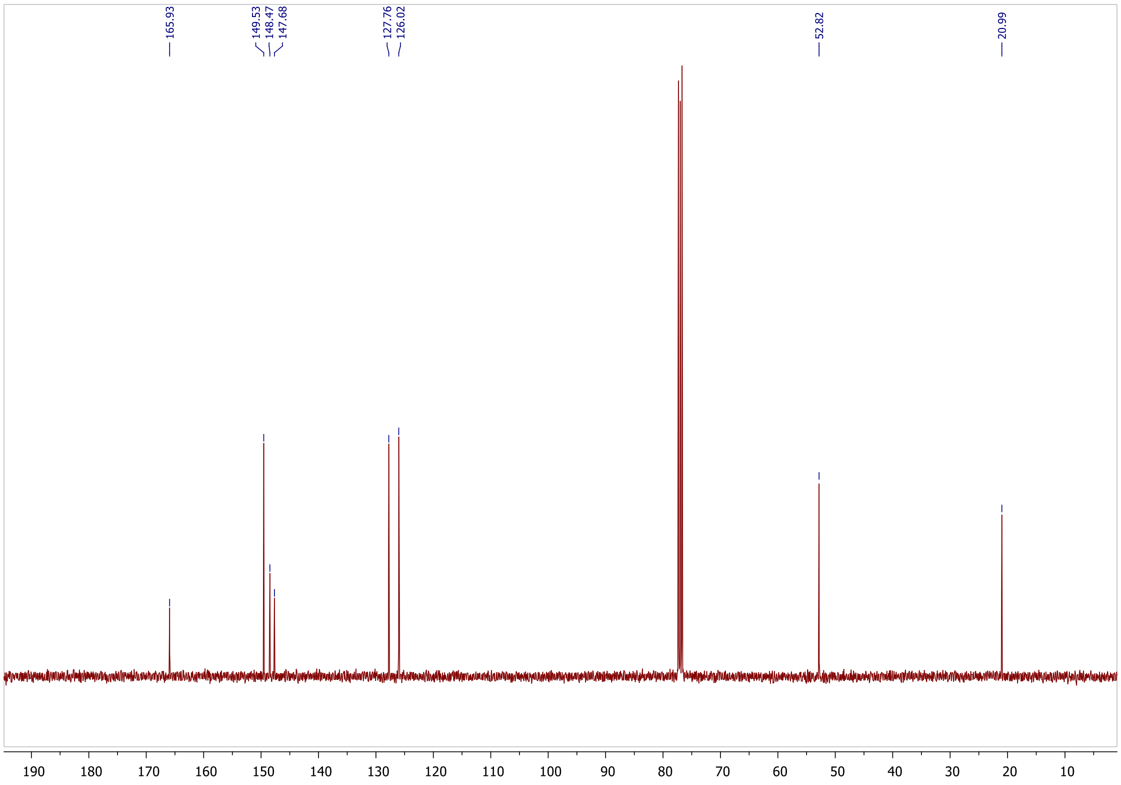

400 MHz ^{1}H NMR spectrum; 100.6 MHz ^{13}C NMR spectrum; $CDCl_3$

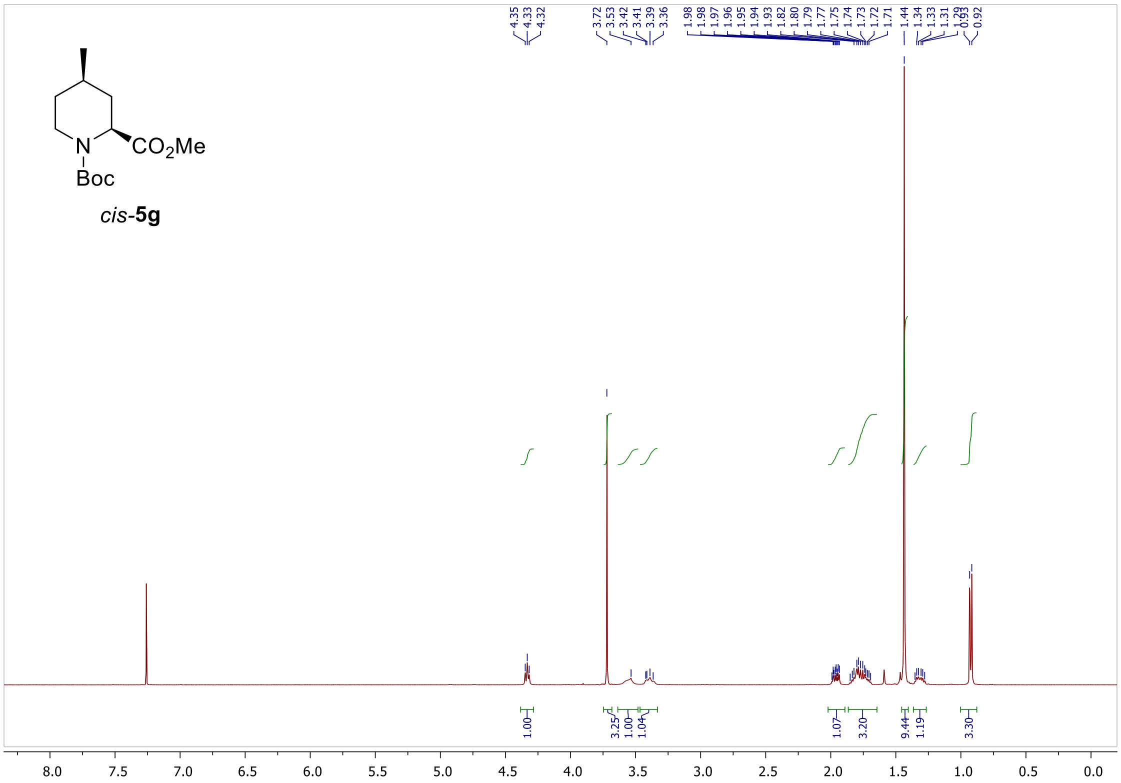

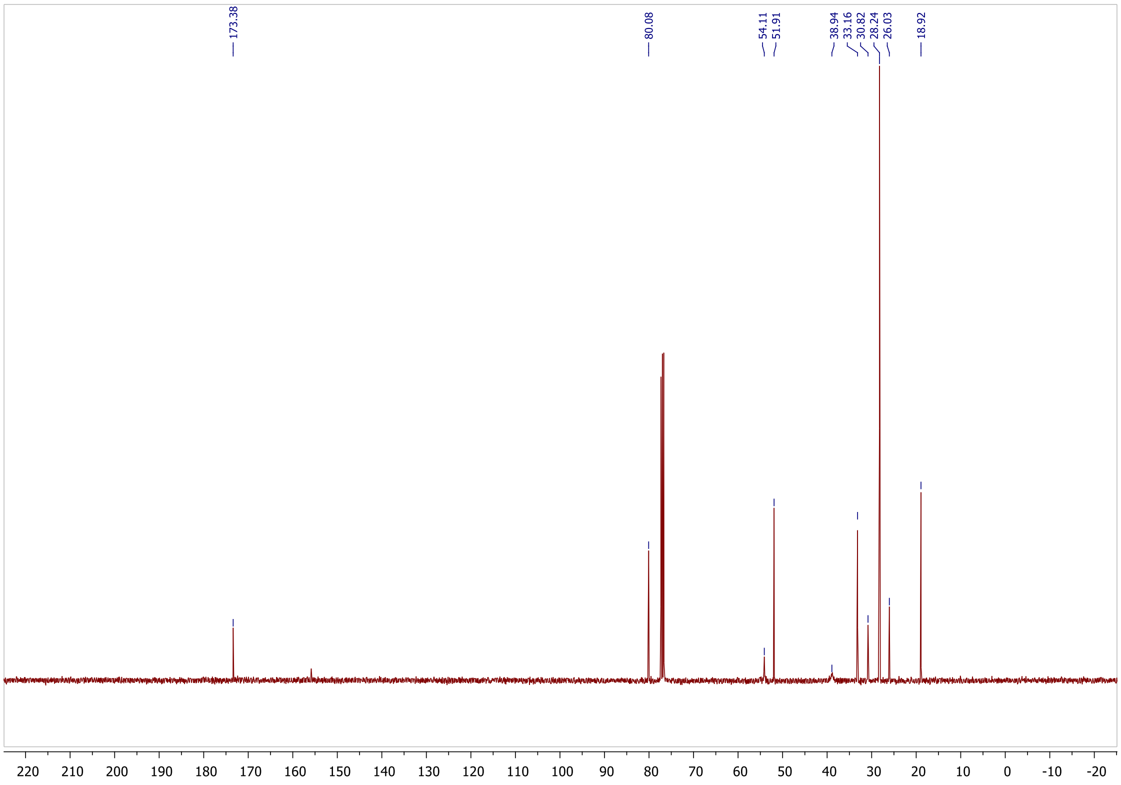

400 MHz ^{1}H NMR spectrum; 100.6 MHz ^{13}C NMR spectrum; $CDCl_3$

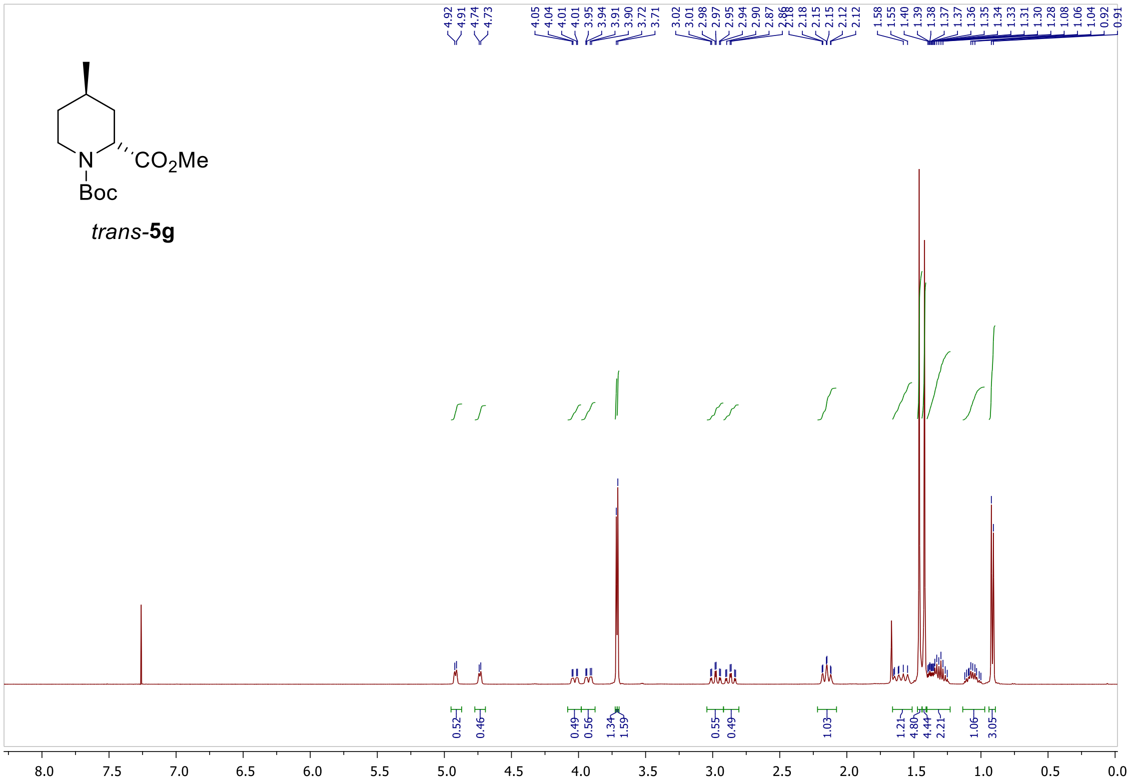

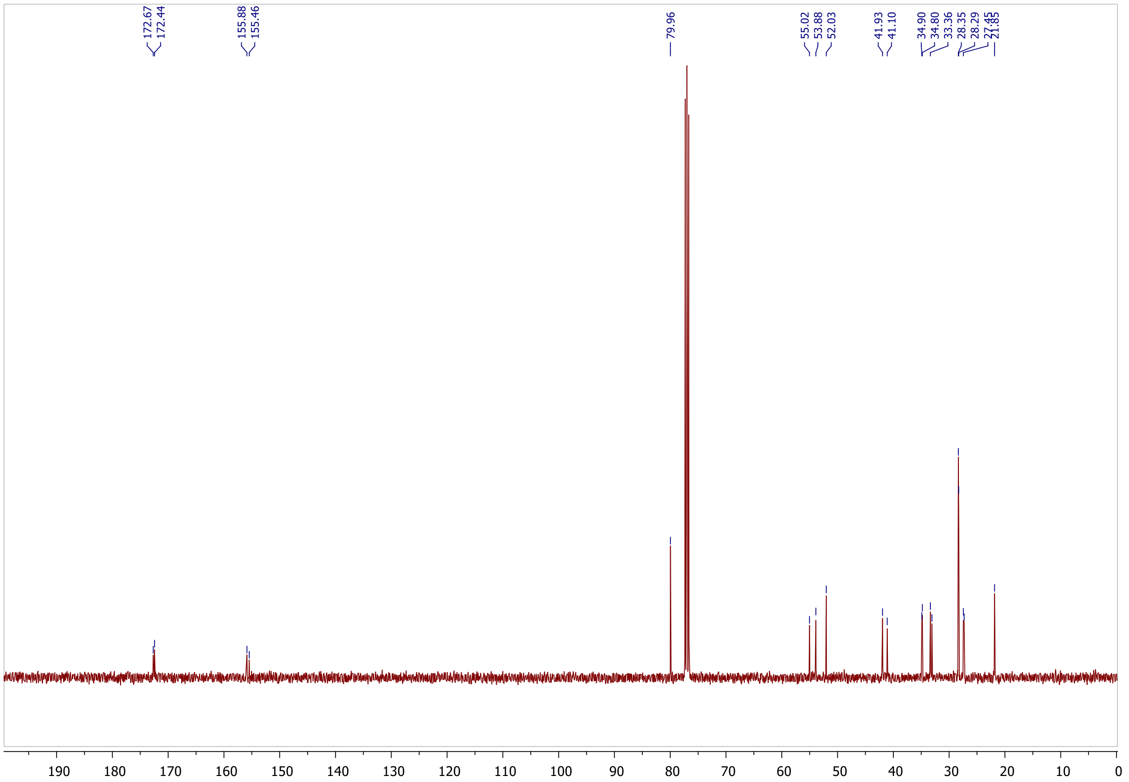

400 MHz ^{1}H NMR spectrum; 100.6 MHz ^{13}C NMR spectrum; MeOH-d_4

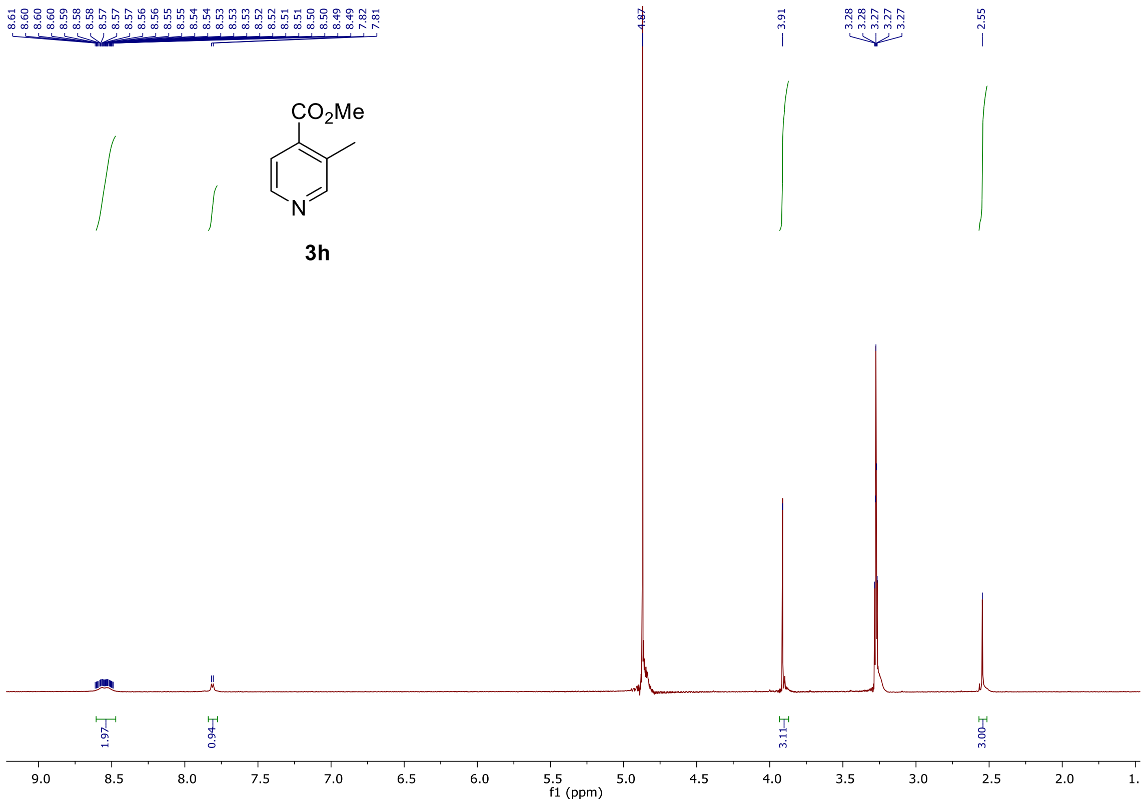

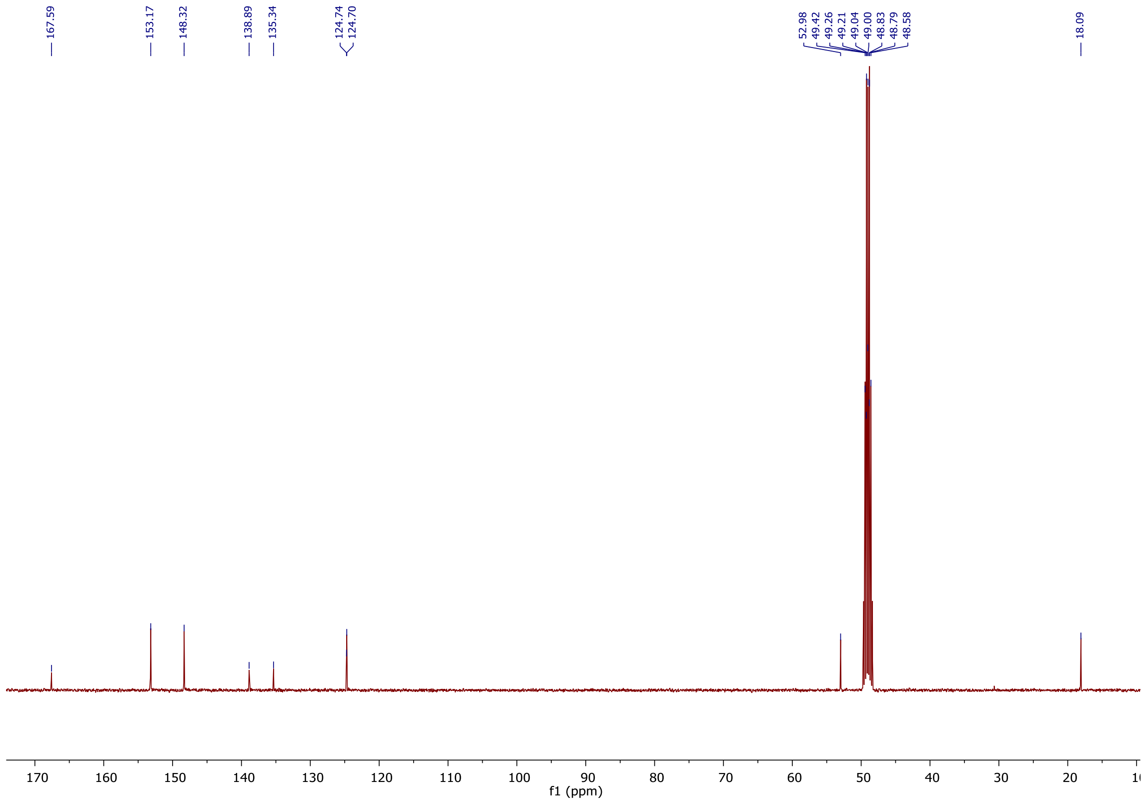

400 MHz ^{1}H NMR spectrum; 100.6 MHz ^{13}C NMR spectrum; $CDCl_3$

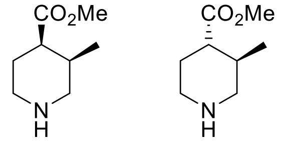

cis-**4h** *trans*-**4h**

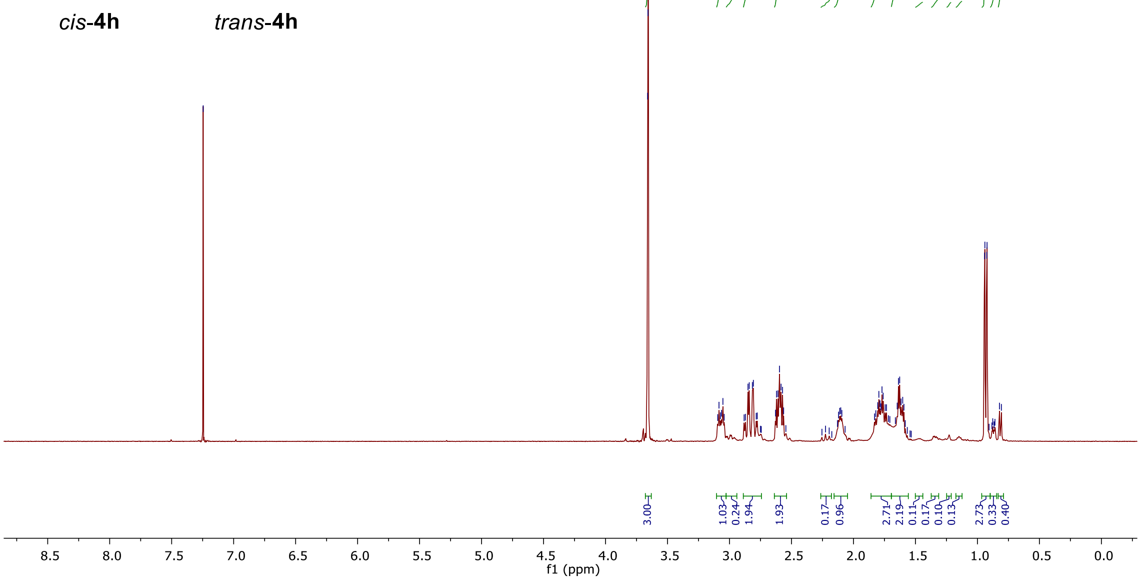

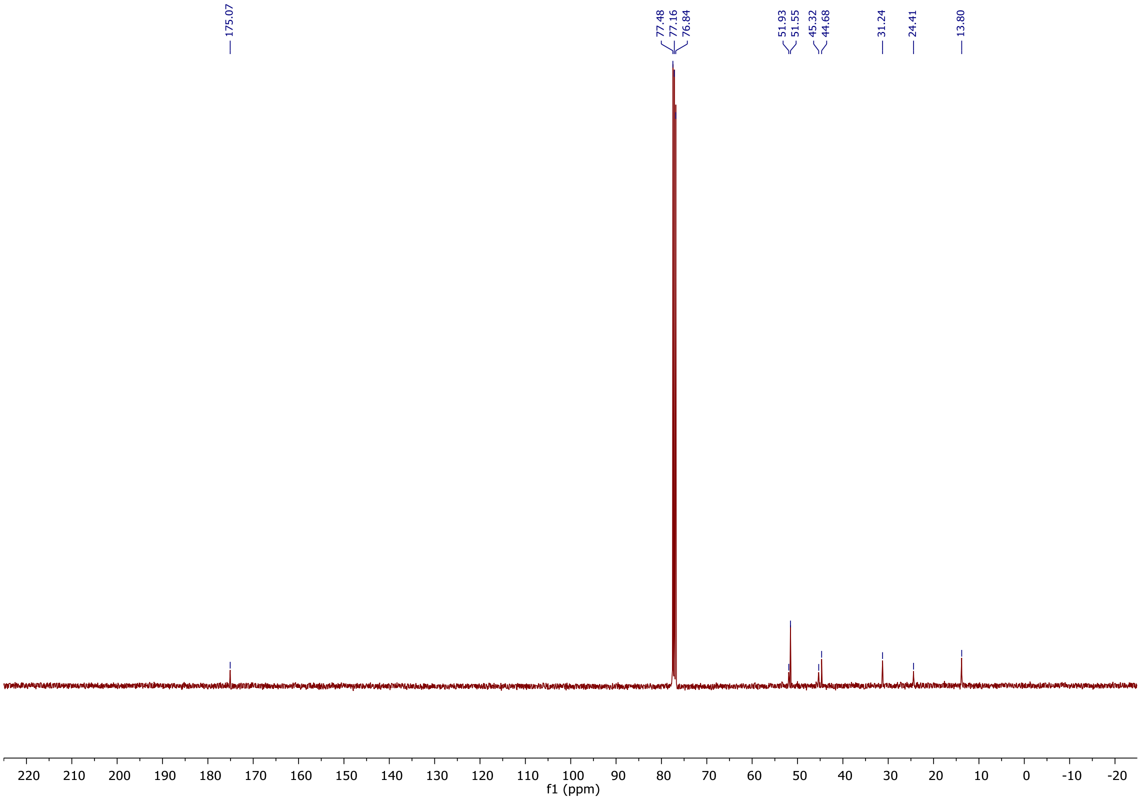

400 MHz ^{1}H NMR spectrum; 100.6 MHz ^{13}C NMR spectrum; $CDCl_3$

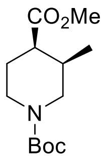

cis-**5h**

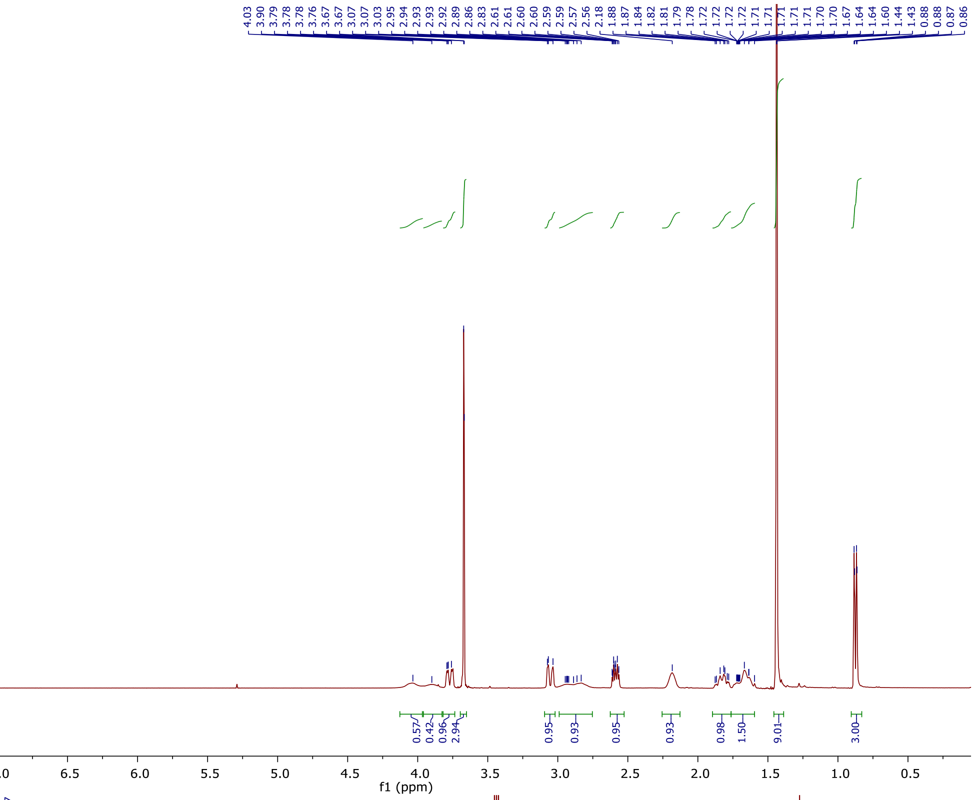

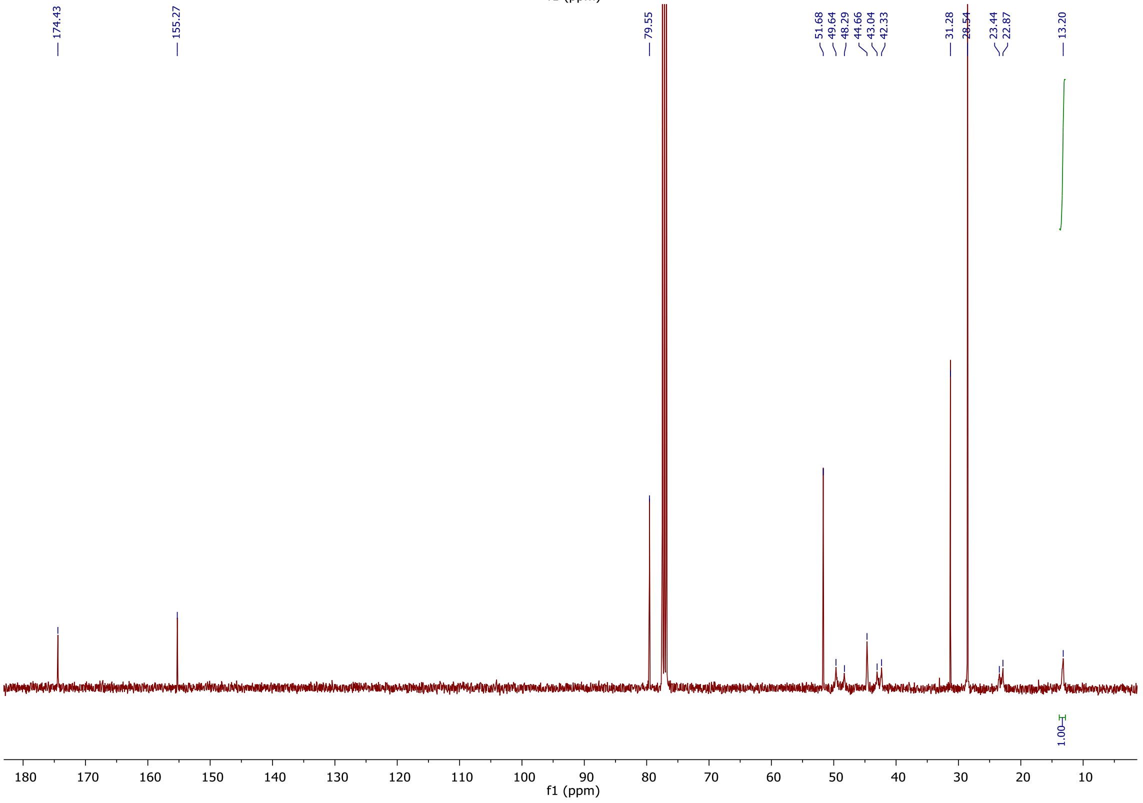

400 MHz ^{1}H NMR spectrum; 100.6 MHz ^{13}C NMR spectrum; $CDCl_3$

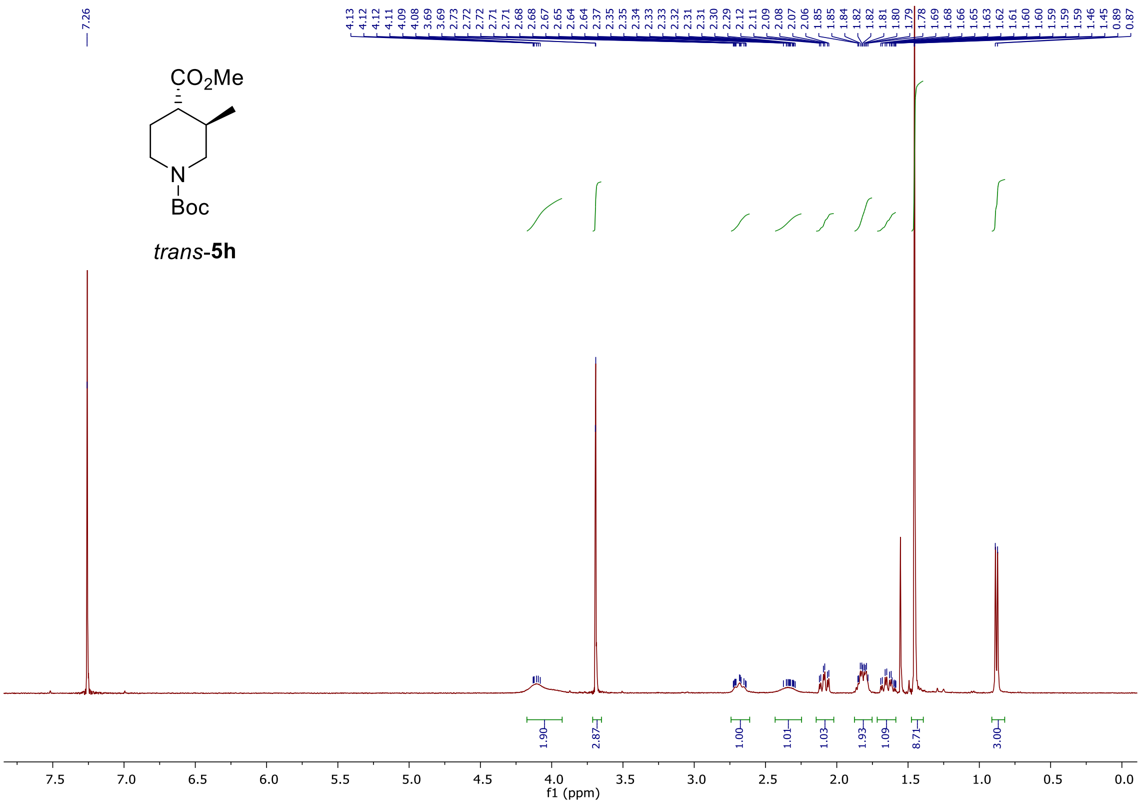

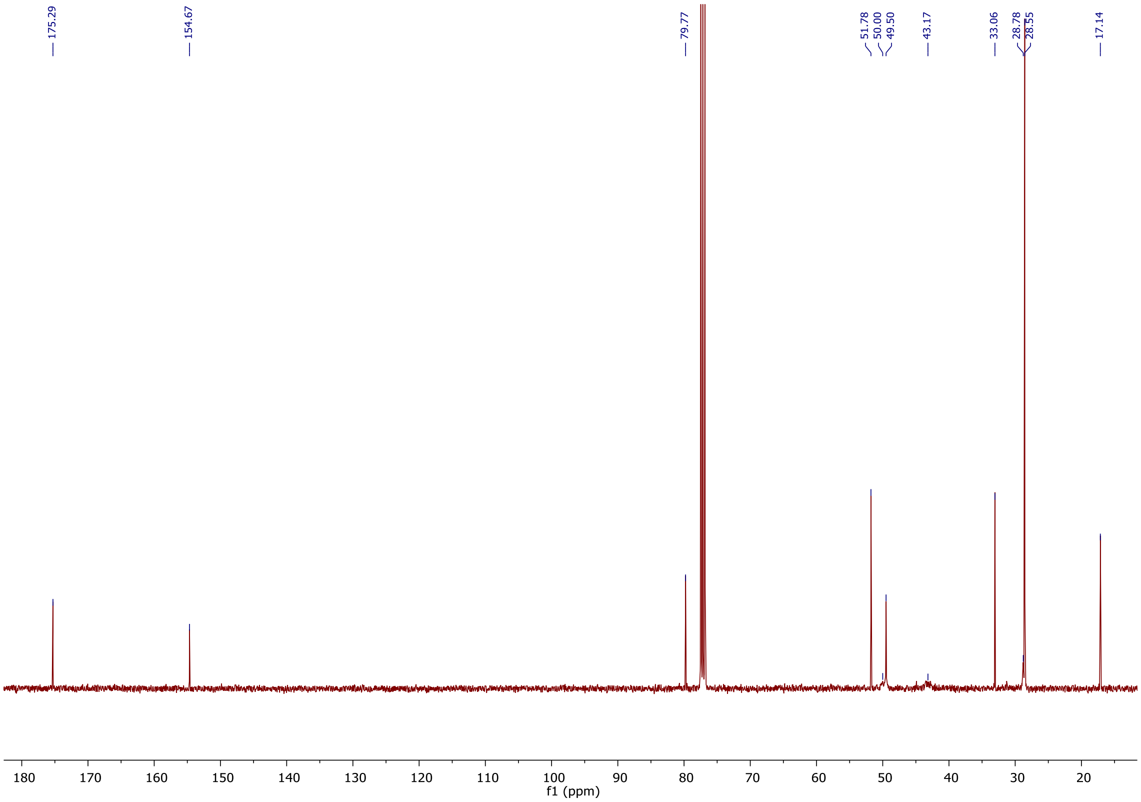

400 MHz ^{1}H NMR spectrum; 100.6 MHz ^{13}C NMR spectrum; $CDCl_3$

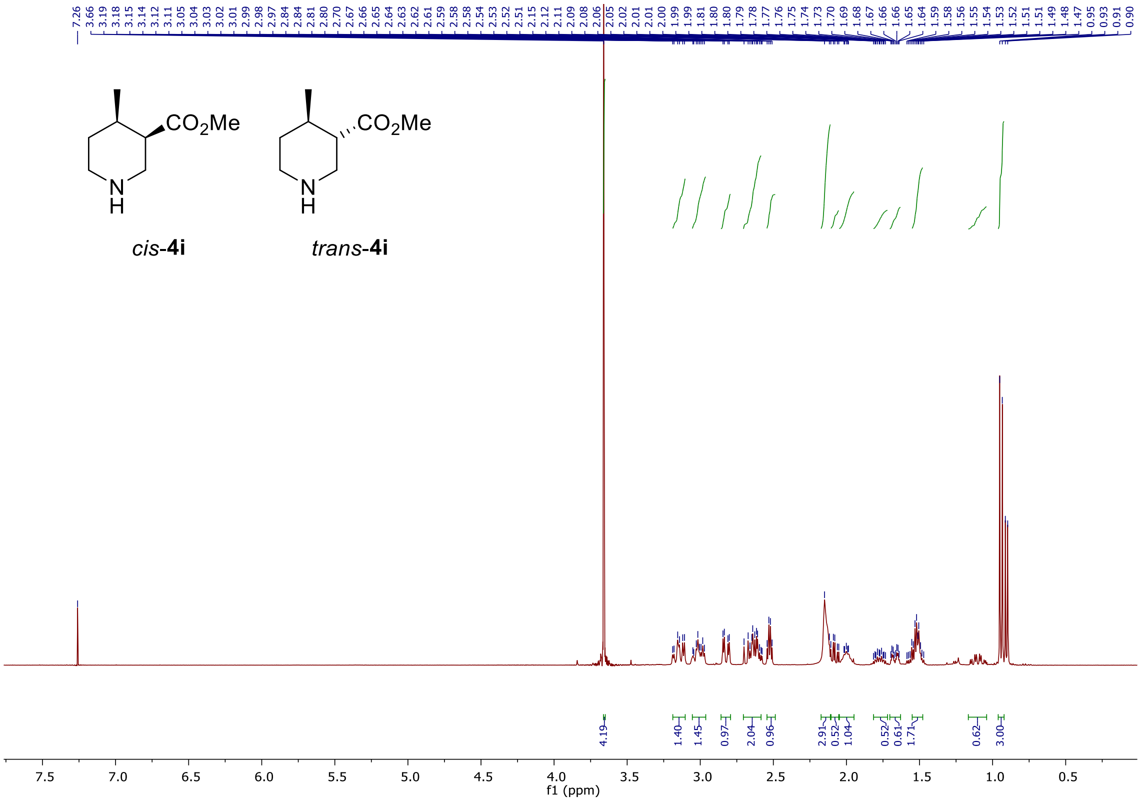

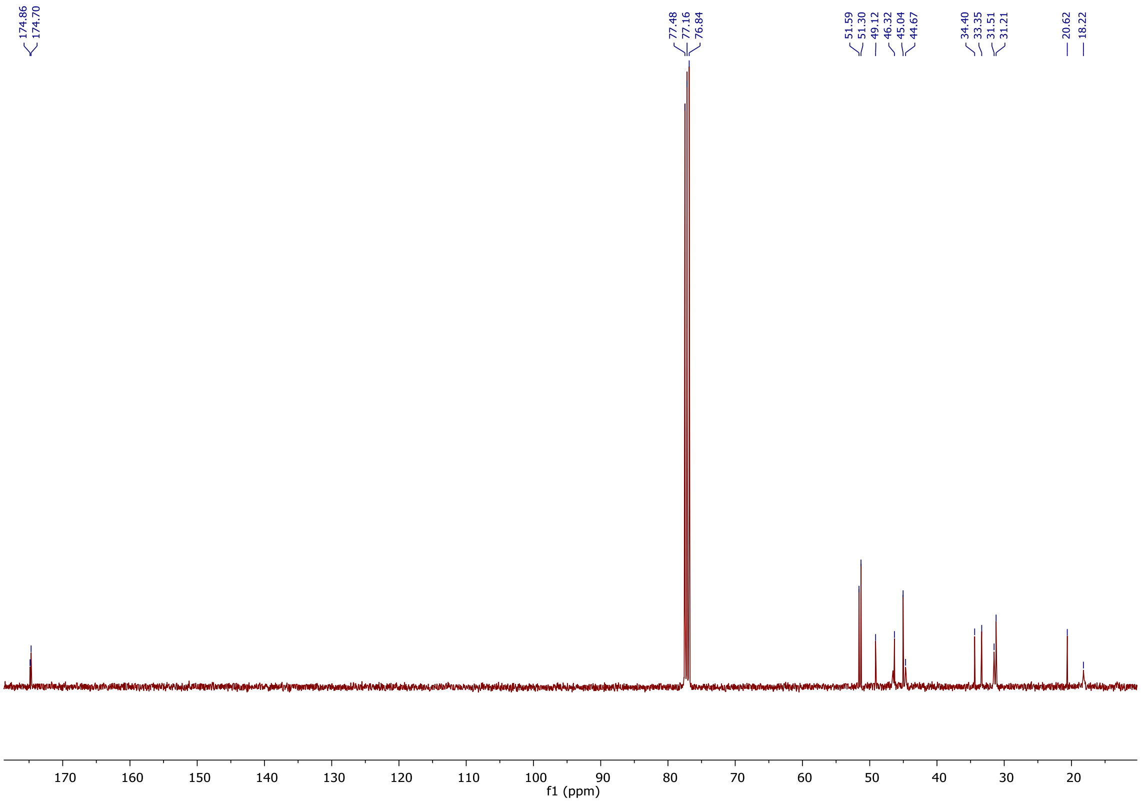

400 MHz ^1H NMR spectrum; 100.6 MHz ^{13}C NMR spectrum; $CDCl_3$

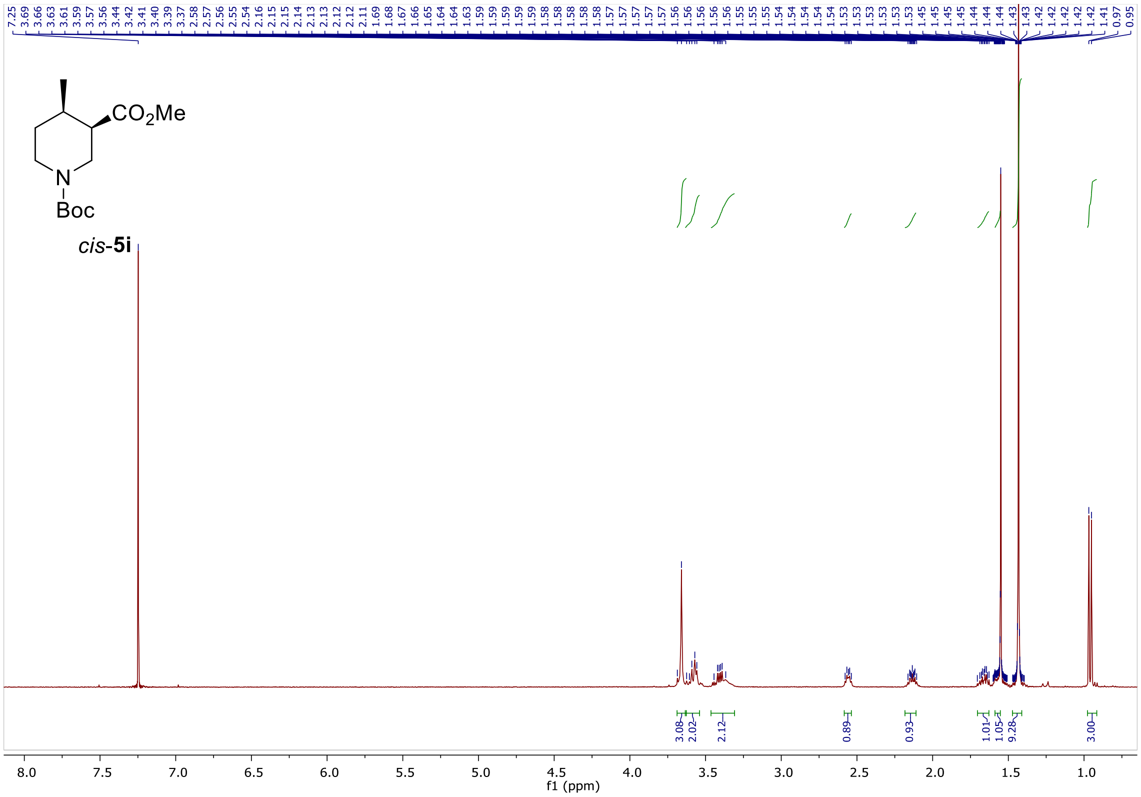

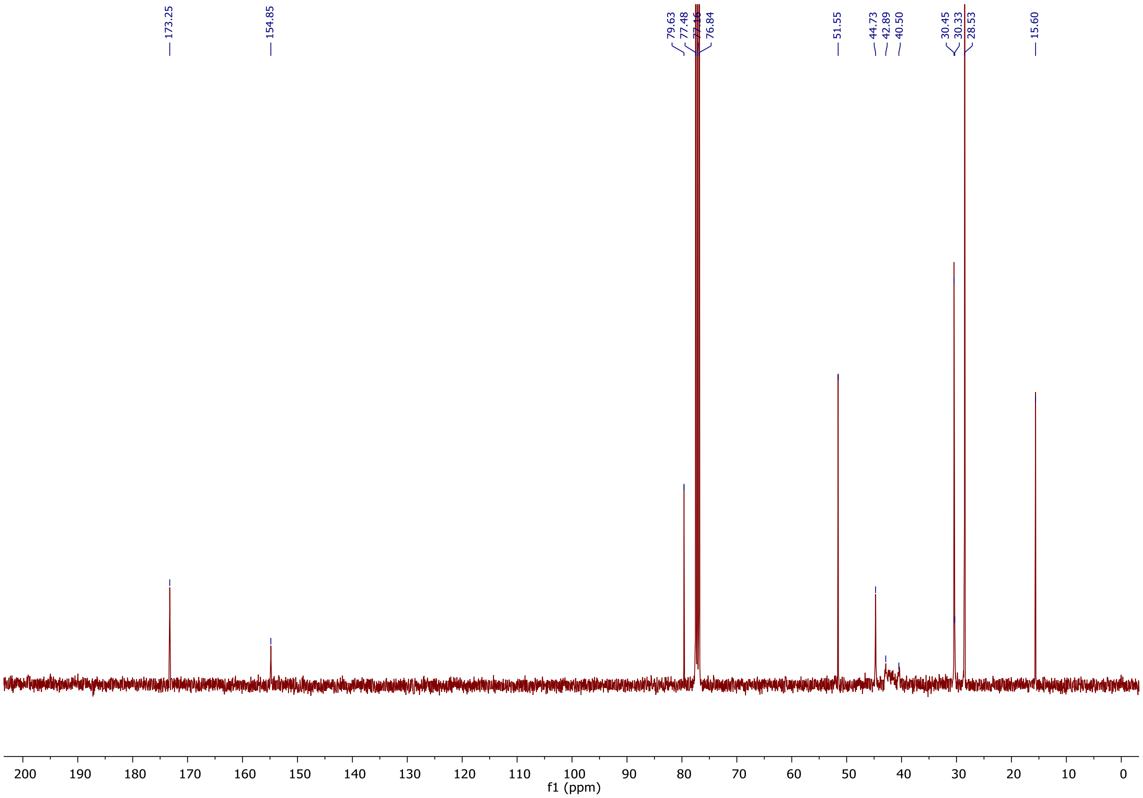

400 MHz ^{1}H NMR spectrum; 100.6 MHz ^{13}C NMR spectrum; $CDCl_3$

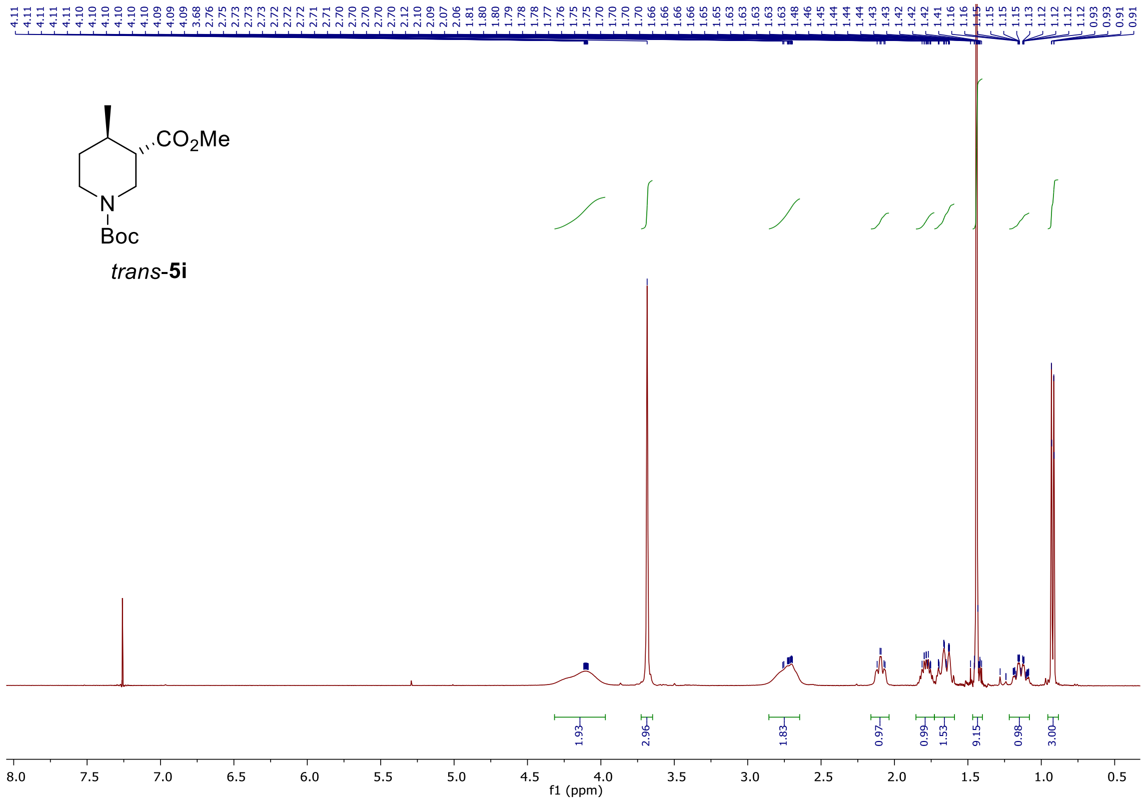

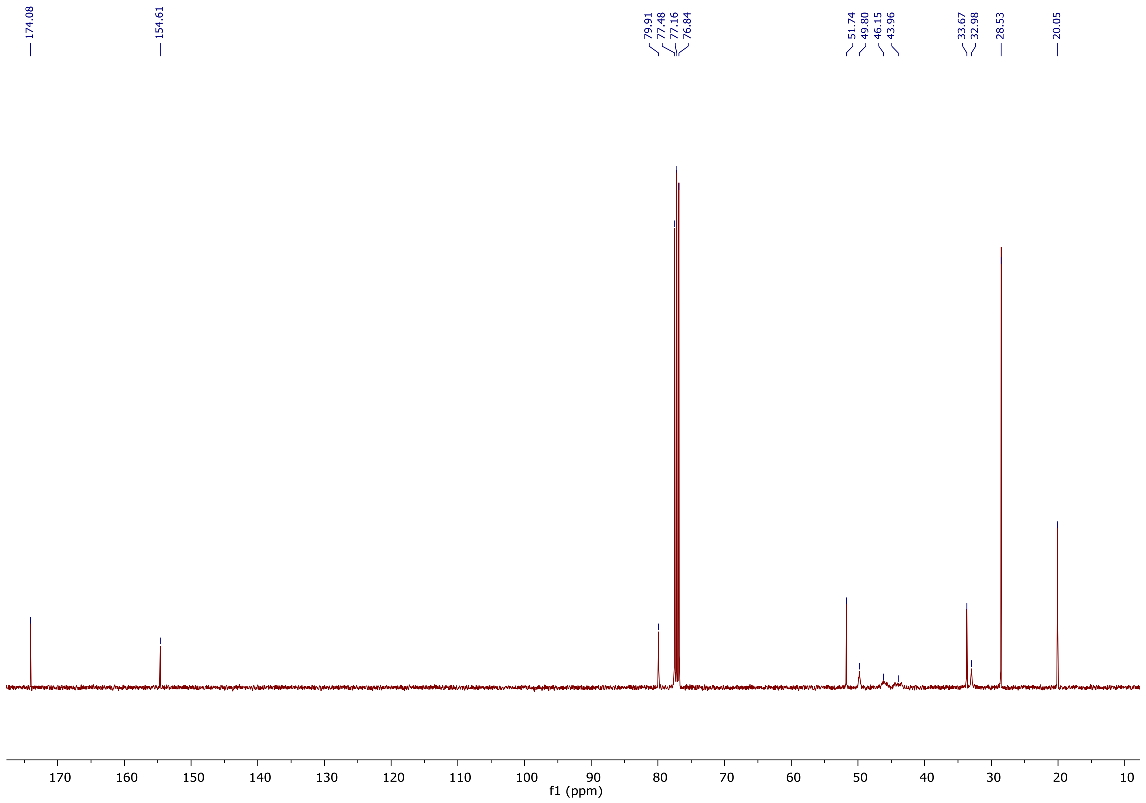

400 MHz ^{1}H NMR spectrum; $CDCl_3$ 100.6 MHz ^{13}C NMR spectrum; MeOH-d_4

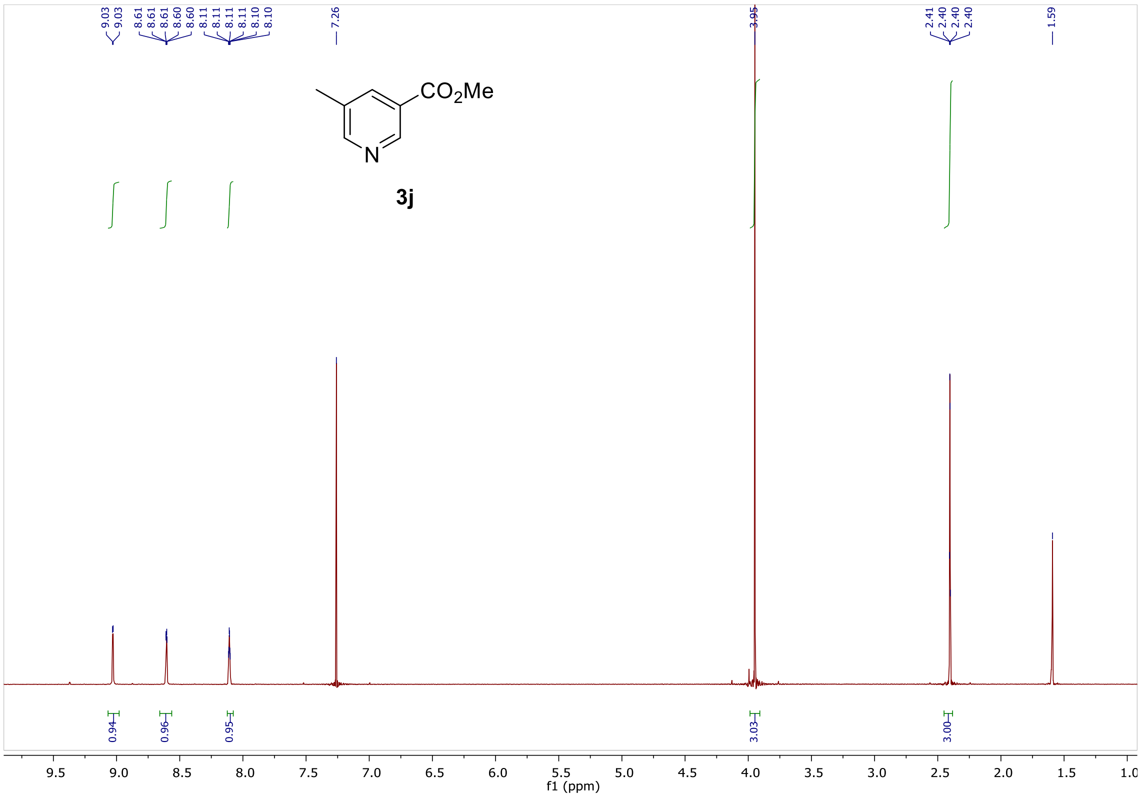

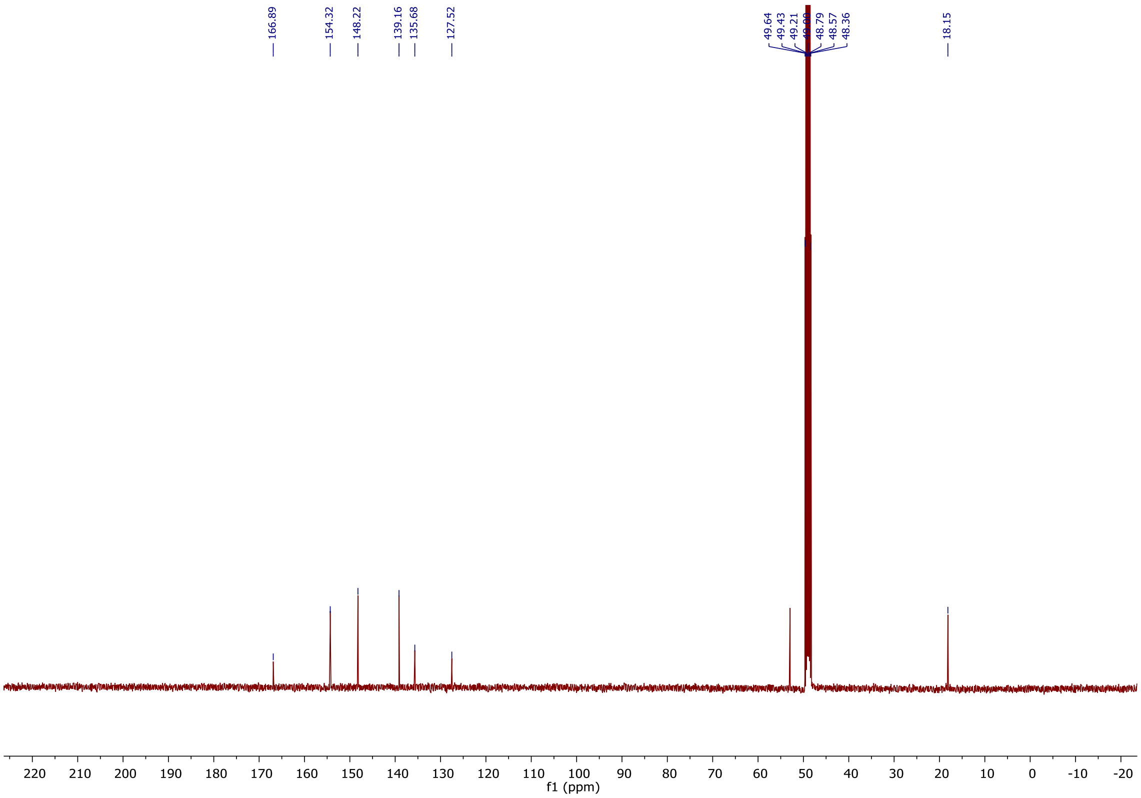

400 MHz ^1H NMR spectrum; 100.6 MHz ^{13}C NMR spectrum; $CDCl_3$

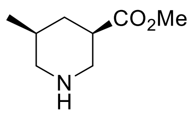

cis-**4j**

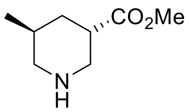

trans-**4j**

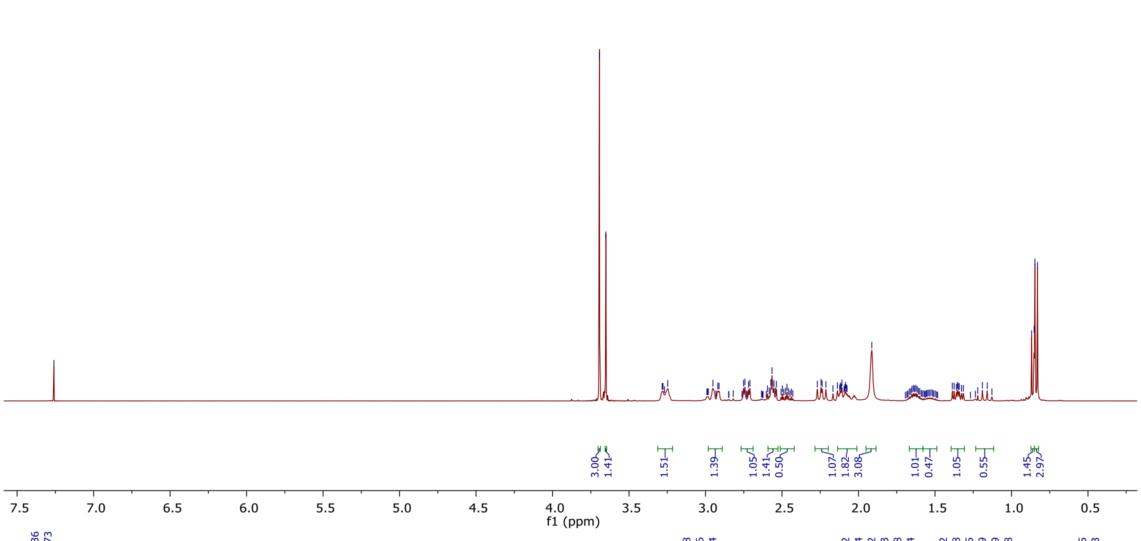

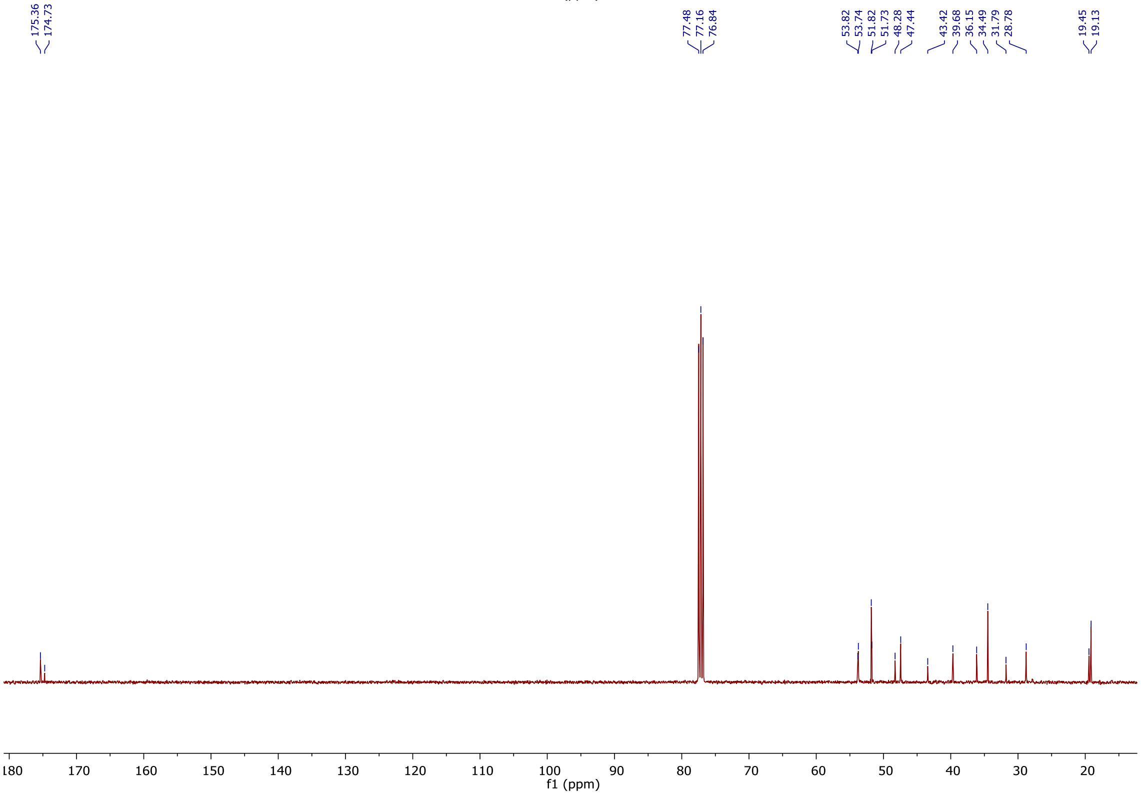

400 MHz ^{1}H NMR spectrum; 100.6 MHz ^{13}C NMR spectrum; $CDCl_3$

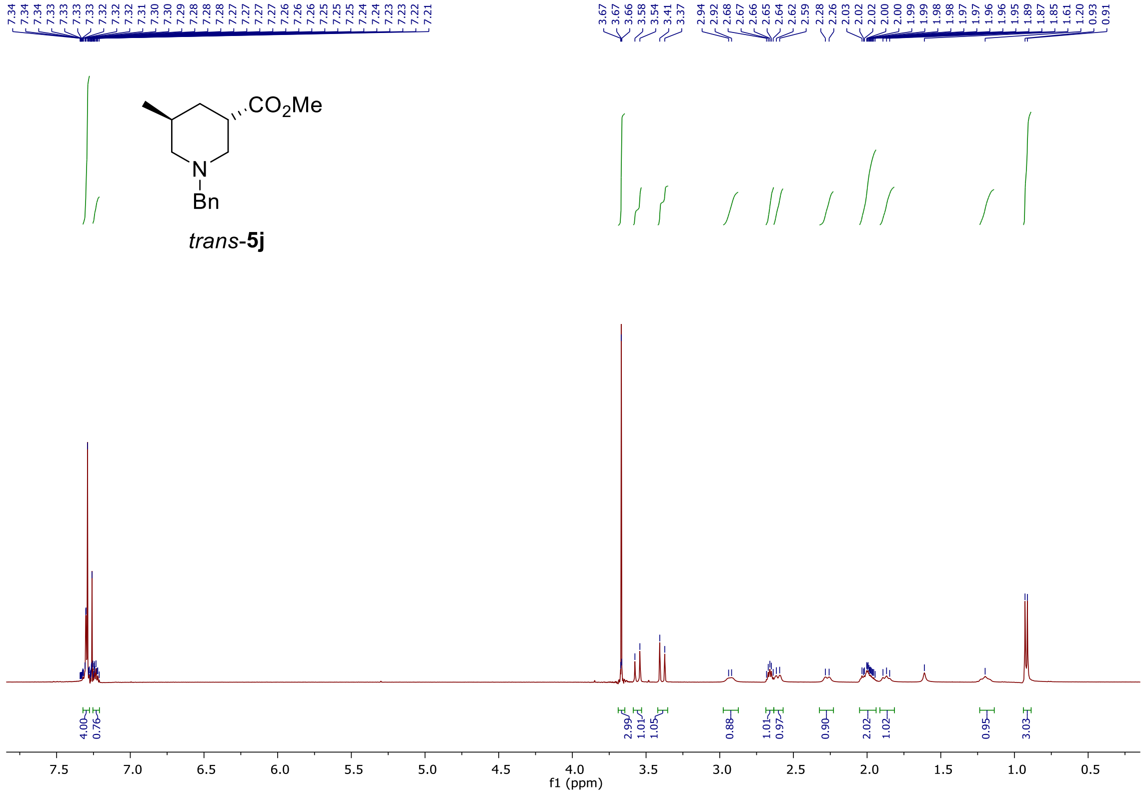

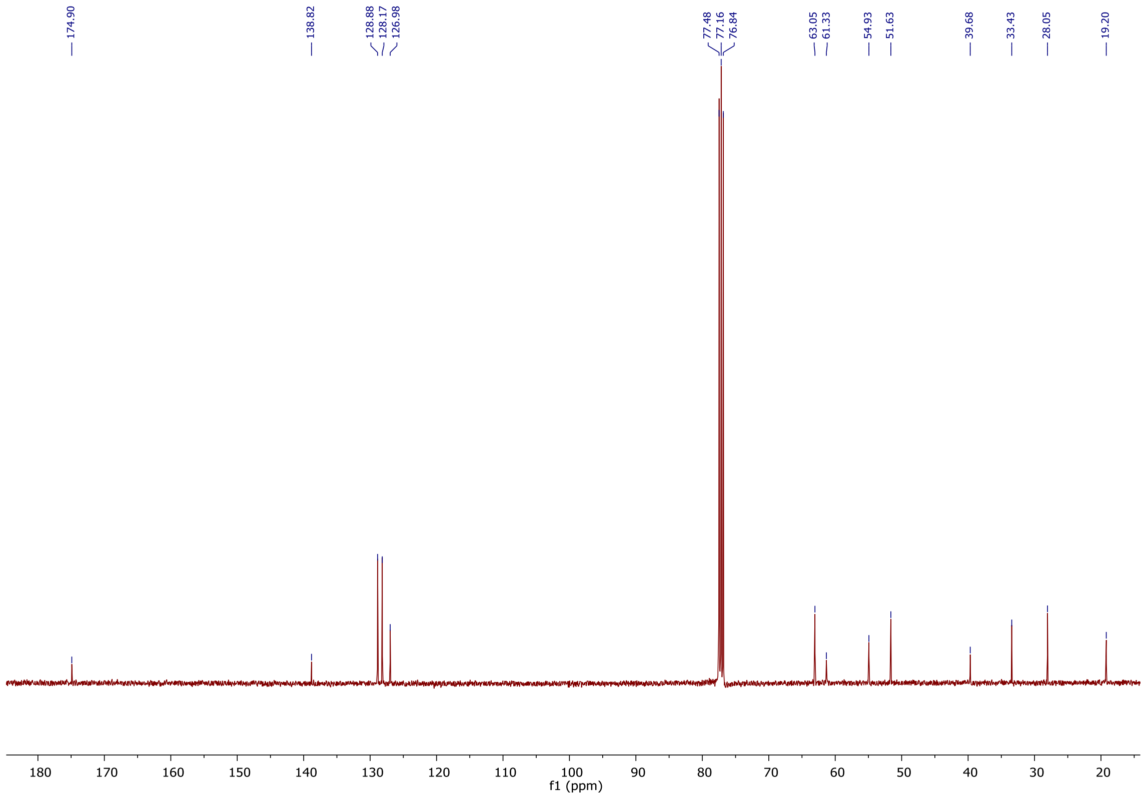

400 MHz ^{1}H NMR spectrum; 100.6 MHz ^{13}C NMR spectrum; $CDCl_3$

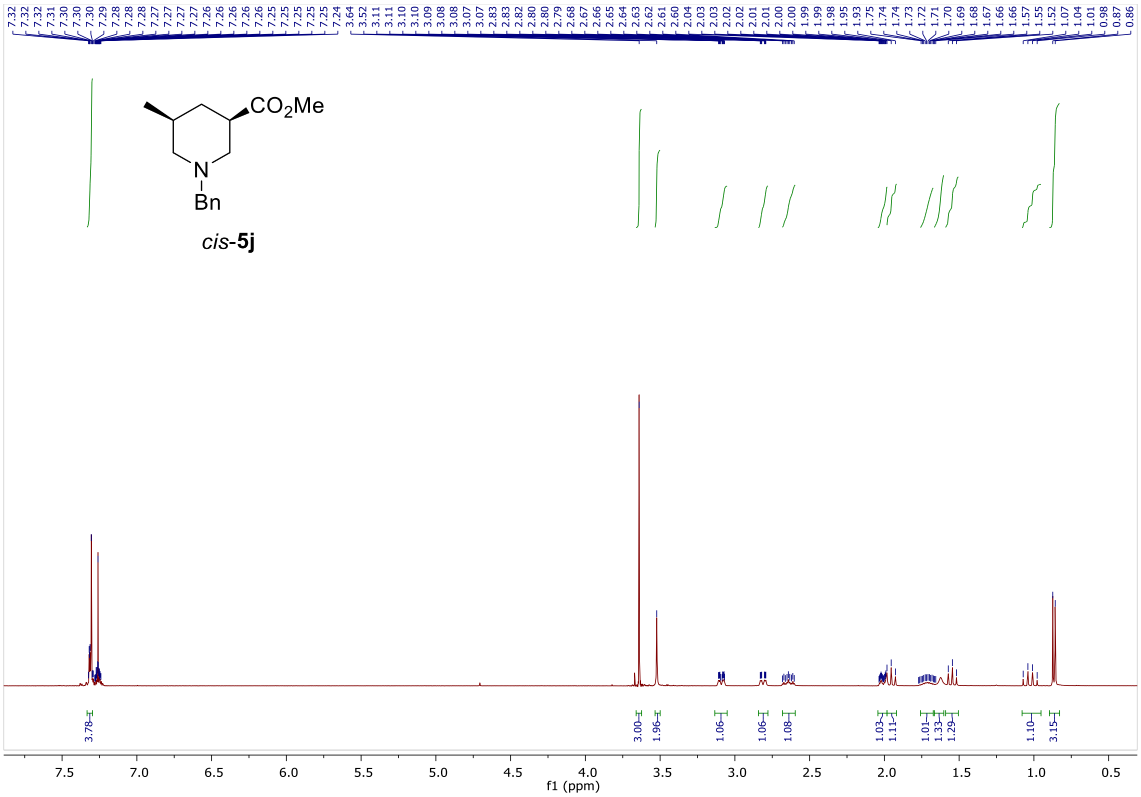

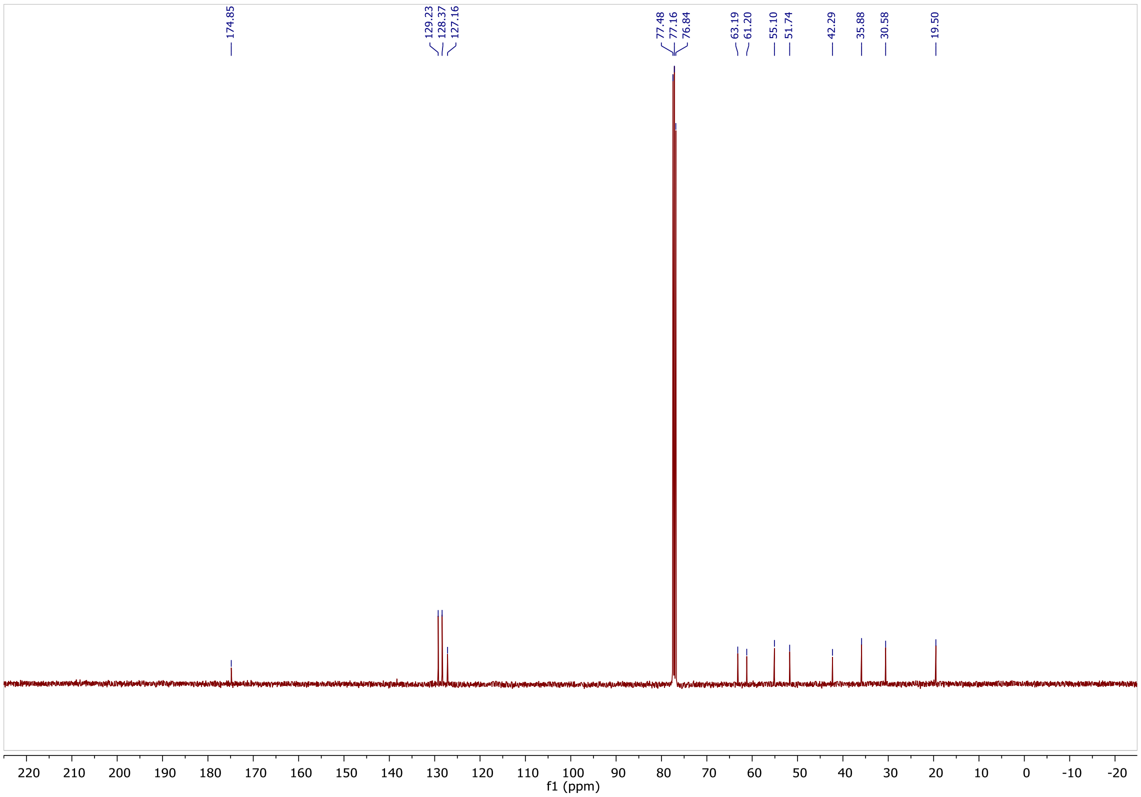

400 MHz ^{1}H NMR spectrum; 100.6 MHz ^{13}C NMR spectrum; $CDCl_3$

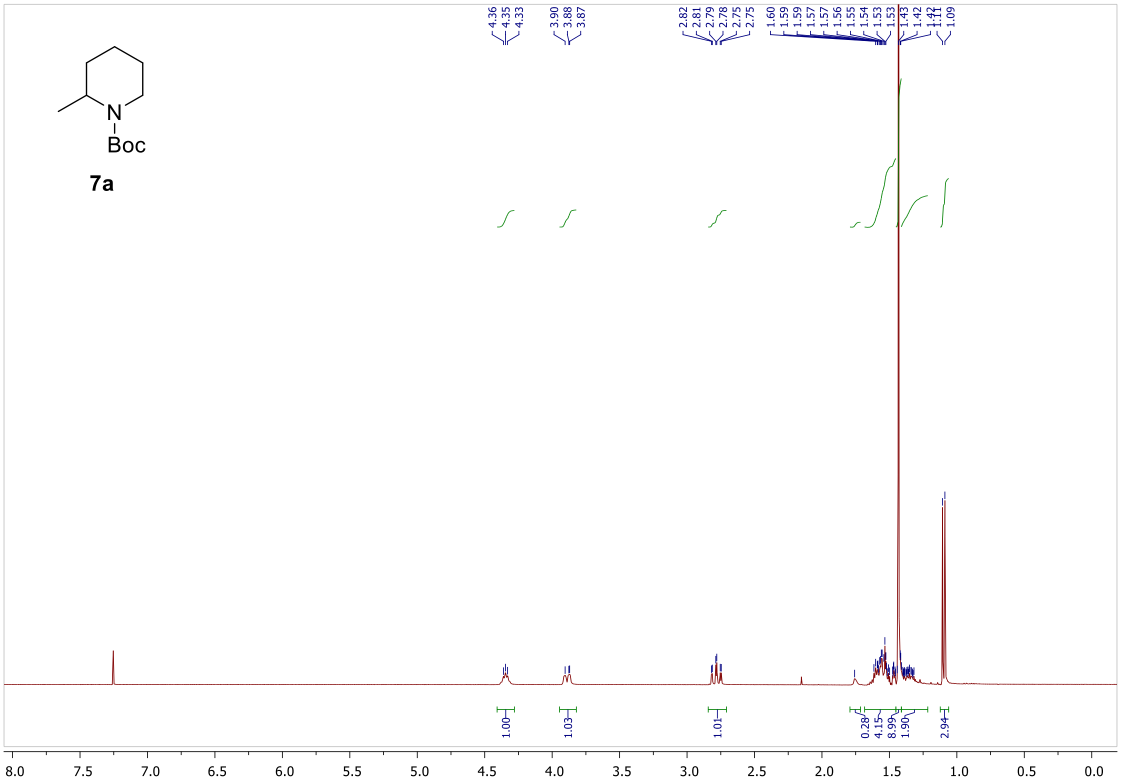

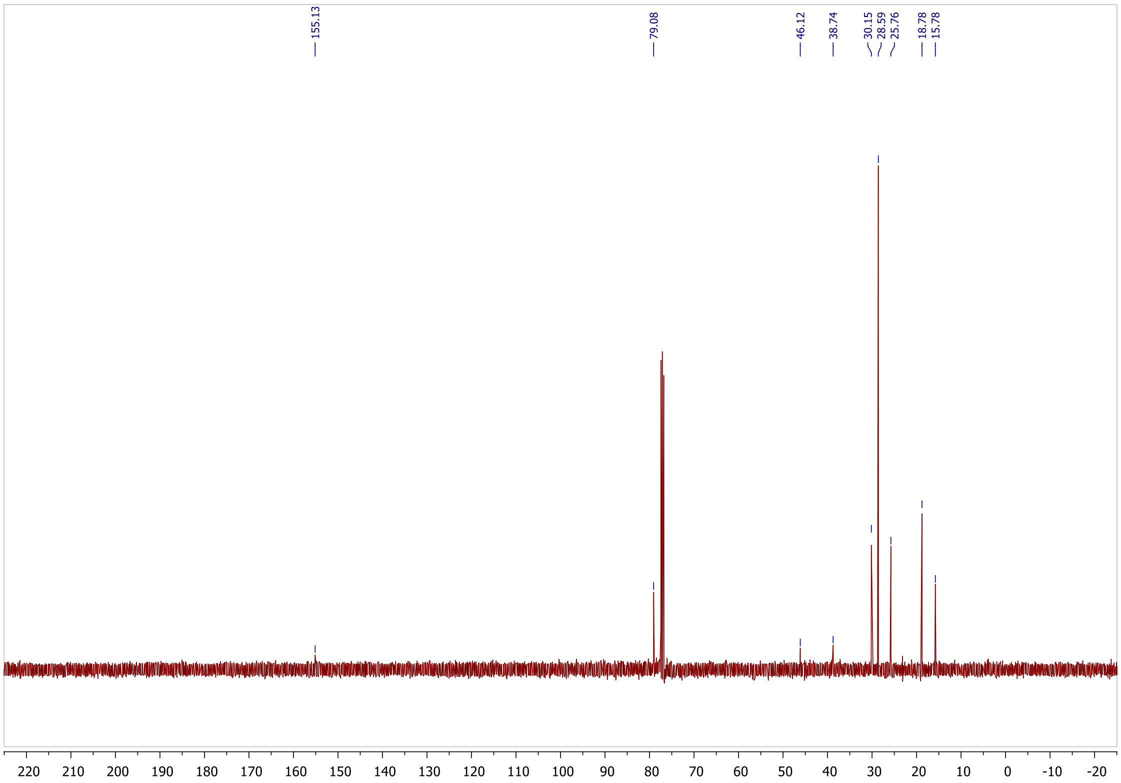

400 MHz ^{1}H NMR spectrum; 100.6 MHz ^{13}C NMR spectrum; $CDCl_3$

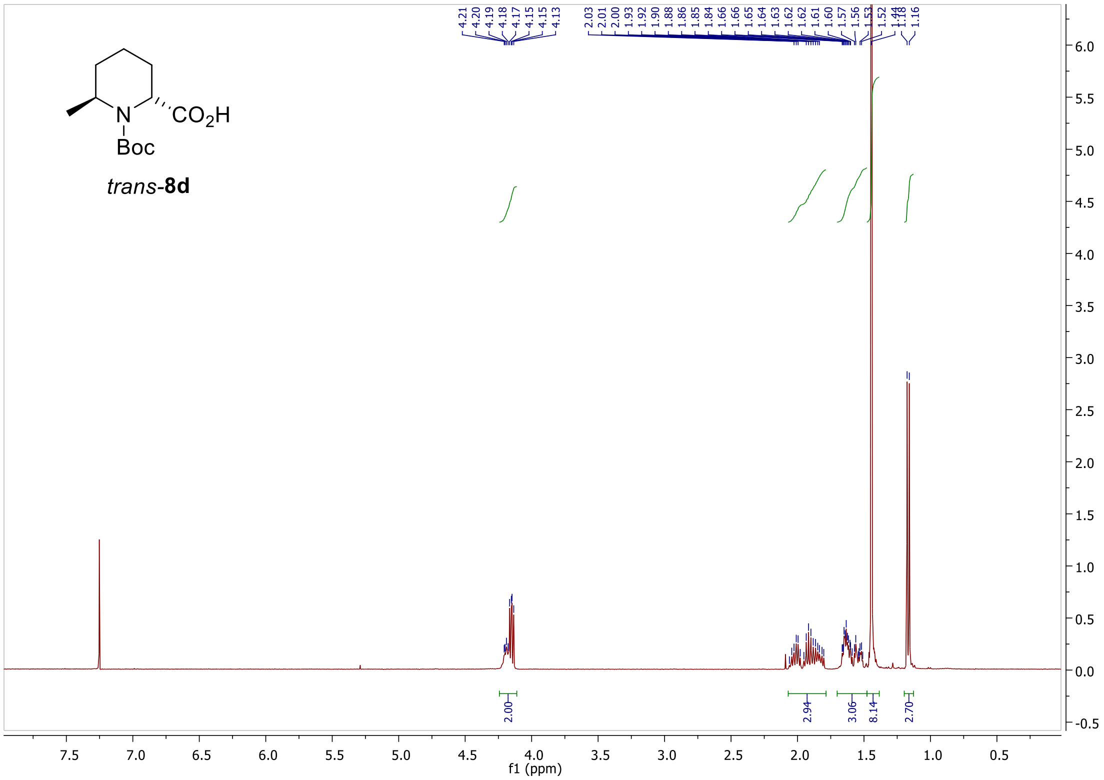
N
CO2H
Boc
trans-8d
4.21
4.20
4.19
4.18
4.17
4.15
4.15
4.13
2.03
2.01
2.00
1.93
1.92
1.90
1.88
1.86
1.85
1.84
1.66
1.66
1.65
1.64
1.63
1.62
1.62
1.61
1.60
1.57
1.56
1.53
1.52
1.44
1.18
1.16
2.00
2.94
3.06
8.14
2.70
f1 (ppm)

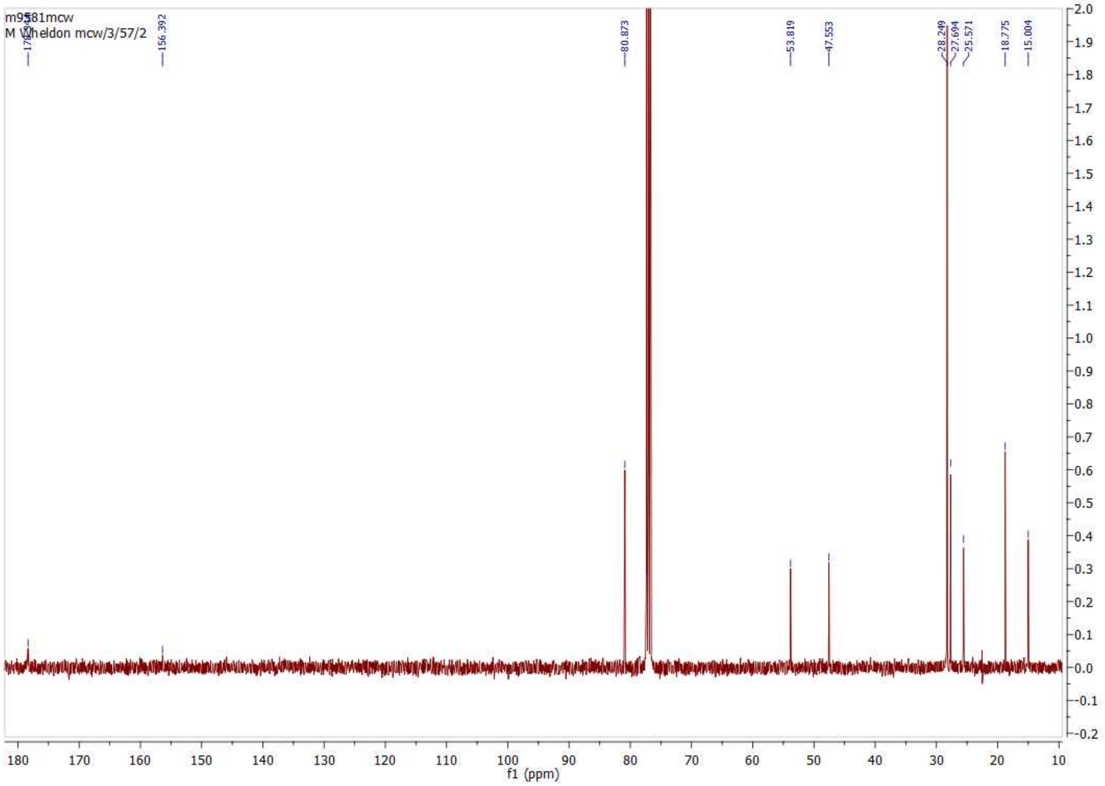
m9581mcw
M Wheldon mcw/3/57/2
156.392
80.873
53.819
47.553
28.249
27.694
25.571
18.775
15.004
f1 (ppm)

400 MHz ^{1}H NMR spectrum; 100.6 MHz ^{13}C NMR spectrum; $CDCl_3$

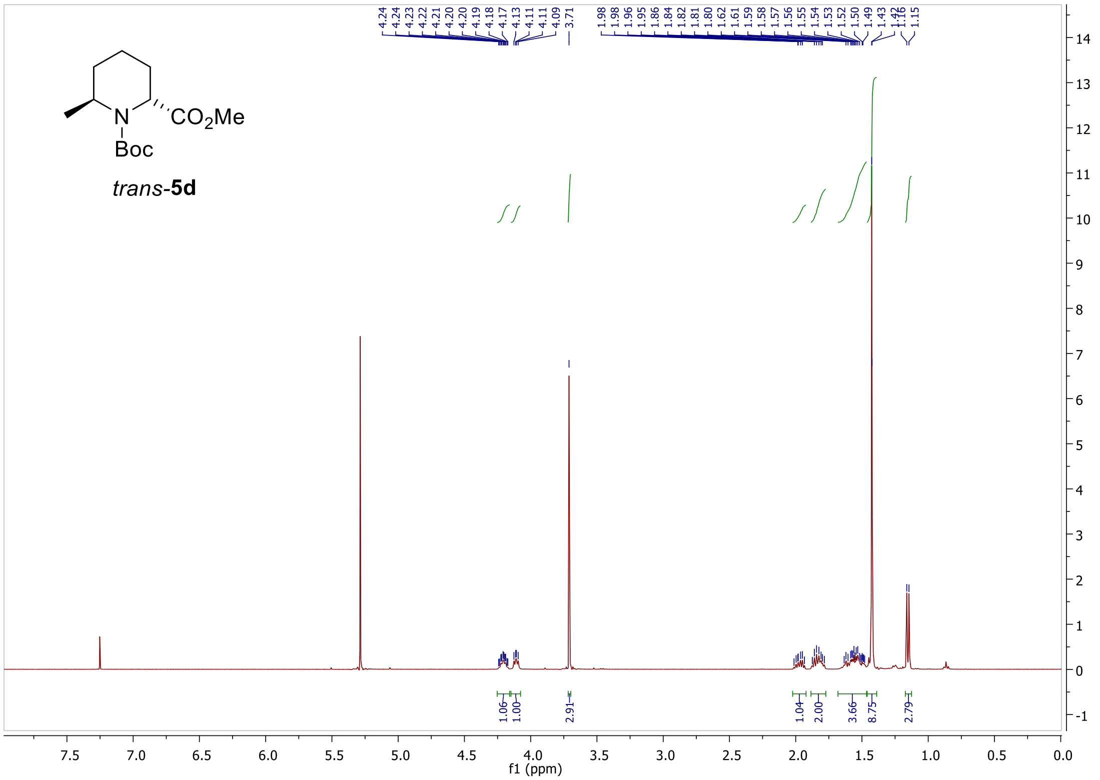

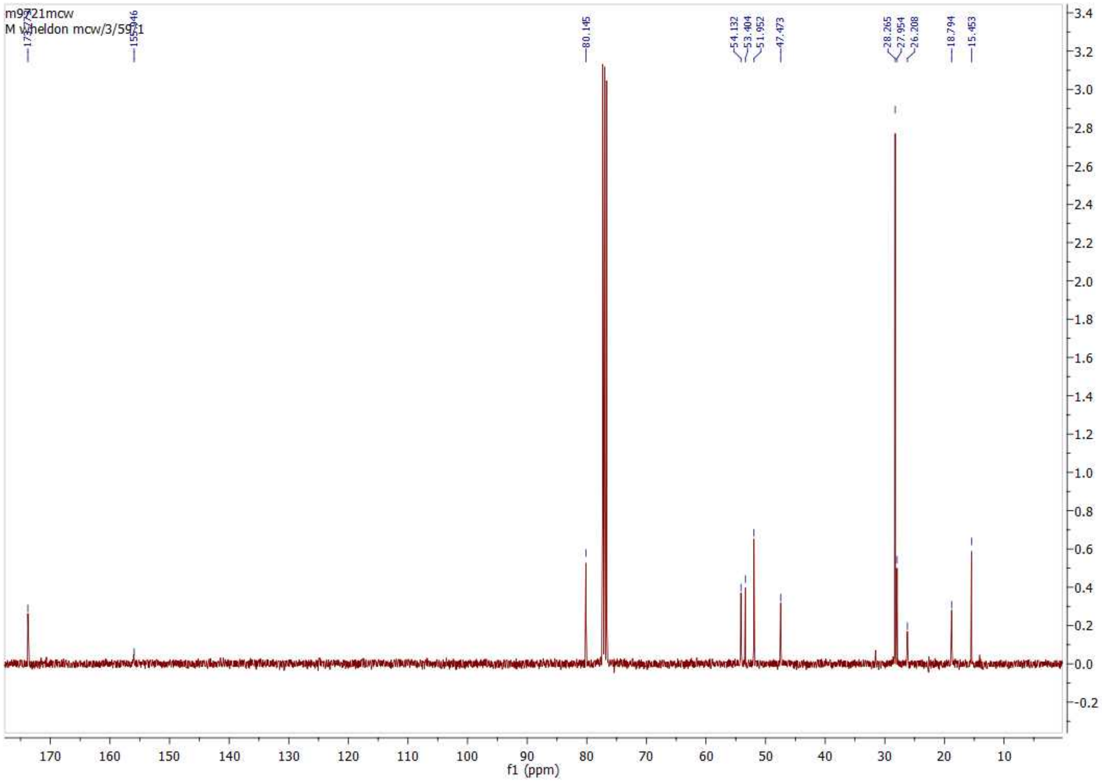

400 MHz ^{1}H NMR spectrum; 100.6 MHz ^{13}C NMR spectrum; $CDCl_3$

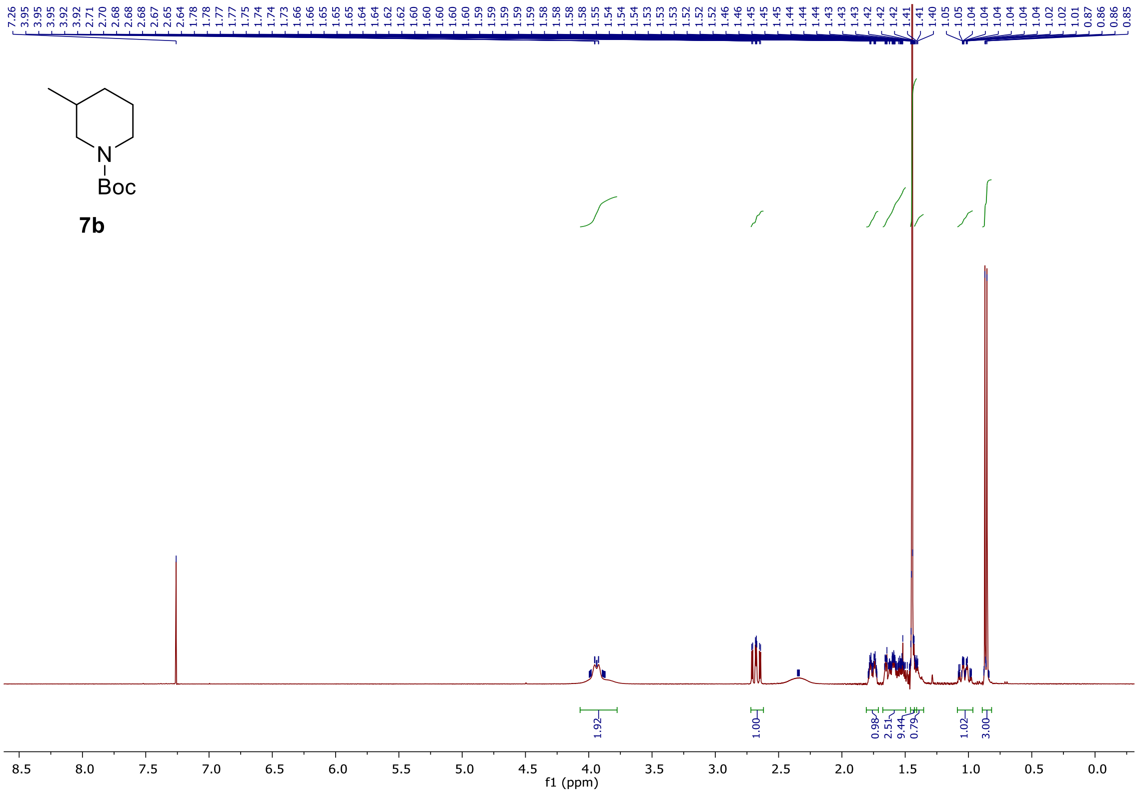

7b

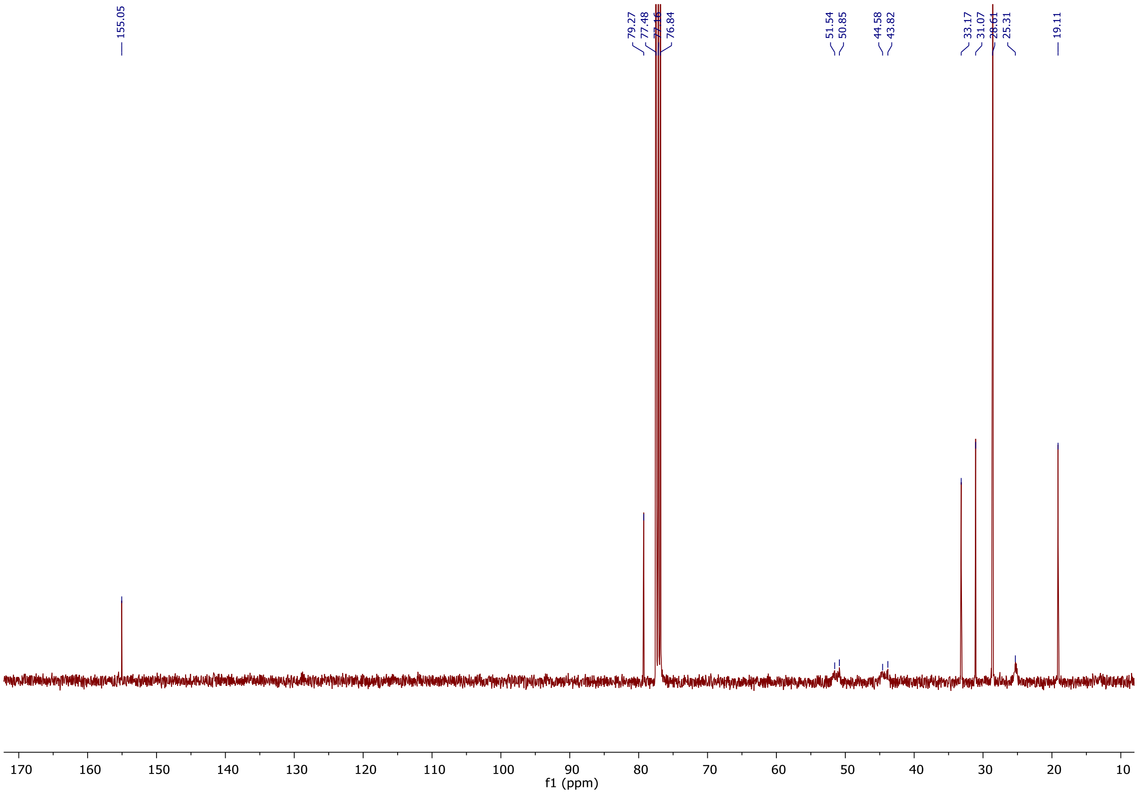

400 MHz ^{1}H NMR spectrum; 100.6 MHz ^{13}C NMR spectrum; $CDCl_3$

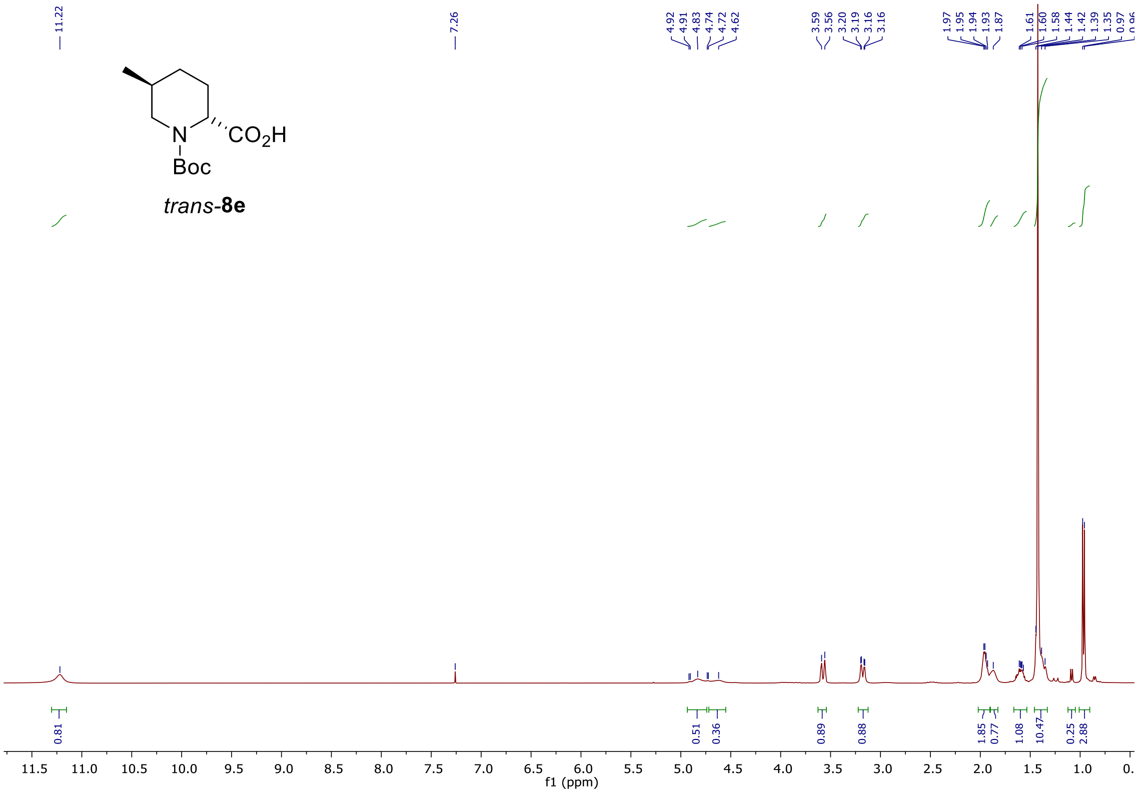

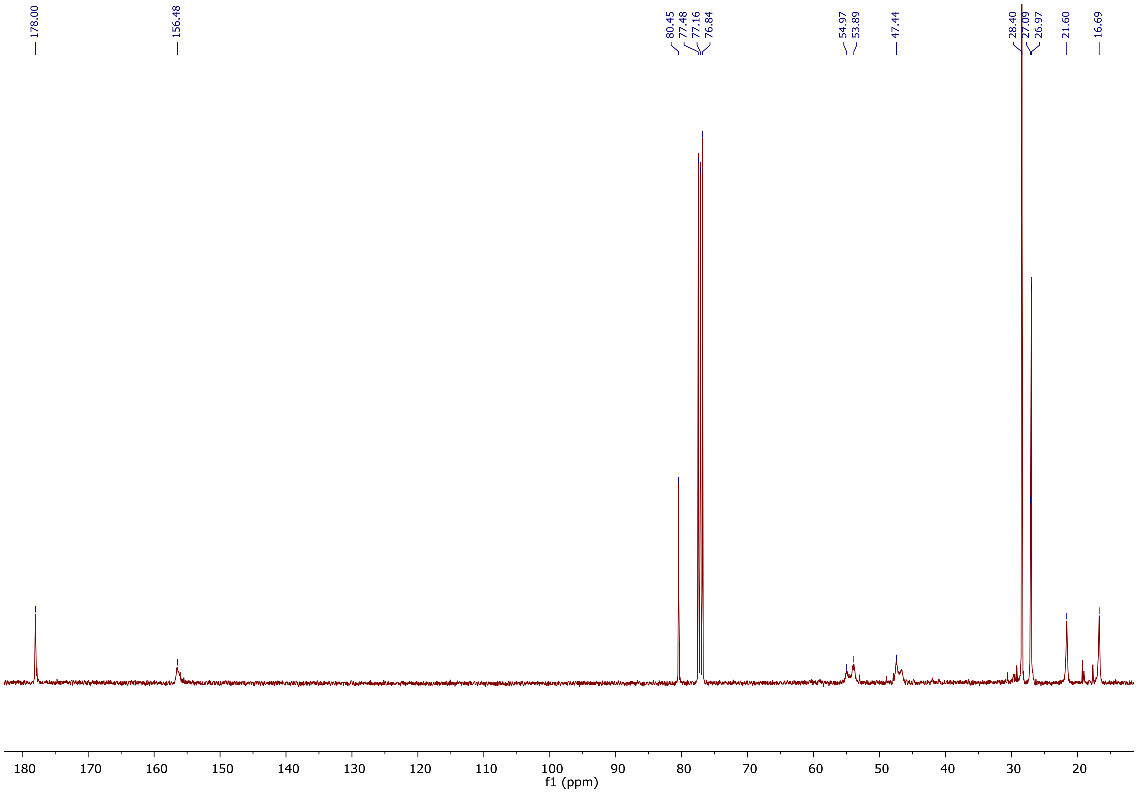

400 MHz ^{1}H NMR spectrum; 100.6 MHz ^{13}C NMR spectrum; $CDCl_3$

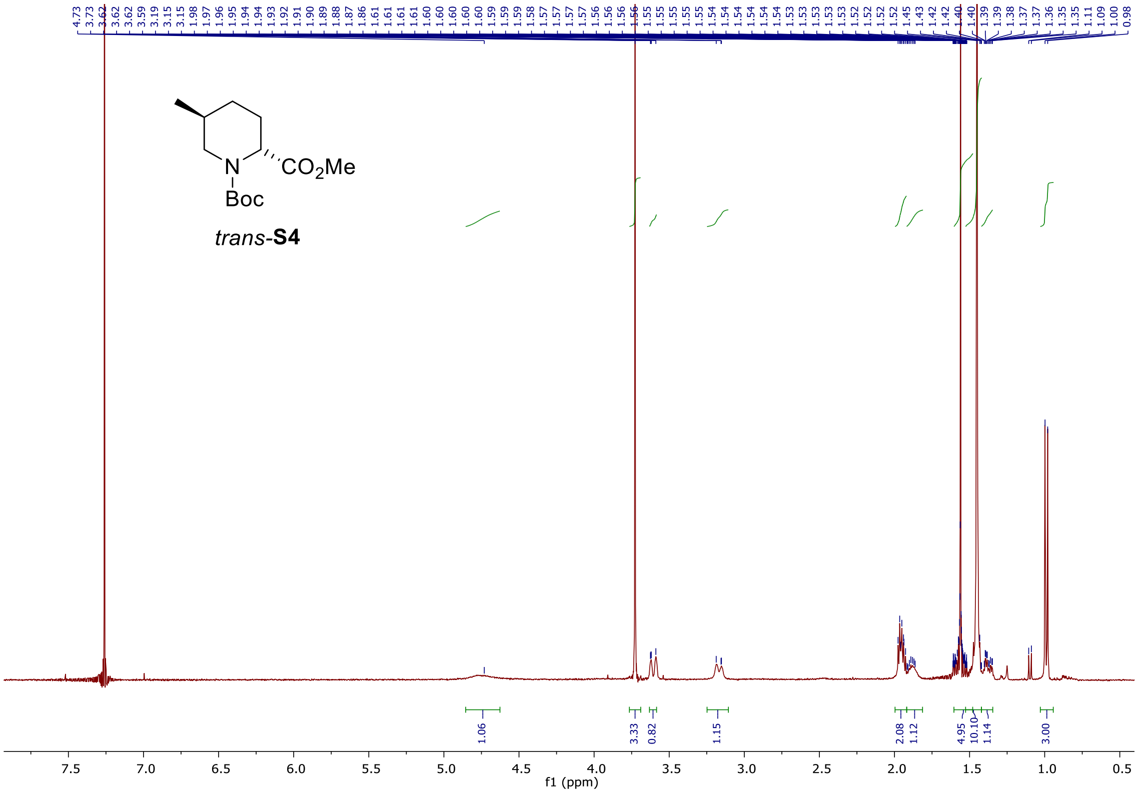

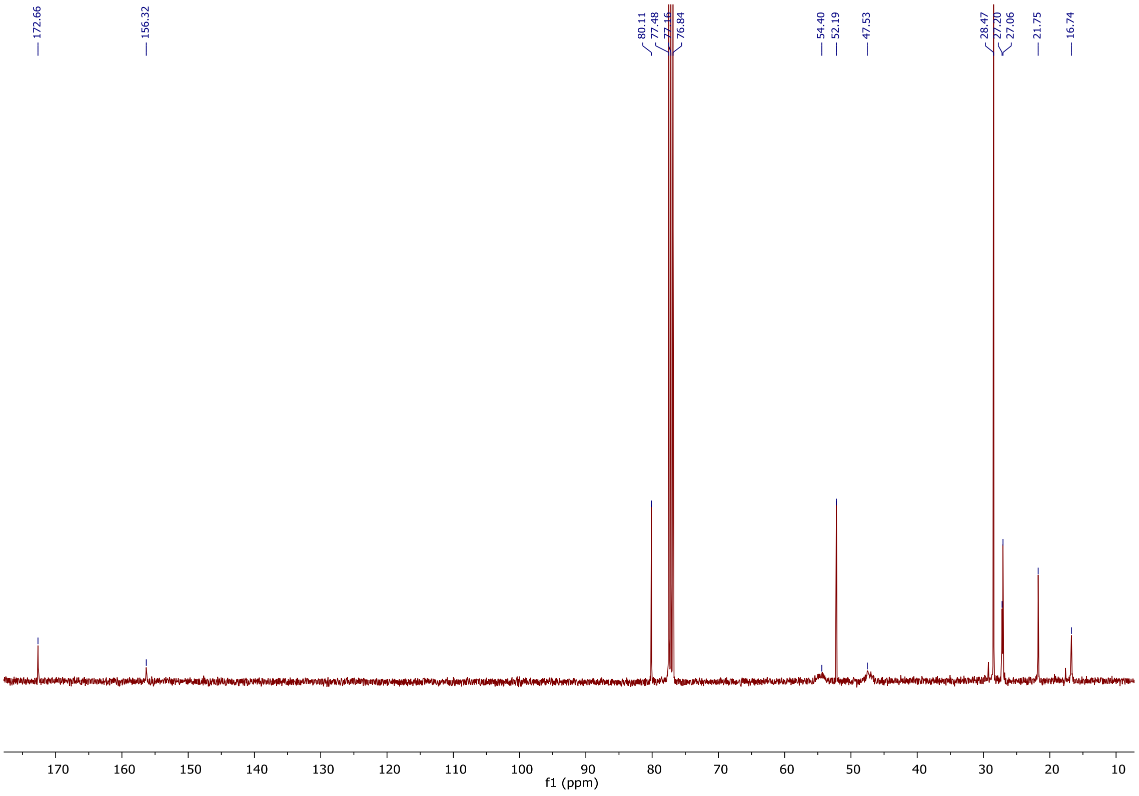

5. References

1 C. Agami, L. Dechoux, C. Ménard and S. Hebbe, *J. Org. Chem.*, 2002, **67**, 7573–7576.

2 P. O'Brien, T. D. Downes, S. P. Jones, H. F. Klein, M. C. Wheldon, M. Atobe, P. S. Bond, J. D. Firth, N. S. Chan, L. Waddelove, R. E. Hubbard, D. C. Blakemore, C. De Fusco, S. D. Roughley, L. R. Vidler, M. A. Whatton, A. J.-A. Woolford and G. L. Wrigley, *Chem. Eur. J.*, 2020, **26**, 8969–8975.

3 J. R. Henry, WO2016077161A1, 2016; Eli Lilly And Company

4 J. R. Medina, C. J. Becker, C. W. Blackledge, C. Duquenne, Y. Feng, S. W. Grant, D. Heerding, W. H. Li, W. H. Miller, S. P. Romeril, D. Scherzer, A. Shu, M. A. Bobko, A. R. Chadderton, M. Dumble, C. M. Gardiner, S. Gilbert, Q. Liu, S. K. Rabindran, V. Sudakin, H. Xiang, P. G. Brady, N. Campobasso, P. Ward and J. M. Axten, *J. Med. Chem.*, 2011, **54**, 1871–1895.

5 A. R. Renslo and R. L. Danheiser, *J. Org. Chem.*, 1998, **63**, 7840–7850.

6 E. Lorthiois, I. Marek and J. F. Normant, *J. Org. Chem.*, 1998, **63**, 566–574.

7 I. Prat, D. Font, A. Company, K. Junge, X. Ribas, M. Beller and M. Costas, *Adv. Synth. Catal.*, 2013, **355**, 947–956.

8 K. Hung, P. W. R. Harris and M. A. Brimble, *J. Org. Chem.*, 2010, **75**, 8728–8731.

9 C. Alegret, F. Santacana and A. Riera, *J. Org. Chem.*, 2007, **72**, 7688–7692.

10 F. Tjosås, N. M. Pettersen and A. Fiksdahl, *Tetrahedron*, 2007, **63**, 11893–11901.

11 J. Guo, L. Yan and W. Jin, *Heterocycl. Commun.*, 2015, 21, 203.

12 L. D. Julian and J. F. Hartwig, *J. Am. Chem. Soc.*, 2010, **132**, 13813–13822.

13 Z. Zhang, G. M. Giampa, C. Draghici, Q. Huang and M. Brewer, *Org. Lett.*, 2013, **15**, 2100–2103.

14 I. Colomer, C. J. Empson, P. Craven, Z. Owen, R. G. Doveston, I. Churcher, S. P. Marsden and A. Nelson, *Chem. Commun.*, 2016, **52**, 7209–7212.

15 https://llama.leeds.ac.uk/, (accessed June 2022).

16 A. Nadin, C. Hattotuwagama and I. Churcher, *Angew. Chem. Int. Ed.*, 2012, **51**, 1114–1122.

17 R. G. Doveston, P. Tosatti, M. Dow, D. J. Foley, H. Y. Li, A. J. Campbell, D. House, I. Churcher, S. P. Marsden and A. Nelson, *Org. Biomol. Chem.*, 2015, **13**, 859–865.

18 M. Lüthy, M. C. Wheldon, C. Haji-Cheteh, M. Atobe, P. S. Bond, P. O'Brien, R. E. Hubbard and I. J. S. Fairlamb, *Bioorg. Med. Chem.*, 2015, **23**, 2680–2694.

19 D. J. Foley, R. G. Doveston, I. Churcher, A. Nelson and S. P. Marsden, *Chem. Commun.*, 2015, **51**, 11174–11177.
